# Study protocol of “From Science 2 School”—prevalence of sports and physical exercise linked to omnivorous, vegetarian and vegan, diets among Austrian secondary schools

**DOI:** 10.3389/fspor.2022.967915

**Published:** 2022-09-28

**Authors:** Katharina C. Wirnitzer, Derrick R. Tanous, Mohamad Motevalli, Georg Göbel, Gerold Wirnitzer, Clemens Drenowatz, Gerhard Ruedl, Armando Cocca, Werner Kirschner

**Affiliations:** ^1^Department of Research and Development in Teacher Education, University College of Teacher Education Tyrol, Innsbruck, Austria; ^2^Department of Sport Science, Leopold-Franzens University Innsbruck, Innsbruck, Austria; ^3^Research Center Medical Humanities, Leopold-Franzens University Innsbruck, Innsbruck, Austria; ^4^Department of Medical Statistics, Informatics and Health Economics, Medical University Innsbruck, Innsbruck, Austria; ^5^Adventurev & change2V, Stans, Austria; ^6^Division of Sport, Physical Activity and Health, University of Teacher Education Upper Austria, Linz, Austria

**Keywords:** child, student, adolescent, puberty, physical activity, plant-based, healthy lifestyle, preventative health

## Abstract

The increasing prevalence of unhealthy lifestyle choices contribute to almost all chronic conditions negatively affecting individual and public health. As the most beneficial preventative solution, a healthy lifestyle focusing on the dual approach of physical activity (PA) and a healthful diet is highly recommended. Considering the growing number of people interested in sustainable, plant-based diets, it seems crucial to analyze lifestyle behaviors with a special focus on diet type to delve deeper into the unenthusiastic health status among young populations. Therefore, this multidisciplinary study aims to survey and scale health behaviors with a special focus on the prevalence of traditional and vegetarian diets in connection with PA levels among Austrian pupils (10–19 years), teachers and principals in secondary education levels I and II. Following a cross-sectional design, sociodemographic and school-related data along with a complete profile of lifestyle behaviors, including detailed information regarding diet, PA, sports & exercise, and other health-related behaviors, were collected using online-based questionnaires. A total number of 8,845 children/adolescents (~1.2% of the eligible 771,525 Austrian secondary school pupils) and 1,350 adults (~1.5% of total eligible 89,243 Austrian teachers/principals) participated in the study. As this is the first investigation to explore the prevalence of veganism/vegetarianism amongst a large group of pupils, the present study will add an important contribution to overcome the lack of knowledge on PA, sports & exercise linked to healthy alternative diets. With a sustainable healthy lifestyle, a healthy transition from childhood to adulthood occurs, which can result in growing healthier functioning generations at all social levels. As a study protocol, the present article is intended to present comprehensive details of the study design, objectives, and the associated analytical procedures of the “From Science 2 School” study.

## Introduction

Healthy behavior is—for good or worse—key in the development of lifestyle-related including non-communicable, diseases (NCDs) (1–6). Developing chronic diseases such as cardiovascular disease, diabetes, cancer and their risk factors (e.g., overweight, inactivity) can begin as early as in utero (7) but may also develop over the lifespan and poses the greatest threat to health and wellbeing (8–11). There is consensus that health behavior develops during childhood and adolescence and tracks over time into adulthood and over the lifespan to old age (2, 6, 12–19).

NCDs are accountable for 71% of deaths worldwide, with most cases preventable and even reversible (4, 5, 20). Physical exercise and diet are good predictors of mortality, with physical inactivity 4th, and overweight/obesity 5th ranked among the leading risk factors for global mortality accounting for 6 and 5% of premature deaths (hypertension: 13%, tobacco substance use: 9%, high blood glucose: 6%), respectively (6, 21–23). All the more, Western and developing nations face two global-scale health dilemmas of pressing concern with the greatest urgency for adults, younger populations (increasingly so), and health care systems worldwide: insufficient physical activity (PA), otherwise known as physical inactivity (6, 18, 22, 24–33), and overweight/obesity (2, 11, 21, 34, 35). According to the current Health Behavior of School-Aged Children (HBSC) study, 81% of children and adolescents fail to meet the recommended level of daily PA (60 min), and 21% are overweight/obese (26). This finding results partially from insufficient nourishment of nutrient-rich foods (48% of adolescents neither eat vegetables nor fruits), along with inadequate daily fruit and vegetable consumption (60 and 62%, respectively), whereby most children and adolescents fail to meet the current dietary recommendations (26, 29, 30). In Austria, a significant proportion of children and adolescents currently face greater risks for developing NCDs or lifestyle-related consequences (26, 29, 30).

Health is one major topic for human development and the future in education–matching the UN Sustainable Development Goals (SDGs, number 3 “Good Health and Well-Being” and number 4 “Quality Education”), the WHO Voluntary Global Targets on NCDs (especially number 3 “10% reduction in insufficient PA”) and UNESCO “Cross-cutting key competencies”– and is the learning objective for educators and policymakers to aim at and consolidate into education and curricula (3, 6, 36–38). Considering the various populations, including families, children and youth, health and education experts, academia and political authorities, amongst others, which specifically require targeting to strengthen younger generations, there is an overarching responsibility for health across the SDGs, with the recommendation to include major participatory input at all levels of society (39). The concept of health education raises the claim of holistic personality development against the background of health-oriented action competence and sustainable willingness to act (6). Schools and their settings provide a suitable environment for health behavior interventions and for developing health behaviors (40–46), which can influence pupils' lifestyle choices independent of their socio-economic backgrounds, where children and adolescents spend 40% of their wake time (40–46). Schools are one of the most promising public health settings since they reach a large number and variety of children and adolescents and create the foundation for shaping healthy behaviors for the most decisive life stages - childhood and adolescence. It has been documented that health policies without school health are doomed to fail (47–49).

In Austria, since 1997, the basic principle decree on health literacy and health education mentions that the number one of its primary goals is to design the school with an environment that promotes health in everyday school life (50). According to the Austrian state educational mandate of the curricula for secondary levels I and II (51–56), health promotion and health education are within the scope of the overarching education goal in the educational area “Health and Exercise,” and thus, are key for a sustainable teaching-learning process. Therefore, as health promotion is a leading learning objective of didactical interventions, health pre-dominates the overriding educational goal and is therefore relevant in all compulsory subjects and courses. However, in terms of implementation and application in everyday school life, health promotion is seen primarily as a special task of school sports, which gives the compulsory subject of “Physical Education” has the “leading role” (51–56).

Due to their positive health effects, PA, sports & exercise are not only sound recommendations to promote and maintain health over the life course but are recognized as “medicine” (1, 13, 14, 32, 57–60)—especially during the COVID-19 situation (61–66). However, it is well-accepted that exercise alone is not enough to promote sustainable health; thus, integrating lifestyle factors (PA, sports & exercise connected to diet) leads to superior health benefits due to cumulative effects (1, 18–20, 33, 60, 67–75). Healthy foods and especially plant-based diets were shown to be highly effective in promoting and maintaining individual health at all stages, including pregnancy, lactation, infancy, childhood, adolescence, adulthood, as well as athletes (33, 76, 77), with enhanced benefits over the lifespan (74, 76–82) and tremendous effects on the prevention and treatment of certain diseases (e.g., NCDs and others) (2, 47, 83). Therefore, vegetarian and vegan diets and whole plant foods are especially sound recommendations and even recognized as “medicine” (76, 80, 83–88), even more so when applied to severe health conditions and may protect against moderate-to-severe COVID-19 infections (64, 89–94). Available data show that 10% of Europeans (75 million people) adhere to different vegetarian diets (95), with 9.5% (845,000) of the Austrian population eating vegetarian or vegan (96) as well as 10.4% of young people in Germany (97). Moreover, vegetarian and vegan lifestyles are especially relevant for the peer groups of younger generations (i.e., young adults aged 22–38 years) (98) as the main drivers for the global avoidance of meat with an increasing trend toward plant-based diets (99, 100). For example, 25% of the 18 yr-olds in the UK eat vegetarian or vegan, while 30% of those aged 18–24 have considered to eat vegan or are already vegan (101, 102). Furthermore, 44% of young people (< 24 years) rate the vegetarian and vegan lifestyles as cooler than smoking (103), and 1 in 12 parents in the UK (8.3% of *n* = 2,200) raise their children (0–12 years) on a vegan diet citing health benefits (61%) followed by ethical reasons (35%) as their main motivations (104). According to a recent study analyzing nearly 2 billion Facebook postings from 132 countries (age group 15-65+ years) regarding interest in sustainable (low-carbon) diets and lifestyles, 12% of Austrian social media users were interested in vegetarian-vegan diets (105) with the plant-based movement forecasted to keep growing (99–106).

Investigating lifestyle behaviors, at least the dual approach of two lifestyle factors (e.g., PA, sports & exercise connected to diet)—clearly discriminated by kind of diet types and their interactions with sociodemographic aspects (e.g., sex, age, Body Mass Index (BMI))—seems to be crucial to advance our understanding of the unenthusiastic health status of young populations. While there is information on young people's PA behavior in Austria (26, 29, 30), there is no information on current dietary trends, especially vegetarian, and vegan diets in Austrian children and adolescents. The current data on diet type of Austrians is limited to adults and the general social context only. In particular, information on the prevalence of traditional and plant-based diets among pupils and adults (teachers, principals) within the school context is lacking.

## Objective

The aim of the interdisciplinary school study *From Science 2 School: Sustainably healthy – active & veggy* is to sample and scale for the first time the prevalence of traditional (omnivore) and alternative diets (vegetarian, vegan) in connection with various lifestyle habits among Austrian pupils (age 10–19 years), teachers and principals in secondary education levels I and II. Accordingly, the following goals have been specified:

1. The primary goal of this school study is to survey the prevalence of omnivorous, vegetarian and vegan diets linked to PA levels of pupils and teachers/principals;
2. The secondary goal is to investigate the health behavior of children and adults at secondary schools;
3. The final goal is to validate the participants' self-reports on their dietary behavior.

*From Science 2 School* is the first study to survey the relationship between PA, sports, exercise and diet type in the school context by taking a large sample into account. In addition, the present study aims to contribute to the inadequate data currently available on adult populations regarding nutritional trends; however, the particular focus is the lack of information on nutrition trends in children and adolescents (10–19 years). Based on the solid ground of the body of science, population and cohort studies have shown increased health consciousness and a healthier lifestyle among vegetarians and especially vegans compared to omnivores. This link is due to avoiding unhealthy practices and taking the time for rest and relaxation with the vegans, in particular, being significantly more physically active, being more likely non-smokers and less likely to drink alcohol (76). Therefore, it was hypothesized that vegetarians, but especially vegans in both the pupils and teachers/principal samples, are more engaged in PA, sports and exercise. This work is necessary to expand both the epidemiological and didactical knowledge for children and adolescents but also adults, especially within the educational setting.

The results of this study will provide a major reflection by adding novel evidence to the body of science regarding the prevalence and characteristics of vegetarians and vegans, in particular, including a special focus on the permanent linkage to PA, sports & exercise in the school context of secondary education. The findings are expected to empower schools to design future offerings that consistently combine PA, sports, exercise with healthy food choices, meals and dishes across the school environment (e.g., the canteen and catering, vending machines, interdisciplinary events, etc.). Thus, the data can help to:

1. Justify the need to consider this interdisciplinary but basic dual approach as a highly effective, safe and low-cost intervention to improve pupil's health by evidence-based rationale;
2. Encourage policy and decision-makers in education (as federal/governmental authorities, principals, teachers and families) to evaluate the current health-related offers in school settings and create or even evolve programs, measures or materials concerning this simple dual approach and to put it into action in everyday school scenarios in order to;
3. Develop health-orientated action competence and sustainable action readiness relating to pupils' health through competence-orientated education and help achieve better pupil health.

Therefore, this study ultimately intends to help transfer the future findings into the school context of school life and school environment and to translate into health-orientated actions and sustainable health-related action readiness, and thus promote the health-related skills and health literacy of pupils required (regardless of their socioeconomic background) to manage their health and maintain it in the long term through competence-orientated education. Moreover, the findings of this study might be useful to:

- Establish the dual approach for sustainable health as a minimum recommendation within school health promotion as an educative, teaching and research goal in line with the state mandate;
- Transfer scientific data more directly to the public by first addressing pupils at the secondary school settings and close the circle by improving both individual and public health status;
- Recommend this safe, effective, low-cost tool to health experts, decision-makers and multipliers to implement in everyday scenarios also as an extension of the school setting (e.g., family, community, therapists, family/primary care and specialized physicians, nutritionists, sports experts, and coaches).

## Materials and methods

### Study design

*From Science 2 School* was conducted as an Austria-nationwide study using a cross-sectional design and based on a multiple-level cluster sampling approach. The present study is an interdisciplinary project incorporating Sport Science, Nutrition Science, and Health Science.

### Participants

**Figure 1:**
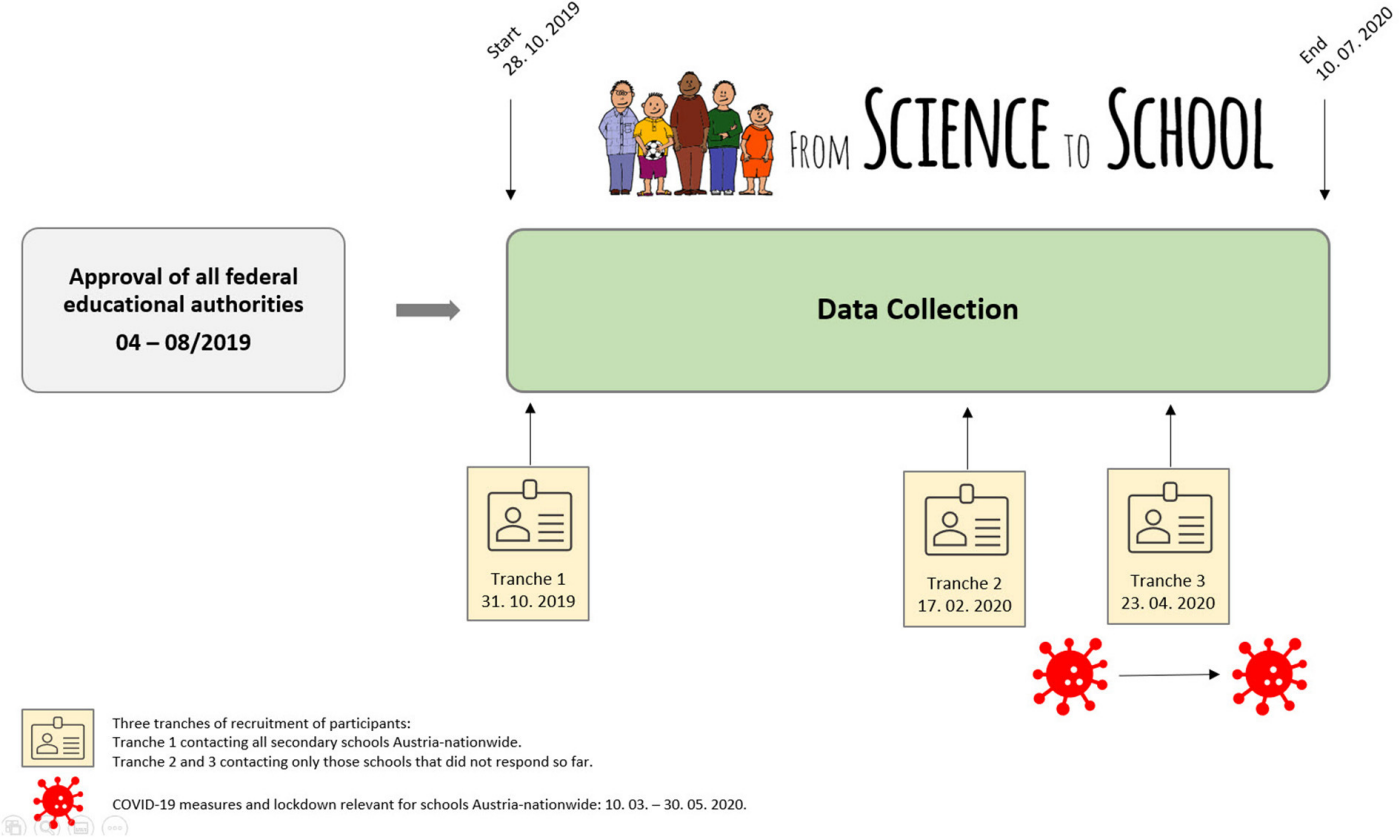
Flow chart of procedure and timescale of previous application for approval by educational authorities and subsequent data collection by online survey.

This study intended to involve the largest possible number of study participants in order to obtain meaningful results based on a large data set (for recruitment strategy, see Figure 1). The target group was all Austrian pupils of secondary levels I and II and all teachers and principals anchored in their respective academic-professional roles at a secondary level I and/or II school. Resulting from this, the Austria-nationwide basic sample size is 860,768 people total and made up of 771,525 pupils and 89,243 teachers/principals at secondary school level.

With the start of data collection (28, October 2019), all schools that serve as secondary levels I and/or II in Austria (*n* = 2,688) were contacted via email to participate in the study. Regarding the implementation of the survey, the study participants were contacted as follows: (1) The school administration received information about the goal and the procedure via email (including an information letter for parents/guardians, a cover letter for teachers and a web link to the online survey) and by personal communication (e.g., telephone call) and (2) gave the resources to the class directors to survey the pupils in daily classroom sessions and to all teachers to survey all colleagues in the teaching staff of the respective school, whereby the principal himself/herself participated in the survey of the teaching staff.

**Figure 2:**
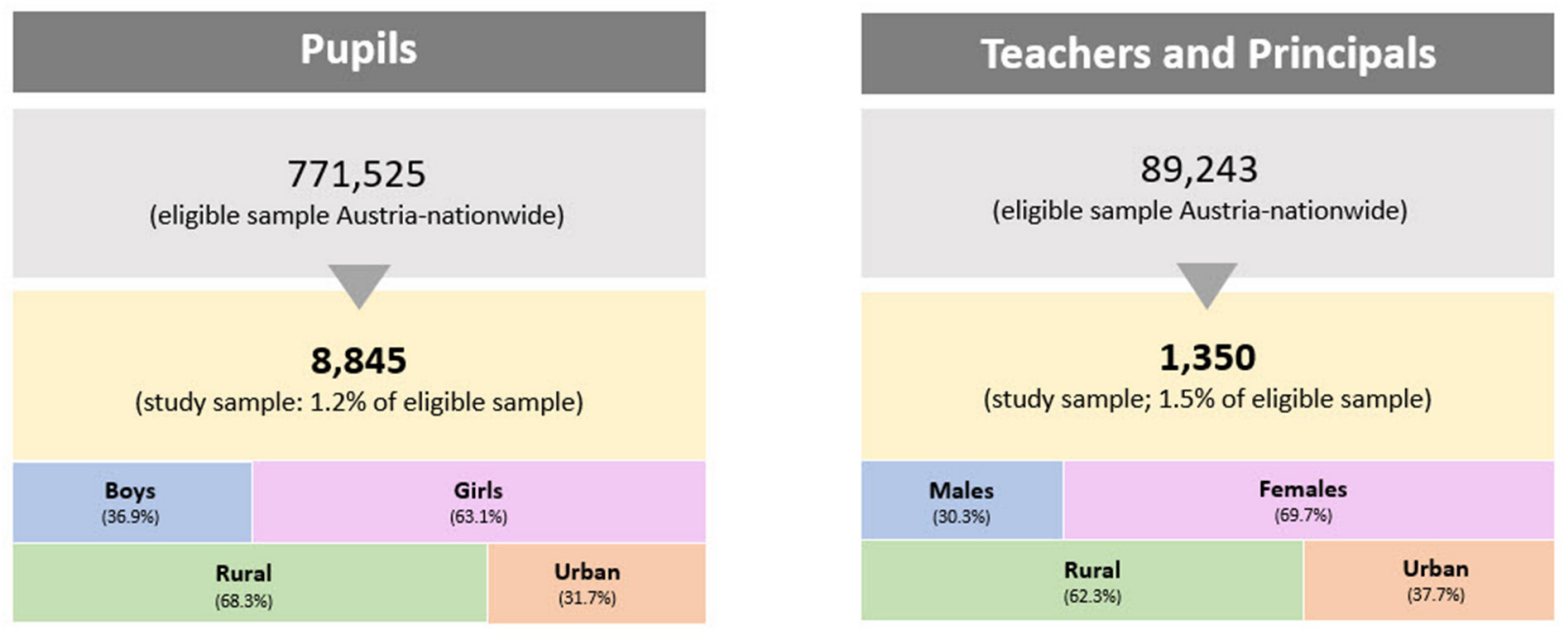
Flow chart of participants' enrollment and classifications of pupils and adults by sex and living environment.

After the closure of data collection (10, July 2020), 8,845 pupils (1.2% of the total eligible 771,525) and 1,350 adults (1.5% of 89,243 eligible teachers, principals) had participated in the online survey (Figure 2).

### Inclusion criteria

Any participant who attended school or was employed in an official educational capacity could participate, meaning pupils and their teachers and principals of Austrian secondary school levels I and/or II. For successful participation in the school survey, a complete data set consisting of a comprehensively filled-out questionnaire (1) and the written declaration of consent and publication (2) was required.

### Questionnaire

During data collection, the short standardized online questionnaire was available in German (LimeSurvey, version 3.17.16). The participants accessed the questionnaire provided for them and completed it via an encrypted interface. The survey was available online, could be taken in school or at home, and took ~10 min to complete with access to a mobile device, tablet or PC/laptop.

The survey of this school study was based on self-report or self-assessment and was provided in two different versions (inclusive respective web links), with one exclusively for pupils (children and adolescents; see Appendix Material A.1) and another for teachers and headmasters (adults; see Appendix Material A.2): www.science2.school/en/#Questionnaire.

The self-report survey consisted of five parts with questions about the individual (part A), PA, sports & exercise (part B), nutrition (part C), health (part D), and miscellaneous (part E) (32, 103, 107–124). In particular, data collected included the following information: demography (nationality, age, sex, federal state, place of residence and region); biometric data (height, weight, calculated BMI); the school context, such as professional role (pupil, teacher, principal), school level (lower or upper secondary level), school type; current dietary adherence, motivation, and duration of current diet, daily fluid intake; current physical exercise behavior (duration/day, frequency/week, type of sport, social, and organizational form, competition participation); motivation and genesis for specific lifestyle choices. In addition, control questions on both nutritional patterns and PA habits were included in the online questionnaire (both versions) to identify conflicting data in order to obtain the most reliable data possible.

#### Questionnaire validation

**Figure 3:**
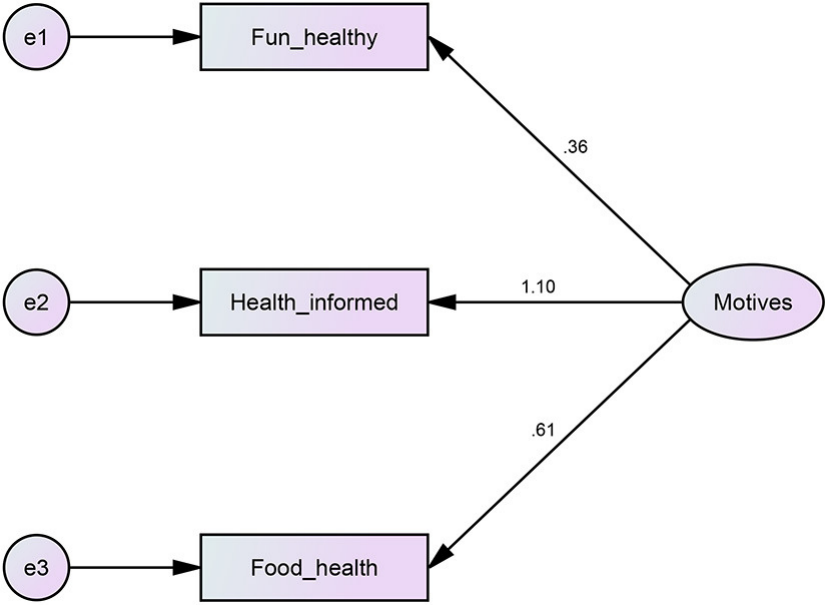
Flow chart of the confirmatory analysis for the tested model (AMOS 22 Software, IBM, Chicago, USA) with regard to the validation of the questionnaire.

The present survey was developed based on the relevant literature (32, 103, 107–123), particularly from validated questionnaires (111, 115, 116, 118, 121), well-known large-size studies/reports (113, 119–121) and recognized literature of renowned authors (122). Accordingly, participants were asked to respond mainly to single-choice items, multiple-choice items, and their preferences among several options. Due to the nature of the items (individually analyzing participants' preferences/life choices rather than latent constructs) and the fact that they were obtained from previously validated questionnaires rather than created originally, it was not possible to estimate content, construct, or criterion-related validity; instead, such items should be considered separately when analyzing and interpreting the data. Despite the majority of the items, including those on nutrition, behaviors, and exercise preferences having such a singe-item structure, three questions in part A, section 11 (Appendix Materials A.1, A.2) assessed different motives for exercising using a 4-point Likert scale from 1 (totally true) to 4 (totally false). Due to the characteristics of these items, the possibility of a latent variable on exercise motives was examined as it may result in presenting said items as a single global dimension. Although results from exploratory factor analysis suggested a 3-item structure is statistically possible (KMO = 0.589; Bartlett's Test of Sphericity > 0.001; total variance explained = 61.5%; item factor loading ≥ 0.628), internal consistency was only sufficient (alpha = 0.636). Further confirmatory analysis carried out with AMOS 22 software (IBM, Chicago, USA) for the tested model (Figure 3) delivered insufficient outcomes (χ^2^ = 0; *df* = 0). In such cases, the indexes of goodness of fit should not be considered and the items should be considered separately (120). As a consequence, a specific validation is not required for these three items; instead, each one provides valid and unique information on participants' motives for being active.

### Procedure and trial status

Figure 1 shows the procedure and timescale of the priorly approved application by the educational authorities and subsequent data collection by online survey. Regarding the operation of implementation, two initial steps were required: (1) Approval of the questionnaire to implement the survey directly at the schools from all nine Austrian federal states by the respective state school boards of the federal education authorities; (2) approval by the Federal Ministry of Education, Science and Research to facilitate contact with the participating schools and to increase the response rate for data collection to reach a representative data set. Recruitment of participants was performed in three phases: phase 1 aimed to contact all secondary schools Austria, nationwide followed by phases 2 and 3, which involved only those secondary schools that did not respond to participate with their school location. Data collection was open from October 31st, 2019 through July 10th, 2020. All questionnaires are now closed.

### Outcome measures

#### Primary outcome measures

The primary outcome measures are the prevalence of diet types (omnivorous, vegetarian, vegan), the prevalence of levels of PA, sports & exercise, as well as the linkage of kind of diet and activity level among Austrian pupils and adults (teachers, principals) of the secondary levels I and II.

#### Secondary outcome measures and effect modifiers

In addition, further outcome measures considering pupils include sex; age; BW; height; calculated BMI; residence (urban vs. rural); school level and class; type of school; time of adherence, motivation, and genesis for current diet; time of adherence, motivation, and genesis for current recreational PA, sports & exercise behavior (duration/day, frequency/week, type of sport, social, and organizational form, competition participation, membership in a sports club); daily fluid intake; daily intake of fruit and vegetables; intake of specific foods/food groups; food allergies/intolerances; healthy and health-promoting foods; healthy and health-promoting PA, sports & exercise (frequency, intensity, duration); leisure time activities; main factor affecting health; smoking; alcohol; lifestyle.

Secondary outcome measures for adults include: sex; age; BW; height; calculated BMI; residence (urban vs. rural); the time of service/duration of the profession; activity/teaching in which school level and type; extent of employment (full/part-time); weekly working time; time of adherence, motivation, and genesis for current diet; time of adherence, motivation, and genesis for current recreational PA, sports & exercise behavior (duration/day, frequency/week, type of sport, social and organizational form, competition participation, membership in a sports club); daily fluid intake; daily intake of fruit and vegetables; intake of specific foods/food groups; food allergies/intolerances; healthy and health-promoting foods; healthy and health-promoting PA, sports & exercise (frequency, intensity, duration); leisure time activities; main factor affecting health; smoking; alcohol; lifestyle.

Based on a wide variety of study questions and subsequent hypotheses, potential interactions and associations between the aforementioned variables at different study levels will be analyzed subsequently at the end of data collection. Specific study biases and limitations will be considered where applicable during interpretation of results. Findings will be prepared and reported in the form of original scientific articles in the upcoming years.

### Ethical principles

The study was conducted according to the medical professional codex, the Helsinki Declaration as of 1996, Data Security Laws and good clinical practice guidelines. Study participation was voluntary and could be canceled at any time without provision of reasons or negative consequences.

#### Written informed declaration of consent and publication

Information on the survey was available on the official website and set up for this purpose. The participants were briefed that participation was voluntary, the survey was anonymous, confidential and processed exclusively for scientific purposes. Prior to the study, participants received written information about the content and procedure of the planned study and gave their written consent online to participate in the survey, prior to publication of the study results. If the respondent discontinued participation in the study, the respective data records were deleted.

#### Vote of the ethics committee of the educational authorities

The present Austrian-wide study is supported by the Federal Ministry of Education, Science and Research, Department 1/7 – School and University Sports. The study protocol was approved by the Rectorate of the University College of Teacher Education Tyrol ethics board (PHT-HSa-17-Z1.8-5n_2427; September 21, 2019) in accordance with the ethics board of the nine Austrian Federal Education Authorities, which was required in order to contact the schools. The final mandatory step to get permission to start the study and conduct the survey in the school setting was the approval by the respective principals at 2,688 schools of secondary levels I and II all over Austria.

#### Duties on part of the investigators

The authors confirm that the ethical and scientific criteria and the quality standards in terms of study planning, procedure (inclusive monitoring), analyses and documentation will be carried out completely in accordance with the study protocol. All rights of the participants will be respected, and the results of the study will be conducted appropriately. The investigators will report and document deviations to the government and federal educational authority ethics boards.

#### Evaluation of the benefit-risk ratio

Participation in the study was voluntary, did not involve any additional risk for the respondents and was reasonable and sensible from a didactic-pedagogical point of view. The survey could be terminated at any time without consequence. Participation in the study was without any financial compensation.

### Data security

All data will be treated in accordance with the applicable data protection regulations and data security laws. The data was collected and is stored anonymously, which is in line with the requirement of anonymous data collection by all the nine federal education authorities. No IP addresses of the participants were stored in the questionnaire or database. Therefore, the assignment of IP addresses or any personal information of participants of the survey was not possible, and no further step toward anonymization is necessary.

### Power analysis and representatively

The results of this study will be presented following the guidance of the STROBE and/or CONSORT criteria (https://www.strobe-statement.org/?id=available-checklists, http://www.consort-statement.org/) mentioned in detailed statements for e.g., kind of publication.

#### Strata, study groups

Primarily, age (10≥14≥19 years) and sex (female, male) were used for estimating sufficient case numbers. The collected data will be representative for sex and the two age groups on a 95%-CI level (with a margin of error determined accordingly: ME = 0.025). Comparison between groups will be made using the appropriate chi-square contingence-test with a level of significance of 0.05 and a power of 80 % (effect size d^2^ = 0.003).

For statistically reliable and representative results of any one particular variable, a sample size of *n* = 984 was needed in order to detect a 3% difference between dietary subgroups (mixed vs. vegetarian vs. vegan) and to reach 80% power within a two-sided test (95%-CI) with an alpha value of 0.05. For linking two or three variables, a respective sample size of *n* = 1,968 and *n* = 2,952 was necessary for dietary subgroup comparison in pupils (e.g., age 10–14 years × sex female; or age 10–14 years × sex female × type of school).

#### Calculations of case number scenarios

Data cube: For each variable (or stratum), one axis has to be considered, e.g., for two strata (e.g., age, sex), two axes and six cells have to be considered, and for each cell, the enclosed case number estimation is relevant. Confidence interval scenarios for proportions were performed using normal approximation (n large) (nQuery). Table 1 shows calculated case number scenarios for potential associations between diet type and health indicators (e.g., status of BW/BMI_CALC_).

**Table 1:** Calculation of case number scenarios for study groups.

	**1**	**2**	**3**	**4**	**5**	**6**	**7**	**8**	**9**
Confidence level	0.950	0.950	0.950	0.950	0.950	0.950	0.950	0.950	0.950
1- or 2-sided interval	2	2	2	2	2	2	2	2	2
Expected proportion (%)	0.200	0.200	0.200	0.300	0.300	0.300	0.500	0.500	0.500
Distance from proportion to limit	0.010	0.020	0.025	0.010	0.020	0.025	0.010	0.020	0.025
Number per cell per stratum	6147	1537	984	8068	2017	1291	9604	2401	1537

Explanation for Scenario 3: For the estimation of a proportion of 20% (two sided 95%-CI) ± 2.5% accuracy, a sample size of *n* = 984 was required after data clearance. If a higher accuracy is needed, the case number is *n* = 1,537 (accuracy of 2%) or *n* = 6,147 (accuracy of 1%). The power to detect an effect of 0.003 between three subgroups (e.g., P1 = 27 vs. P2 = 30 vs. P3 = 33%) would be 80%. The Software nQuery Advisor 7.0 was used for the case number estimation.

### Statistical methods and data analysis

Stratified subgroup analyses will be conducted on a descriptive level. The analyses will be performed using the latest version of statistical software according to the statistical methods corresponding to the available data. All results are reported as arithmetic means and standard deviations (SD) for metric variables or when more meaningful, median and interquartile range (IQR), and as absolute and relative values for categorical data. In addition to bar charts, several types of box plots will also be used for the graphic display. Multivariate regression analyses are used to calculate the effects of diet type, sex, age, BMI_CALC_, weekly exercise or sports level/frequency, etc. Analysis of variance is performed to identify individual differences in sex, age, BMI_CALC_, etc., as well as years of adherence to the particular diet type and sports experience, motivation for a specific diet type and sport, type/discipline of sport (social form, informal/formal, indoor/outdoor), participation in competitions, membership in sports clubs, additional sports, etc. The level of statistical significance was set at *p* ≤ 0.05.

## Discussion

This study is the first to survey the prevalence of vegan vs. non-vegan secondary school pupils with a dual approach to health. By taking a large sample into account, this study will provide an important contribution to the lack of information about plant-based diets linked to PA, sports & exercise.

Healthy children and adolescents form the foundation and prerequisite for healthy adults who could successfully serve as role models and decision-makers of the future (e.g., parents, teachers, physicians, therapists, politicians, etc.). The health behavior of children and adolescents is vital to assess because health behavior develops during immaturity, tracks over time into adulthood and later becomes increasingly difficult to change (2, 12–19, 125, 126).

Factually, children are the essence of a nation's future. As there is a consensus on enabling young generations to reach their full potential as a prerequisite for sustainable development, there is growing recognition of the importance of preventative measures at young ages based on (e.g.) lifestyle behaviors. However, children require adult support to grow up healthy and thrive as they are incapable of doing so alone (127, 128). Consequently, policy- and decision makers have a moral, social, and economic duty to ensure children's health (19). Therefore, the most important and influential time to learn good health behaviors is as early as possible in life and needs consistent attention until adulthood at the least (2, 6, 12, 17, 19).

Teacher health is not only a prerequisite for high-quality education but crucial for successful societies; healthy teachers positively contribute to educating and growing healthy children via their distinct impact on pupils' lifestyle choices (129, 130). The promotion of teacher health is thus not a “private matter” of individual teachers but a contribution to the education system and the general public as a whole (131) regarding public health issues for nations such as Austria (132). Good health behaviors, healthy lifestyles, and health education passing from adults to children appear to be part of the future health solution; however, parents or teachers cannot be relied upon exclusively for building the health of their children and preventing NCDs. Children generally have two main settings for naturally learning the basics of healthy lifestyles and thus the individual health behaviors based on lifestyle factors, such as the micro-unit family setting or the macro-unit school setting as general socio-economic (living/working) health domains that are more culturally/environmentally-oriented (133, 134), with the latter as a crucial age-appropriate environment being key to any future public health solution (6, 19, 47–49).

The school setting is ideal for encouraging healthy behavior (e.g., regular PA, healthy diet) at young ages and across various socio-economic backgrounds considering children and adolescents spend a long period of time (9 years in Austria) in compulsory schooling (135). In addition, school settings are well controlled regarding age groups (school level), state educational mandates/national curricula, and standardized teacher education at the tertiary level (University/College) (98, 136). Concerning any future sustainable health solution, the central prerequisite is a health-promoting school environment (6, 19, 47, 137–141). Therefore, health initiatives that exclude school settings are doomed to fail (48, 49, 142).

Consistently, a current review identified education amongst others as an effective public health intervention for improving child health in reducing morbidity as well as mortality from infectious diseases (143), indicating the key potential of educational settings (primary up to tertiary) to succeed in tackling the development of NCDs and their risk factors to improve the health of nations (18). Reversing current health risks is a common intervention strategy and has shown positive results in previous study designs (45, 46, 144, 145). However, the singular strategy to intervene after health risks occur is inefficient and often results in only short-lived beneficial changes (146). Thus, the dual approach of linking PA, sports and exercise with nutrition is crucial (18, 87).

PA, sports & exercise are necessary components of energy flux, well-known tools to reduce the prevalence of overweight/obesity, essential for developing and sustaining a healthy body weight and BMI (147) and, therefore, aid in preventing and improving major health crises (18, 21, 58). Promoting the development of PA, sports & exercise behavior in childhood has a direct influence on the prevalence of physical inactivity. Respective interventions that target activity levels in children and adolescents were shown to be feasible in this regard (145) and are quite clear regarding positive health effects: the more PA, sports & exercise young people take part in, the greater the benefits (44, 59, 144, 145, 148).

However, there seems to exist a limit to improving health with PA, sports & exercise alone since people who live active lives can still develop NCDs and chronic diseases (18). Severe atherosclerosis has been observed in young people and soldiers around the age of 20 and athletes, with 75–90% coronary artery blockage, which has a detrimental effect on the brain and peripheral extremity arteries (18, 147–153). Therefore, the additive impact of other lifestyle-related factors (particularly diet) may be decisive in NCD development. Meanwhile, the global obesity prevalence almost tripled from 1975 to 2016 (34), and overweight/obesity is associated with higher all-cause mortality across four continents (34); further, it has been reported that poor dietary behaviors are common across Europe (4, 5, 35). According to a recent report of the Global Burden of Disease Study (22), compared to other factors, dietary risks account for 22% of all deaths among adults (≥ 25 years) in Western countries, having the most impact with more than half of all diet-related deaths linked to low intakes of fruits and whole grains along with high intake of sodium. Children and adolescents of Western societies do not meet the consumption recommendations for fruits, vegetables, and whole foods.

According to scientific data, there appears to be a strong connection between plant-based diets and overweight/obesity, and thus, NCD prevention (76, 77, 82, 149, 150, 154–157). Due to the characteristics of whole plant-foods (low-calorie, high density of nutrients and dietary fiber) compared to animal foods (e.g., energy-dense, high intakes from protein, fat, salt, sugar, refined carbohydrates, but low in fruits, vegetables and dietary fiber) (18, 158), such diets provide the concise prerequisite for sustaining healthy body weight, since vegans followed by vegetarians, were found typically having the lowest BMI, with omnivores having the highest BMI (76, 150, 154–156). Although low BMI does not help determine whether an individual might be predisposed to chronic disease, a high BMI almost always does indicate such a predisposition (159). The characteristics of plant foods (low energy, high density of fiber, and nutrients) (16, 76, 83, 150), however, do not seem to be the only reason for a healthier BMI in vegan/vegetarian populations since data indicate that those who follow plant-based diets may have other healthy lifestyle habits in addition, such as higher engagement in sports and exercise (76, 141, 160). Therefore, based on scientific evidence available, vegan adults may have the lowest risk for becoming overweight/obese as a risk factor of NCDs since they were most often reported within healthy BMI categories and thus at lower risk for developing NCDs (76). However, even vegans may still consume high proportions of their daily calories from processed foods, and therefore it is plausible that plant-based diets can be unbalanced and unhealthy, too (18, 76).

Well-planned vegetarian and vegan (supplemented with vitamin B12) diets are healthy, nutritionally adequate, and appropriate for all age groups, including pregnancy and breastfeeding, infants, children and adolescents, adults and seniors, and athletes. Moreover, the Academy of Nutrition and Dietetics (161) explicitly emphasizes the health benefits of plant-based diets, especially in the prevention and treatment of certain chronic disease (161, 162). Health benefits of plant-based diets are well-documented, from lower body weight, BMI, blood pressure, low risk of obesity, type 2 diabetes, and certain cancers to increased antioxidant status (reduced oxidative stress) and reduced micro-inflammatory processes (76, 161).

Vegetarian children have also been shown to be leaner than non-vegetarian children, with these differences in BMI increasing during adolescence into adulthood, and reduced risk of developing overweight and obesity, high cholesterol, hypertension, heart disease and type 2 diabetes. Children on a full vegan therapy diet effectively reduced blood pressure, cholesterol levels, body weight (obesity), and risk for/status of chronic disease(s) (98, 161–163). Furthermore, the long-term positive influence of plant protein sources, as well as fruit and vegetable consumption, on health and high life expectancy is evident, especially in long-term vegetarianism (≥ 17 years: +3.6 years), as well as a significant reduction in all-cause mortality of up to−34% with a plant-based compared to a mixed diet (18, 57, 76, 98). Moreover, there is a consensus that nutrient deficiencies concerning so-called critical nutrients (e.g., iron, vitamin D, vitamin B12, iodine) occur in all dietary patterns (including mixed diets) since an undersupply or deficiency is usually caused by incorrectly implemented nutrition or inadequate planning and composition of meals (18, 98, 161, 164). As with any diet, the quality and reliability of nutrition depends on a person's individual nutritional knowledge and the availability and option of fortified foods (76). Plant-based diets per se are no more deficient than any other diet, although vegans should pay specific attention to appropriate intakes of vitamin B12 (76, 83, 98, 161). For daily recommended nutrient intake, mixed dieters were found to undersupply seven nutrients (calcium, fiber, folic acid, iodine, magnesium, vitamins C and E), while vegans did not reach the recommended intake of three nutrients (calcium, iodine, and vitamin B12). The micronutrient supply of vegans vs. non-vegans shows a good vitamin B12 status, no differences for vitamins B12, D, or iron, and no deficits for vitamins A, B1, B6, C and E, iron, magnesium, phosphorus, copper and folic acid, and at the same time, better intakes of beta-carotene, vitamins C and K, folic acid, magnesium, potassium, dietary fiber and phytochemicals (165–168).

In contrast to some national nutrition organizations, supported by the latest evidence, since 1997 the AND (161, 162) recommends well-planned vegetarian and vegan diets (supplemented with vitamin B12) as they meet the energy and nutrient requirements of infants, children and adolescents and promote for children's healthy growth and development (76, 83, 158, 161–163, 169–171). Therefore, such healthy options should be offered as early as possible-before chronic diseases develop (76, 98, 161).

Adults who frequently consume fruits and vegetables have consumed these foods since childhood (98). With no particular nutritional risks in the vegan subgroup (170), the VeChi-Youth study recently found a significantly higher intake of legumes, nuts, dairy and meat alternatives, with the intake of whole grain products being highest and that of fats/oils and sweet foods being lowest, in vegan children and adolescents in Germany (6–18 years), of while omnivores tend to consume fewer vegetables in favor of dairy and fats (169). In addition, the VeChi-Diet study (*N* = 430) comparing vegan vs. non-vegan 1–3 yr-olds in Germany even showed that vegan child nutrition is possible without deficiencies. The vegan diet provides the same amount of energy and macronutrients (158) but a better fat quality (highest average intake of polyunsaturated fatty acids and lowest intake of saturated fatty acids) than vegetarian and mixed children (171) in early childhood, which contributes to normal growth of vegan infants (158, 171). Vegan-fed infants showed a more favorable nutrient intake (e.g., highest average intake of vitamin E, vitamin B1, folate, magnesium, iron, vitamin B12 intake when supplemented) than omnivorously-fed children; however, the latter had higher intakes of vitamin B2, calcium and iodine, vitamin B12, and of the health-promoting omega-3 fatty acid DHA compared to plant-based children (171).

However, for more details on the detrimental effects and the benefits of vegetarian and vegan diets for adults, children and adolescents, active people and athletes, the interested reader is referred to two recent reviews on these specific topics (18, 98).

Regardless of diet type, assuring adequate consumption of all essential nutrients has been emphasized to prevent the risk of nutritional deficiencies and/or undersupply in the growth and development process (160). In this regard, daily fruit and vegetable intake and a balanced fluid intake/hydration status are considered key elements of any diet type (172, 173).

By separating distinct dietary subgroups (omnivore, vegetarian, vegan), *From Science 2 School* can identify possible health behavior associations related to diet type. To date, there is limited evidence on the prevalence of diet type in school children (29, 49, 50, 119, 159, 174), with no existing evidence from the school context and on Austrian pupils particularly. *From Science 2 School* addresses this issue for the first time in the school setting and will add to the lack of information on the diet type of pupils and teachers/principals related to physical activity and body weight as important health indicators (26, 29, 30, 158). Considering the depth of the scientific evidence related to the topic, the dual approach of sports and exercise linked with plant-based nutrition appears most promising for overall health (5, 18, 40, 41, 46, 87, 175–177).

However, interspersed into the background of the global health crisis and its underlying data, it appears that the general population, parents, and teachers, in particular, seemingly lack adequate knowledge or the qualifications for teaching competence-orientated health literacy to future generations.

### Practical implications

As a result of the mass movement toward plant-based diets, consumers are increasingly demanding meat-free and purely plant-based meals, so that supermarkets, catering and public catering establishments (snack bars, fast food, restaurants, hotels) now offer a wide range of vegetarian-vegan options (98).

*From Science 2 School*, unlike previous designs, is based on the Austrian state mandate (50–52) and therefore includes a “prevention first” strategy (6) but also incorporates the potential to identify poor health behaviors of children, adolescents, their teachers and school principals as the first step to intervene. The Austrian secondary school curricula (51–56) still has a huge potential untapped to draw the attention of experts in education and health because, to date, this basic, low-cost, safe and effective dual approach has not been fully taken into account and thoroughly executed (87). Moreover, this is in line with the agenda of the European Parliament in demanding and emphasizing a “shift to prevention” as the most important considering future interventions, measures and programs for better health of nations (178). However, the pervasiveness of overweight/obesity and physical inactivity is currently too prevalent in children and adolescents worldwide and in Austria (6, 19, 26, 29, 30).

Although school-based interventions targeting PA and/or nutrition still result in inconsistent findings for decreasing overweight/obesity (44, 46, 145, 148, 174), the school setting provides an excellent existing opportunity to develop health behavior in addition to the family setting. A multi-level (from individual/family to governmental policy-making), multi-dimensional (various areas, e.g., lifestyle, behavior; and settings, e.g., school, community; national health care system and services, statutory health insurance) approach to health is key for overcoming the challenges of individual health as well as public health (6, 133, 134). Since gaps remain unbridged regarding current scientific evidence and practical settings, a more de-musicalized, integrated, and holistic lifestyle-focused approach seems most promising to add a major contribution in stopping, controlling and, at best, reversing two of today‘s major childhood health problems. This dual approach of PA, sports & exercise constantly tied to healthy diets, at the least, with its potential to prevent NCDs and promote sustainable health, remains largely untapped as a minimum recommendation. Findings from the present study will help establish health-oriented action competence and sustainable health-related action readiness regarding improvements to the current and long-term health status of children and adolescents.

*From Science 2 School* will provide data from the secondary school context as one of the key settings to shape future public health by creating permanent bondage between PA, sports & exercise with distinct diet schemes in secondary school pupils and made sustainable through competence-orientated health literacy. Through the dual-linkage of PA, sports & exercise with optimal diet, healthy lifestyles may be largely achieved with the future perspective of long-term NCD prevention and avoiding premature death (4, 5, 66, 94) by tapping the full potential of the state mandate of the Austrian curricula (51–56) concerning health promotion and literacy (47, 139, 140).

Based on the sound evidence considering health promotion by vegetarian and vegan diets, the American Medical Association (179) recommends that all medical facilities and hospitals offer plant-based (especially vegan) meals to improve the health of their patients, staff and visitors, and to eliminate animal products from the menu. In Portugal, it is therefore mandatory by law (government resolution: 3.3.2017) to offer at least one vegan dish in public canteens, i.e., in all schools, universities, colleges, hospitals, and other public institutions like prisons (180). According to the increasingly convincing body of evidence (76), vegetarian-vegan meals and menus should therefore be reflected in the public catering of educational settings (buffets, canteens, refectories, vending machines) on the one hand and the curricula, teaching and learning of schools and colleges/universities on the other hand (18, 87, 98).

Results from this project may be of help for policy- and decision-makers in the secondary educational context (governmental and federal authorities, school management, principals, teachers, families) to reconsider current health-related school offerings in order to rebuild and/or upgrade programs, measures, opportunities, and environments in everyday school scenarios (buffet, catering, vending machines, events, etc.).

#### Legal action for transfer and implementation in the educational context

Legislation and case law are also dealing with the new field of tension in the school context of vegetarian/vegan nutrition (expected 2–5 pupils eat a vegetarian or vegan diet in each group/class of 25 children). In the educational context of Austrian and German public catering, individual preferences and dislikes must be reflected in the food offered due to social and psychological significances of food (98, 181).

However, health promotion and education, preferably by the promising dual approach to sustainable health with its complex subject areas, is one of the top learning goals of didactic interventions and thus overarching educational principle by the state mandate of Austrian secondary school curricula. Health promotion and education is therefore relevant to all compulsory subject. Despite this, it is seen primarily as a special task of school sports, particularly with the compulsory subject of “Physical Education” having the “leading role” (51–56, 98).

In light of the state's educational mandate for teaching, learning and research, the dual health approach as a permanent link between the two lifestyle factors and main pillars of sustainable health: (1) regular exercise & sport and (2) healthy (plant-based) nutrition can, on the one hand, be regarded as a minimum recommendation, and on the other hand, as a “best practice” measure for health promotion, as it offers a particularly promising solution and maximum success through cumulative health effects. This interdisciplinary educational mandate must be continued seamlessly in the tertiary education setting by means of teaching/training and research mandates, especially anchored in the curricula of pedagogy studies/teachers training, health professions and life sciences, in order to enable future educators, teachers/lecturers, experts and multipliers accordingly for health education in teaching, learning and research (18, 87, 98).

### Limitations and strengths of the study

Not all pupils, teachers, and principals were within reach of the chosen recruitment method. In addition, the present study has the following limitations: (1) the cross-sectional design of the study; (2) possible over-reporting (e.g., longer duration of PA) or under-reporting (e.g., lower body weight) due to self-reporting based on socially desired statements. (3) Furthermore, part of the data collection at the time when COVID-19 related measures (inclusive lockdown) were put into action in March 2020 and affected the public, schools and universities in a later leg of the study. Although the COVID-19 situation was highly relevant to the school setting and this study, it was not possible to take into account this unpredictable situation in the online survey without any risk or consequences potentially affecting the data gathered (e.g., loss of data due to stopping and re-starting the online-survey, conflicting data sets of pre vs. during vs. post-COVID-19 situation, getting biased data, etc.). (4) Additionally, the validation process for the questionnaire showed that each item included in it must be considered individually. This occurrence means that every variable in the study is based on a single-item construct leading to different limitations: i.e., lower precision in representing the related attribute, low number of discrimination points (meaning larger sample sizes are required), and impossibility to assess internal consistency (182). (5) Nonetheless, recent research has demonstrated that single-item questionnaires may be considered valid (183) and can be consistently used in the social sciences as they are easier to apply (184, 185) and may significantly reduce the problems associated with lengthy surveys (186).

The present study, however, has several strengths, too. Since the scientific data available on the research question is limited to adults and the overall social context, and since information is lacking, in particular, (1) on children and adolescents and (2) in the school context, this school study is the first to examine this two-dimensional approach to health behavior, i.e., current nutritional behavior combined with PA behavior, Austria-nationwide based on a large sample. The study aims to provide a large data set resulting in robust information for the comparison of the respective subgroups (pupils vs. teachers/principals; omnivorous, vegetarian, vegan; physically active vs. inactive; secondary level I vs. II; federal states), which includes epidemiological as well as demographic-biometric aspects. A large number of participants helps to recognize clear differences between the subgroups and identify specific factors (e.g., age, school type) and aspects (health behavior assessment).

With the current sample size of 8,845 pupils and 1,350 adults (before data clearance), this effort for a representative data set seems reasonable to be achieved for the sample of the pupils at least (see aforementioned: Power Analysis and Representatively). The representativeness can be determined regarding various factors, such as demographic and biometric data, data on the school context, current PA and nutrition behavior, etc.

## Conclusions

*From Science 2 School: Sustainably healthy—active & veggy* is the first study to (i) assess the prevalence of current dietary trends (mixed, vegetarian, vegan) with (ii) a special focus on the linkage of the lifestyle factors PA, sports & exercise in the school context—especially for the peer groups of children and adolescents aged 10–19 years. Additionally, *From Science 2 School* has taken a large sample into account. The survey of the health behavior of Austrian pupils, teachers and principals in secondary schools I and II, allows for further examination of the topic with particularly meaningful outcomes regarding epidemiological factors and other health behaviors by allocating results to the school environment. This school study can make a significant contribution to help in reflecting on and rethinking conventional health approaches with a more functional and highly impactful dual approach. The duality of regular PA, sports & exercise consistently combined with healthy diets holds a promising “prevention first” perspective considering a meaningful contribution to public health in terms of a minimal recommendation for sustainable and lifelong health. In addition to this study, future interventions should be designed to provide a deeper understanding of the integrated role of diet type on children's health behaviors, particularly the interactions between diet type and/or physical activity level with other lifestyle factors.

## Data availability statement

The datasets presented in this article are not readily available because due to Austrian data security law, and additionally to the requirements of all the nine Austrian federal educational authorities considering data on pupils, it is not applicable. Requests to access the datasets should be directed to katharina.wirnitzer@ph-tirol.ac.at.

## Ethics statement

The study was conducted according to the medical professional codex, the Helsinki Declaration as of 1996, Data Security Laws and good clinical practice guidelines. Study participation was voluntary and could be cancelled at any time without provision of reasons or negative consequences.

Information on the survey was available on the official website and set up for this purpose. The participants were briefed that participation was voluntary, the survey was anonymous, confidential and processed exclusively for scientific purposes. Prior to the study, participants received written information about the content and procedure of the planned study and gave their written consent online to participate in the survey, prior to publication of the study results. If the respondent discontinued participation in the study, the respective data records were deleted.

The present Austrian-wide study is supported by the Federal Ministry of Education, Science and Research, Department 1/7 – School and University Sports. The study protocol was approved by the Rectorate of the University College of Teacher Education Tyrol ethics board (PHT-HSa-17-Z1.8-5n_2427; September 21, 2019) in accordance with the ethics board of the nine Austrian Federal Education Authorities, which was required in order to contact the schools. The final mandatory step to get permission to start the study and conduct the survey in the school setting was the approval by the respective principals at 2,688 schools of secondary levels I and II all over Austria.

## Author contributions

KW: conceptualization and study design. KW, GG, CD, and AC: methodology and formal analysis. KW, MM, and DT: original draft preparation. KW, DT, MM, CD, GR, and WK: writing, critical review and edits. GW: tech support. All authors read and agreed to the published version of the manuscript.

## Funding

This Austria nationwide school study is funded by the TWF (Tiroler Wissenschaftsförderung; reference number: UNI-0404/2413). The TWF was and is still not involved, and thus, there is no impact from the funding agency on study design, conduction, data collection, data analysis, presentation, and publication of the findings.

## Conflict of interest

The authors declare that the research was conducted in the absence of any commercial or financial relationships that could be construed as a potential conflict of interest.

## Publisher's note

All claims expressed in this article are solely those of the authors and do not necessarily represent those of their affiliated organizations, or those of the publisher, the editors and the reviewers. Any product that may be evaluated in this article, or claim that may be made by its manufacturer, is not guaranteed or endorsed by the publisher.

## Supplementary material

Questionnaires for pupils and teachers/principals of Austrian secondary schools are attached: Appendix A.1. Questionnaire version for pupils of Austrian secondary school levels I and II (in German). Appendix A.2. Questionnaire version for teachers and principals of Austrian secondary school levels I and II (in German).

## References

[1] American College of Lifestyle Medicine. JAMA Physician Competencies for Prescribing Lifestyle Medicine; Definition. Evidence Overwhelmingly Supports Efficacy of Lifestyle Medicine. Lifestyle Medicine Research (2020). Available online at: https://www.lifestylemedicine.org/ACLM/Lifestyle_Medicine/Research/ACLM/About/What_is_Lifestyle_Medicine_/LM_Research.aspx?hkey=dde46b29-faec-459b-b719-6432ad5172d0 (accessed December 03, 2020).

[2] NCD Risk Factor Collaboration (NCD-RisC). Worldwide trends in body-mass index, underweight, overweight, and obesity from 1975 to 2016: a pooled analysis of 2416 population-based measurement studies in 128.9 million children, adolescents, and adults. Lancet. (2017) 390:2627–42. 10.1016/S0140-6736(17)32129-3 29029897 PMC5735219

[3] The World Health Organization. Global Action Plan for the Prevention and Control of Non-Communicable Diseases: 2013–2020. (2013). Available online at: https://apps.who.int/iris/bitstream/handle/10665/94384/9789241506236_eng.pdf;jsessionid=72665324C3A58DEFD129427F62384ED4?sequence=1 (accessed December 03, 2020).

[4] The World Health Organization. Non-communicable diseases. (2021). Available online at: https://www.who.int/news-room/fact-sheets/detail/noncommunicable-diseases (accessed June 29, 2020).

[5] World Health Organization. Preventing Non-Communicable Diseases. (2021). Available online at: https://www.who.int/activities/preventing-noncommunicable-diseases (accessed January 19, 2020).

[6] Wirnitzer K.C Drenowatz C. An integrative approach in addressing today's global health crisis. In:Wirnitzer K Drenowatz C Kirschner W Tanous D Rosemann T, editor. International Research & Knowledge Exchange for Addressing Today's Global Health Paradox, 1st Edn. Event Abstracts. Editorial Main (2020). p. 14–22.

[7] Barker DJ Osmond C Kajantie E Eriksson JG. Growth and chronic disease: findings in the Helsinki Birth Cohort. Ann Hum Biol. (2009) 36:445–58. 10.1080/03014460902980295 19562567

[8] Jiménez-Pavón D Konstabel K Bergman P Ahrens W Pohlabeln H Hadjigeorgiou C. Physical activity and clustered cardiovascular disease risk factors in young children: a cross-sectional study (the IDEFICS study). BMC Med. (2013) 11:172. 10.1186/1741-7015-11-172 23899208 PMC3728104

[9] Mikkelsen B Williams J Rakovac I Wickramasinghe K Hennis A Shin HR. Life course approach to prevention and control of non-communicable diseases. BMJ. (2019) 364:l257. 10.1136/bmj.l257 30692103 PMC6349133

[10] Motevalli M Drenowatz C Tanous DR Khan NA Wirnitzer K. Management of childhood obesity—time to shift from generalized to personalized intervention strategies. Nutrients. (2021) 13:1200. 10.3390/nu13041200 33917383 PMC8067342

[11] World Health Organization. Report of the Commission on Ending Childhood Obesity. Implementation Plan: Executive Summary. (2017). Available online at: https://apps.who.int/iris/bitstream/handle/10665/259349/WHO-NMH-PND-ECHO-17.1-eng.pdf (accessed July 23, 2021).

[12] Belanger M Sabiston CM Barnett TA O'Loughlin E Ward S Contreras G. Number of years of participation in some, but not all, types of physical activity during adolescence predicts level of physical activity in adulthood: results from a 13-year study. Int J Behav Nutr Phys Act. (2015) 12:76. 10.1186/s12966-015-0237-x 26058349 PMC4464637

[13] Gries KJ Raue U Perkins RK Lavin KM Overstreet BS D'Acquisto LJ. Cardiovascular and skeletal muscle health with lifelong exercise. J Appl Physiol. (2018) 125:1636–45. 10.1152/japplphysiol.00174.2018 30161005 PMC6295480

[14] Myers J McAuley P Lavie CJ Despres JP Arena R Kokkinos P. Physical activity and cardiorespiratory fitness as major markers of cardiovascular risk: their independent and interwoven importance to health status. Prog Cardiovasc Dis. (2015) 57:306–14. 10.1016/j.pcad.2014.09.011 25269064

[15] Physicians Committee for Responsible Medicine. Four Ways Vegan Diets Can Benefit Kids. (2016). Available online at: https://www.pcrm.org/news/blog (accessed July 21, 2018).

[16] Physicians Committee for Responsible Medicine. Vegetarian Diets: Advantages for Children. (2018). Available online at: http://www.pcrm.org/sites/default/files/pdfs/health/info_advchild.pdf (accessed May 14, 2018).

[17] Telama R. Tracking of physical activity from childhood to adulthood: a review. Obes Facts. (2009) 2:187–95. 10.1159/000222244 20054224 PMC6516203

[18] Wirnitzer K. Vegan diet in sports and exercise – Health benefits and advantages to athletes and physically active people: a narrative review. Int J Sports Exerc Med. (2020) 6:165. 10.23937/2469-5718/1510165

[19] Wirnitzer KC Drenowatz C. Improving Child & Adolescent Health for better Public Health – Fiction or within the scope of possibility? The perspective of a lifestyle-centered approach for Addressing Today's Global Health Paradox. In:Wirnitzer K Drenowatz C Kirschner W Tanous D Rosemann T, editor. International Research & Knowledge Exchange for Addressing Today's Global Health Paradox, 1st Edn. Editorial Meeting 2 (2020). p. 45–47.

[20] Yamada Y. Dietary Intake and Physical Activity for Human Health (Message from the Guest Editor). (2020). Available online at: https://www.mdpi.com/journal/nutrients/special_issues/Dietary_Physical_Health (accessed on July 27, 2021).

[21] Euractiv Special Report. Physical Inactivity: A Ticking Time Bomb in the EU. (2015). Available online at: http://en.euractiv.eu/wp-content/uploads/sites/2/special-report/euractiv_special_report_-_physical_inactivity_a_ticking_timebomb_in_the_eu.pdf (accessed September 12, 2017).

[22] GBD 2017 Diet Collaborators. Health effects of dietary risks in 195 countries, 1990-2017: a systematic analysis for the Global Burden of Disease Study 2017. Lancet. (2019) 393:1958–72. 10.1016/S0140-6736(19)30041-8 30954305 PMC6899507

[23] Wirnitzer K Drenowatz C Kirschner W Tanous D Rosemann T. International research & knowledge exchange for addressing today's global health paradox. Front Public Health. (2020) 10.32140456

[24] Euractiv Special Report. EU at ‘Tipping Point' in Reversing Current Nutritional Trends, Report Highlights. (2021). Available online at: https://www.euractiv.com/section/agriculture-food/news/eu-at-tipping-point-in-reversing-current-nutritional-trends-report-highlights/ (accessed July 27, 2021).

[25] Euractiv Special Report. Obesity Must Not Become a New Normal, MEPs Warn. (2021). Available online at: https://www.euractiv.com/section/health-consumers/news/obesity-must-not-become-a-new-normal-meps-warn/ (accessed July 27, 2021).

[26] Felder-Puig R Teutsch F Ramelow D Maier G. Health and Health Behavior of Austrian Schoolchildren. Results of the WHO-HBSC-Survey (2018) (Gesundheit und Gesundheitsverhalten von österreichischen Schund Sch. Ergebnisse des WHO-HBSC-Survey 2018). Bundesministerium fArbeit, Soziales, Gesundheit und Konsumentenschutz (eds) Wien (2019). Available online at: https://www.sozialministerium.at/cms/site/attachments/8/8/2/CH4154/CMS1562043067885/2018_hbsc-bericht_mit_alternativtexten_final.pdf (accessed January 17, 2020).

[27] Guthold R Stevens GA Riley LM Bull FC. Worldwide trends in insufficient physical activity from 2001 to 2016: a pooled analysis of 358 population-based surveys with 1.9 million participants. Lancet Glob Health. (2018) 6:e1077–86. 10.1016/S2214-109X(18)30357-7 30193830

[28] Guthold R Stevens GA Riley LM Bull FC. Global trends in insufficient physical activity among adolescents: a pooled analysis of 298 population-based surveys with 1.6 million participants. Lancet Child Adolesc Health. (2020) 4:23–35. 10.1016/S2352-4642(19)30323-2 31761562 PMC6919336

[29] World Health Organization – Regional Office for Europe (2020). Available online at: https://www.euro.who.int/en/publications/abstracts/spotlight-on-adolescent-health-and-well-being.-findings-from-the-20172018-health-behaviour-in-school-aged-children-hbsc-survey-in-europe-and-canada.-international-report.-volume-1.-key-findings (accessed December 03, 2020).

[30] World Health Organization. Regional Office for Europe. (2020). Available online at: https://www.euro.who.int/en/health-topics/Life-stages/child-and-adolescent-health/health-behaviour-in-school-aged-children-hbsc/publications/2020/spotlight-on-adolescent-health-and-well-being.-findings-from-the-20172018-health-behaviour-in-school-aged-children-hbsc-survey-in-europe-and-canada.-international-report.-volume-2.-key-data (accessed December 03, 2020).

[31] Lee IM Shiroma EJ Lobelo F Puska P Blair SN Katzmarzyk PT. Effect of physical inactivity on major non-communicable diseases worldwide: an analysis of burden of disease and life expectancy. Lancet. (2012) 380:219–29. 10.1016/S0140-6736(12)61031-9 22818936 PMC3645500

[32] The World Health Organization. Physical Activity Strategy for the WHO European Region. Regional committee for Europe 65th session (2015). Available online at: https://www.euro.who.int/__data/assets/pdf_file/0010/282961/65wd09e_PhysicalActivityStrategy_150474.pdf (accessed on March 24, 2020).

[33] Wirnitzer K Boldt P Lechleitner C Wirnitzer G Leitzmann C Rosemann T. Health status of female and male vegetarian and vegan endurance runners compared to omnivores – results from the NURMI Study (Step 2). Nutrients. (2018) 11:29. 10.3390/nu11010029 30583521 PMC6356807

[34] Global BMI Mortality Collaboration Di Angelantonio E Bhupathiraju S Wormser Wormser D Gao P. Body-mass index and all-cause mortality: individual-participant-data meta-analysis of 239 prospective studies in four continents. Lancet. (2016) 388:776–86. 10.1016/S0140-6736(16)30175-1 27423262 PMC4995441

[35] World Health Organization. How Healthy Are Children's Eating Habits? – WHO/Europe Surveillance Results. (2021). Available at: Available online at: https://www.euro.who.int/en/health-topics/noncommunicable-diseases/obesity/news/news/2021/3/how-healthy-are-childrens-eating-habits-whoeurope-surveillance-results (accessed February 04, 2021).

[36] United Nations Educational, Scientific and Cultural Organization. Education for Sustainable Development Goals. Learning Objectives (2017). Available online at: https://www.unesco.de/sites/default/files/2018-08/unesco_education_for_sustainable_development_goals.pdf (accessed December 03, 2020).

[37] United Nations. Sustainable Development Goals. Transforming our World. The 2030 agenda for sustainable development (2015). Available online at: https://sustainabledevelopment.un.org/post2015/transformingourworld; Available online at: https://www.undp.org/content/dam/undp/library/corporate/brochure/SDGs_Booklet_Web_En.pdf (accessed December 03, 2020).

[38] World Health Organization. World Health Statistics. (2016). Monitoring health for the SDGs. (2016). Available online at: http://www.who.int/gho/publications/world_health_statistics/2016/en/ (accessed September 12, 2017).

[39] Independent Accountability Panel. The Health of Women, Children and Adolescents Is at the Heart of Transforming Our World: Empowering Accountability. Final reflections report (2021). Available online at: https://iapewec.org/reports/iap-2021-final-report/ (accessed July 23, 2021).

[40] Brown T Summerbell C. Systematic review of school-based interventions that focus on changing dietary intake and physical activity levels to prevent childhood obesity: an update to the obesity guidance produced by the National Institute for Health and Clinical Excellence. Obes Rev. (2009) 10:110–41. 10.1111/j.1467-789X.2008.00515.x 18673306

[41] De Bourdeaudhuij I Van Cauwenberghe E Spittaels H Oppert JM Rostami C Brug J. School-based interventions promoting both physical activity and healthy eating in Europe: a systematic review within the HOPE project. Obes Rev. (2011) 12:205–16. 10.1111/j.1467-789X.2009.00711.x 20122137

[42] Evans CE Albar SA Vargas-Garcia EJ Xu F. School-based interventions to reduce obesity risk in children in high- and middle-income countries. Adv Food Nutr Res. (2015) 76:29–77. 10.1016/bs.afnr.2015.07.003 26602571

[43] García-Hermoso A Alonso-Martínez AM Ramírez-Vélez R Pérez-Sousa MÁ Ramírez-Campillo R Izquierdo M. Association of physical education with improvement of health-related physical fitness outcomes and fundamental motor skills among youths: a systematic review and meta-analysis. JAMA Pediatr. (2020) 174:e200223. 10.1001/jamapediatrics.2020.0223 32250414 PMC7136862

[44] The Institute of Development Studies. The Impact of School Health Programmes. (2017). Available online at: https://opendocs.ids.ac.uk/opendocs/handle/20.500.12413/13185 (accessed August 28, 2021).

[45] Parrish AM Okely AD Stanley RM Ridgers ND. The effect of school recess interventions on physical activity: a systematic review. Sports Med. (2013) 43:287–99. 10.1007/s40279-013-0024-2 23512170

[46] Yuksel HS Sahin FN Maksimovic N Drid P Bianco A. School-based intervention programs for preventing obesity and promoting physical activity and fitness: a systematic review. Int J Environ Res Public Health. (2020) 17:347. 10.3390/ijerph17010347 31947891 PMC6981629

[47] The World Health Organization. Health promoting schools. An effective approach to early action on noncommunicable disease risk factors. (2017). Available online at: https://www.who.int/publications/i/item/health-promoting-school-an-effective-approach-for-early-action-on-ncd-risk-factors (accessed April 29, 2021).

[48] Costello A Peterson S Rasanathan K. Strategic review of child health where's the leadership? Future commitments of UNICEF and WHO for global child health. BMJ. (2018) 362:k3543. 10.1136/bmj.k3543 30061193 PMC6064977

[49] National Research Council (US); Institute of Medicine (US). Children's Health, The Nation's Wealth: Assessing and Improving Child Health. Part 2 - Children's Health: A New Conceptual Framework Committee on Evaluation of Children‘S Health, Board on Children, Youth, and Families, Division of Behavioral and Social Sciences and Education. Washington, DC: National Academies Press (2004).

[50] BMUK – *Grundsatzerlass Gesundheitserziehung*. (1997). Available online at: https://schularzt.at/fileadmin/user_upload/Grundsatzerlass_Gesundheitserziehung_1997.pdf (accessed April 14, 2021).

[51] Lehrplan AHS. Unterstufe (Sekundarstufe I). (2021). Available online at: https://www.bmbwf.gv.at/Themen/schule/schulpraxis/lp/lp_ahs.html (accessed September 03, 2021).

[52] Lehrplan AHS Unterstufe (Sekundarstufe I) (2021). Available online at: https://www.ris.bka.gv.at/GeltendeFassung.wxe?Abfrage=Bundesnormen&Gesetzesnummer=10008568 (accessed September 03, 2021).

[53] Lehrplan AHS Oberstufe (Sekundarstufe II) (2018). Available online at: https://www.bmbwf.gv.at/Themen/schule/schulpraxis/lp/lp_ahs.html; Available online at: https://www.ris.bka.gv.at/GeltendeFassung.wxe?Abfrage=Bundesnormen&Gesetzesnummer=10008568&FassungVom=2018-09-01 (accessed September 03, 2021).

[54] Lehrplan AHS Oberstufe (Sekundarstufe II). (2021). Available online at: https://www.ris.bka.gv.at/GeltendeFassung.wxe?Abfrage=Bundesnormen&Gesetzesnummer=10008568 (accessed September 03, 2021).

[55] Lehrplan der Neuen Mittelschule (2018). Available online at: https://www.ris.bka.gv.at/Dokumente/Bundesnormen/NOR40199276/NOR40199276.pdf (accessed June 01, 2020).

[56] Lehrplan der Neuen Mittelschule. Sechster Teil (2018). Available online at: https://www.ris.bka.gv.at/Dokumente/Bundesnormen/NOR40199276/NOR40199276.pdf (accessed June 01, 2020).

[57] American College of Sports Medicine. ACSM Exercise Guidelines | 3 Essential Resources. (2020). Available online at: https://www.acsm.org/blog-detail/acsm-certified-blog/2020/09/03/acsm-exercise-guidelines-resources (accessed July 27, 2021).

[58] Jeukendrup A. Would you want a drug that does all of this? Free of charge safe for children? Now available everywhere! It is called physical activity. (2018). Available online at: https://twitter.com/jeukendrup/status/849548949216268288 (accessed December 03, 2020).

[59] Khan KM Thompson AM Blair SN Sallis JF Powell KE Bull FC. Sport and exercise as contributors to the health of nations. Lancet. (2012) 380:59–64. 10.1016/S0140-6736(12)60865-4 22770457

[60] Koehler K Drenowatz C. Integrated role of nutrition and physical activity for lifelong health. Nutrients. (2019) 11:1437. 10.3390/nu11071437 31247924 PMC6682932

[61] American College of Sports Medicine. Staying physically active during the Covid-19 pandemic. (2020). Available online at: https://www.acsm.org/read-research/newsroom/news-releases/news-detail/2020/03/16/staying-physically-active-during-covid-19-pandemic (accessed December 03, 2020).

[62] Guan H Okely AD Aguilar-Farias N Del Pozo Cruz B Draper CE El Hamdouchi A. Promoting healthy movement behaviours among children during the COVID-19 pandemic. Lancet Child Adolesc Health. (2020) 4:416–8. 10.1016/S2352-4642(20)30131-0 32458805 PMC7190292

[63] Jakobsson J Malm C Furberg M Ekelund U Svensson M. Physical activity during the coronavirus (COVID-19) pandemic: prevention of a decline in metabolic and immunological functions. Front Sports Act Living. (2020) 2:57. 10.3389/fspor.2020.00057 33345048 PMC7739799

[64] Mattioli AV Sciomer S Cocchi C Maffei S Gallina S. Quarantine during COVID-19 outbreak: changes in diet and physical activity increase the risk of cardiovascular disease. Nutr Metab Cardiovasc Dis. (2020) 30:1409–17. 10.1016/j.numecd.2020.05.020 32571612 PMC7260516

[65] Sallis R Young DR Tartof SY Sallis JF Sall J Li Q. Physical inactivity is associated with a higher risk for severe COVID-19 outcomes: a study in 48440 adult patients. Br J Sports Med. (2021) 55:1099–105. 10.1136/bjsports-2021-104080 33849909

[66] World Health Organization. Stay Physically Active During Self-Quarantine. (2021). Available online at: http://www.euro.who.int/en/health-topics/health-emergencies/coronavirus-covid-19/novel-coronavirus-2019-ncov-technical-guidance-OLD/stay-physically-active-during-self-quarantine?fbclid=IwAR3QGWe_fktH477OnO9dLfD8Tt5oyGg7nHCOhCPZ0Knv9aIw_LCDMO3n_qw#article (accessed July 22, 2021).

[67] Balan E Decottignies A Deldicque L. Physical activity and nutrition: two promising strategies for telomere maintenance? Nutrients. (2018) 10:1942. 10.3390/nu10121942 30544511 PMC6316700

[68] European Commission – Nutrition and physical activity (2021). Available online: Available online at: https://ec.europa.eu/health/nutrition_physical_activity/overview_en (accessed July 23, 2021).

[69] EUR-Lex (EU) – Council conclusions on nutrition and physical activity (2014). Available online at: https://eur-lex.europa.eu/legal-content/EN/ALL/?uri=OJ:C:2014:213:FULL (accessed August 28, 2021).

[70] Godoy-Cumillaf A Fuentes-Merino P Díaz-González A Jiménez-Díaz J Martínez-Vizcaíno V Álvarez-Bueno C. The effects of physical activity and diet interventions on body mass index in Latin American children and adolescents: a systematic review and meta-analysis. Nutrients. (2020) 12:1378. 10.3390/nu12051378 32408483 PMC7284900

[71] Kaiser Permanente. Healthy eating active living (heal) program health promotion, and synopses. (2016). Available online: http://gken.org/Synopses/HP_10003.pdf (accessed September 12, 2017).

[72] Komulainen P Tuomilehto J Savonen K Männikkö R. Exercise, diet, and cognition in a 4-year randomized controlled trial: Dose-Responses to Exercise Training (DR's EXTRA). Am J Clin Nutr. (2021) 113:1428–39. 10.1093/ajcn/nqab018 33742194 PMC8244125

[73] Sweet SN Fortier MS. Improving physical activity and dietary behaviours with single or multiple health behaviour interventions? A synthesis of meta-analyses and reviews. Int J Environ Res Public Health. (2010) 7:1720–43. 10.3390/ijerph7041720 20617056 PMC2872344

[74] Tuso PJ Ismail MH Ha BP Bartolotto C. Nutritional update for physicians: plant-based diets. Perm J. (2013) 17:61–6. 10.7812/TPP/12-085 23704846 PMC3662288

[75] Tuso P. Physician update: total health. Perm J. (2014) 18:58–63. 10.7812/TPP/13-120 24694316 PMC4022559

[76] Leitzmann C Keller M. Vegetarische und vegane Ernährung. 4, vollständig überarbeitete und erweiterte Auflage. UTB, Stuttgart, Germany (2020).

[77] Appleby PN Key TJ. The long-term health of vegetarians and vegans. Proc Nutr Soc. (2016) 75:287–93. 10.1017/S0029665115004334 26707634

[78] Greger M. How Not to Die: Discover the Foods Scientifically Proven to Prevent and Reverse Disease. Croydon: CPI Group (UK) Ltd (2017).

[79] Li D. Effect of the vegetarian diet on non-communicable diseases. J Sci Food Agric. (2014) 94:169–73. 10.1002/jsfa.6362 23965907

[80] Physicians Committee for Responsible Medicine – Frequently Asked Questions About Nutrition (2018). Available online: www.pcrm.org/health/diets/vegdiets/frequently-asked-questions-about-nutrition#RecommendVegDiet (accessed June 14, 2018).

[81] Rizzo NS Sabaté J Jaceldo-Siegl K Fraser G. Vegetarian dietary patterns are associated with a lower risk of metabolic syndrome: the Adventist Health Study 2. Diabetes Care. (2011) 34:1225–7. 10.2337/dc10-1221 21411506 PMC3114510

[82] Tonstad S Stewart K Oda K Batech M Herring RP Fraser GE. Vegetarian diets and incidence of diabetes in the Adventist Health Study-2. Nutr Metab Cardiovasc Dis. (2013) 23:292–9. 10.1016/j.numecd.2011.07.004 21983060 PMC3638849

[83] Physicians Committee for Responsible Medicine – Plant-Based Diets (2020). Available online at: https://www.pcrm.org/good-nutrition/plant-based-diets#RecommendVegDiet (accessed August 28, 2022).

[84] Barnard N. Your Body in Balance. The New Science of Food, Hormones, and Health. New York, NY: Grand Central Publishing (2020).

[85] Kadoch MA. The power of nutrition as medicine. Prev Med. (2012) 55:80. 10.1016/j.ypmed.2012.04.013 22561031

[86] William A. Medical Food. Warum Obst und Gemüse als Heilmittel potenter sind als jedes Medikament. Arkana, München (2017).

[87] Wirnitzer K. Therapeutic, Probiotic, and Unconventional Foods. In:Wirnitzer KC, editor. Vegan Nutrition: Latest Boom in Health and Exercise London: Elsevier (2018). 10.1016/B978-0-12-814625-5.00020-0

[88] Popper P. Food Over Medicine. Dallas, TX: Benbella Books (2013).

[89] de Faria Coelho-Ravagnani C Corgosinho FC Sanches F FZ Prado CMM Laviano A Mota JF. Dietary recommendations during the COVID-19 pandemic. Nutr Rev. (2021) 79:382–93. 10.1093/nutrit/nuaa067 32653930 PMC7454801

[90] The Centre for Evidence-Based Medicine (2020). Available online at: https://www.cebm.net/covid-19/what-explains-the-high-rate-of-SARS-CoV-2-transmission-in-meat-and-poultry-facilities-2/ (accessed July 22, 2021).

[91] Durand-Moreau Q Mackenzie G Adisesh A Straube S Chan XHS Zelyas N. Twitter analytics to inform provisional guidance for COVID-19 challenges in the meatpacking industry. Ann Work Expo Health. (2021) 65:373–6. 10.1093/annweh/wxaa123 33492381 PMC7929462

[92] Greger M. Primary pandemic prevention. Am J Lifestyle Med. (2021) 15:498–505. 10.1177/15598276211008134 34646097 PMC8504329

[93] Kim H Rebholz CM Hegde S LaFiura C Raghavan M Lloyd JF. Plant-based diets, pescatarian diets and COVID-19 severity: a population-based case–control study in six countries. BMJ Nutr Prev Health. (2021) 4:257–66. 10.1136/bmjnph-2021-000272 34308134 PMC8219480

[94] World Health Organization. Food nutrition tips during self-quarantine (2021). Available online at: http://www.euro.who.int/en/health-topics/health-emergencies/coronavirus-covid-19/novel-coronavirus-2019-ncov-technical-guidance-OLD/food-and-nutrition-tips-during-self-quarantine?fbclid=IwAR0IxmHZqgX-uwgq0cNTsDM3BdHUogV8EcFbqiY3olALGzBP_hbzW6AwYnA (accessed July 22, 2021).

[95] Heinrich-Böll-Stiftung – Meat Atlas (2014). Available online at: https://www.foeeurope.org/sites/default/files/publications/foee_hbf_meatatlas_jan2014.pdf (accessed April 30, 2021).

[96] STATISTA (2020). Available online at: https://de.statista.com/statistik/daten/studie/709815/umfrage/anzahl-der-vegetarier-veganer-und-flexitarier-in-oesterreich/ (accessed July 30, 2021).

[97] Heinrich-Böll-Stiftung – Meat Atlas (2021). Available online at: https://www.boell.de/sites/default/files/2021-01/Fleischatlas2021_0.pdf?dimension1=ds_fleischatlas_2021 (accessed July 30, 2021).

[98] Wirnitzer K. Nachhaltig gesund – Vegane Ernährung in Bewegung und Sport. Übersichtsartikel. Fachzeitschrift Bewegung & Sport, Heft 3, Schwerpunkt Ernährung & Sport: 27–34. (2021). Available online at: https://www.science2.school/wp-content/uploads/2021/11/WirnitzerKC_Vegan-Sport_final-online_full-version_FachZS-BUS_4Nov2021.pdf (accessed August 28, 2022).

[99] Parker J. The Year of the Vegan. Where Millennials Lead, Business Governments Will Follow. The World in 2019 (2018). Available online at: https://worldin2019.economist.com/theyearofthevegan?utm_source=412&utm_medium=COM (accessed March 10, 2020).

[100] Pellman-Rowland M. Millenials Are Driving the Worldwide Shirt Away From Meat. (2018). Available online at: https://www.forbes.com/sites/michaelpellmanrowland/2018/03/23/millennials-move-away-from-meat/#618a21aa4a49 (accessed March 10, 2020).

[101] Chiorando M. 30% of British Shoppers Aged 18-24 Are Vegan or Considering It. Plant Based News (2018). Available online at: https://www.plantbasednews.org/culture/30-british-shoppers-18-24-vegan-considering (accessed June 13, 2022).

[102] Chiorando M. Young People In UK Are ‘Ditching Meat In Record Numbers'. Plant Based News (2019) Available online at: https://www.plantbasednews.org/news/young-people-uk-ditching-meat-record-numbers?utm_content=bufferfe38a&utm_medium=social&utm_source=facebook.com&utm_campaign=buffer (accessed June 13, 2022).

[103] Chiorando M. 44% Of Generation Z Say Being Vegan Is 'Cooler Than Smoking'. Young people are driving the move towards meat-free dining. Plant Based News. (2018). Available online at: https://www.plantbasednews.org/post/44-of-generation-z-vegan-cooler-smoking (accessed June 13, 2022).

[104] Chiorando M. 1 In 12 Parents 'Raising Their Children Vegan' Says New Poll. Health was the main reason for ditching animals products (2018). Available online at: https://www.plantbasednews.org/post/new-study-says-1-in-12-parents-are-raising-their-children-vegan (accessed March 10, 2020).

[105] ker S Garcia D Valin H van Ruijven B. Using social media audience data to analyse the drivers of low-carbon diets. Environ Res Lett. (2021) 16:074001. 10.1088/1748-9326/abf770

[106] Fridays for Future (2021). Available online at: Available online at: https://fridaysforfuture.at/allianzen (accessed July 22, 2021).

[107] World Health Organization. Body Mass Index – BMI. (2021). Available online at: https://www.euro.who.int/en/health-topics/disease-prevention/nutrition/a-healthy-lifestyle/body-mass-index-bmi (accessed July 02, 2020).

[108] World Health Organization. Global Health Observatory (GHO) Data. Mean Body Mass Index (BMI). Situation and Trends. (2018). Available online at: http://www.who.int/gho/ncd/risk_factors/bmi_text/en/ (accessed September 12, 2018).

[109] Boeing H Bechthold A Bub A Ellinger S Haller D Kroke A et al. Deutschen Gesellschaft für Ernährung e. V. (DGE; Hrsg.). Stellungnahme. Gemüse und Obst in der Prävention ausgewählter chronischer Krankheiten. Bonn, Deutschland (2012). Available online at: https://www.dge.de/fileadmin/public/doc/ws/stellungnahme/DGE-Stellungnahme-Gemuese-Obst-2012.pdf (accessed September 03, 2020).

[110] Cole TJ Lobstein T. Extended international (IOTF) body mass index cut-offs for thinness, overweight and obesity. Pediatr Obes. (2012) 7:284–94. 10.1111/j.2047-6310.2012.00064.x 22715120

[111] Haftenberger M Heuer T Heidemann C Kube F Krems C Mensink GB. Relative validation of a food frequency questionnaire for national health and nutrition monitoring. Nutr J. (2010) 9:36. 10.1186/1475-2891-9-36 20840739 PMC2945984

[112] Hall JN Moore S Harper SB Lynch JW. Global variability in fruit and vegetable consumption. Am J Prev Med. (2009) 36:402–9.e5. 10.1016/j.amepre.2009.01.029 19362694

[113] Hofmann F Griebler R Ramelow D Unterweger K Griebler U Felder-Puig R. Gesundheit und Gesundheitsverhalten von Österreichs Lehrer/innen: Ergebnisse der Lehrer/innen-befragung 2010. LBIHPR Forschungsbericht, Wien, Austria (2012).

[114] Kromeyer-Hauschild K Wabitsch M Kunze D Geller F Geiß HC Hesse V. Perzentile für den Body-mass-Index für das Kindes- und Jugendalter unter Heranziehung verschiedener deutscher Stichproben. Monatsschrift Kinderheilkunde. (2001) 149:807–18. 10.1007/s001120170107

[115] Markland D Hardy L. The exercise motivations inventory: preliminary development and validity of a measure of individuals' reasons for participation in regular physical exercise. Pers Individ Differ. (1993) 15:289–96. 10.1016/0191-8869(93)90219-S

[116] Markland D Ingledew DK. The measurement of exercise motives: factorial validity and invariance across gender of a revised exercise motivations inventory. Br J Health Psychol. (1997) 2:361–76. 10.1111/j.2044-8287.1997.tb00549.x

[117] Neuhauser H Schienkiewitz A Schaffrath-Rosario A Dortschy R Kurth BM. Beiträge zur Gesundheitsberichterstattung des Bundes. Referenzperzentile für anthropometrische Maßzahlen und Blutdruck aus der Studie zur Gesundheit von Kindern und Jugendlichen in Deutschland (KiGGS). 2. erweiterte Auflage. Robert-Koch Institut, Berlin, Germany (2013).

[118] Schmid J Molinari V Lehnert K Sudeck G.; Conzelmann A. BMZI-HEA. Adaption des Berner Motiv- und Zielinventars im Freizeit- und Gesundheitssport für Menschen im höheren Erwachsenenalter. Zeitschrift für Gesundheitspsychologie. (2014) 22:104–17. 10.1026/0943-8149/a000119

[119] Wirnitzer K Seyfart T Leitzmann C Keller M Wirnitzer G. Prevalence in running events and running performance of endurance runners following a vegetarian or vegan diet compared to non-vegetarian endurance runners: the NURMI Study. Springerplus. (2016) 5:458. 10.1186/s40064-016-2126-4 27119062 PMC4831958

[120] Griebler R Winkler P Bengough T. Österreichischer Kinder- und Jugendgesundheitsbericht. Bundesministerium für Gesundheit, Wien, Austria (2016).

[121] Ramelow D Teutsch F Hofmann F Felder-Puig R. Ludwig Boltzmann Institut Health Promotion Research. Gesundheit und Gesundheitsverhalten von österreichischen Schülern und Schülerinnen. Ergebnisse des WHO-HBSC-Survey (2014). Bundesministerium für Gesundheit, Wien, Austria (2015).

[122] Leitzmann C Keller M. Vegetarische Ernährung. 3 aktualisierte Auflage Edn. UTB, Stuttgart (2013).

[123] Felder-Puig R Ramelow D Maier G Teutsc F. Ergebnisse der WieNGS Lehrer/innen-Befragung. Institut für Gesundheitsförderung und Prävention, Wien, Austria (2017).

[124] Dijkstra TK. On statistical inference with parameter estimates on the boundary of the parameter space. Br J Math Stat Psychol. (1992) 45:289–309. 10.1111/j.2044-8317.1992.tb00994.x

[125] Drenowatz C Greier K. Association of sports participation and diet with motor competence in Austrian middle school students. Nutrients. (2018) 10:1837. 10.3390/nu10121837 30501115 PMC6316641

[126] Ruedl G Greier N Niedermeier M Posch M Prünster V Faulhaber M. Factors associated with physical fitness among overweight and non-overweight Austrian secondary school students. Int J Environ Res Public Health. (2019) 16:4117. 10.3390/ijerph16214117 31731515 PMC6862517

[127] Anselma M Chinapaw MJM Altenburg TM. Determinants of child health behaviors in a disadvantaged area from a community perspective: a participatory needs assessment. Int J Environ Res Public Health. (2018) 15:644. 10.3390/ijerph15040644 29614732 PMC5923686

[128] Institute of Medicine (US) Committee on Assuring the Health of the Public in the 21st Century. The Future of the Public's Health in the 21st Century. Washington, DC: National Academies Press (2002). Chapter 2: Understanding Population health and Its Determinants. Available online at: https://www.ncbi.nlm.nih.gov/books/NBK221239/.

[129] Cheung P. Teachers as role models for physical activity: are preschool children more active when their teachers are active? Eur Phys Educ Rev. (2020) 26:101–10. 10.1177/1356336X19835240

[130] Wong LS Gibson AM Farooq A Reilly JJ. Interventions to increase moderate-to-vigorous physical activity in elementary school physical education lessons: systematic review. J Sch Health. (2021) 91:836–45. 10.1111/josh.13070 34431516

[131] Nieskens B Rupprecht S Erbring S. Was hält Lehrkräfte gesund? Ergebnisse der Gesundheitsforschung für Lehrkräfte und Schulen. In Handbuch Lehrergesundheit Impulse für die Entwicklung Guter Gesunder Schulen; Eine Veröffentlichung der DAK-Gesundheit und der Unfallkasse Nordrhein-Westfalen; Carl Link (Wolters Kuiwer): Köln, Germany (2012). p. 31–96.

[132] Wirnitzer KC Drenowatz C Cocca A Tanous DR Motevalli M Wirnitzer G. Health behaviors of Austrian Secondary School Teachers and Principals at a Glance: first results of the from science 2 school study focusing on sports linked to mixed, vegetarian, and vegan diets. Nutrients. (2022) 14:1065. 10.3390/nu14051065 35268041 PMC8912656

[133] World Health Organization – Regional Office for Europe (2007). Available online at: http://www.euro.who.int/__data/assets/pdf_file/0018/103824/E89384.pdf (accessed August 03, 2021).

[134] Economic and Social Research Council. The Dahlgren-Whitehead rainbow (2021). Available online at: https://esrc.ukri.org/about-us/50-years-of-esrc/50-achievements/the-dahlgren-whitehead-rainbow/ (accessed August 03, 2021).

[135] Nichols SDS Francis MP Dalrymple N. Sustainability of a curriculum-based intervention on dietary behaviors and physical activity among primary school children in Trinidad and Tobago. West Indian Med J. (2014) 63:68–77. 10.7727/wimj.2014.011 25303198 PMC4655622

[136] Tanous RT Ruedl G Kirschner W Drenowatz C Craddock J Rosemann T. School health programs of physical education and/or diet among pupils of primary and secondary school levels I and II linked to body mass index: a systematic review protocol within the project *From Science 2 School*. – under review. PLoS ONE. (2022) 18. 10.1371/journal.pone.0275012 PMC9536596 36201567

[137] Langford R Bonell CP Jones HE Pouliou T Murphy SM Waters E. The WHO Health Promoting School framework for improving the health and well-being of students and their academic achievement. Cochrane Database Syst Rev. (2014) 4:CD008958. 10.1002/14651858.CD008958.pub2 24737131 PMC11214127

[138] Clark H Coll-Seck AM Banerjee A Peterson S Dalglish SL Ameratunga S. A future for the world's children? A WHO-UNICEF-Lancet commission. Lancet. (2020) 395:605–58. 10.1016/S0140-6736(19)32540-1 32085821

[139] Okan O Bauer U Levin-Zamir D Pinheiro P Sorensen K. International Handbook of Health Literacy. Research, Practice and Policy Across the Lifespan. Bristol: Policy Press (2019) 10.51952/9781447344520

[140] Wojtowicz A. Roundtable on Health Literacy; Board on Population Health and Public Health Practice; Health and Medicine Division; National Academies of Sciences, Engineering, and Medicine. Developing Health Literacy Skills in Children and Youth: Proceedings of a Workshop. Washington DC: The National Academies Press (2020). 10.17226/25888 33026761

[141] Wirnitzer KC Drenowatz C Cocca A Tanous DR Motevalli M Wirnitzer G. Health behaviors of Austrian secondary level pupils at a Glance: first results of the from science 2 school study focusing on sports linked to mixed, vegetarian, and vegan diets. Int J Environ Res Public Health. (2021) 18:12782. 10.3390/ijerph182312782 34886508 PMC8657632

[142] Institute of Medicine. Committee on Comprehensive School Health Programs in Grades K-12. In:Allensworth D Lawson E Nicholson L Wyche J, editors. Schools and Health: Our Nation's Investment. Washington, DC: The National Academies Press (1997). Available at: (accessed 03.12.2020).

[143] Besnier E Thomson K Stonkute D Mohammad T Akhter N. Which public health interventions are effective in reducing morbidity, mortality and health inequalities from infectious diseases amongst children in low- and middle-income countries (LMICs): an umbrella review. PLoS ONE. (2021) 16:e0251905. 10.1371/journal.pone.0251905 34111134 PMC8191901

[144] Fredriksen PM Hjelle OP Mamen A Meza TJ Westerberg AC. The health oriented pedagogical project (HOPP) a controlled longitudinal school-based physical activity intervention program. BMC Public Health. (2017) 17:370. 10.1186/s12889-017-4282-z 28454531 PMC5410047

[145] Metcalf B Henley W Wilkin T. Effectiveness of intervention on physical activity of children: systematic review and meta-analysis of controlled trials with objectively measured outcomes (EarlyBird 54). BMJ. (2012) 345:e5888. 10.1136/bmj.e5888 23044984

[146] Moreno JP Vaughan E Hernandez D Cameron RT Foreyt JP Johnston CA. The impact of acculturation level on weight status and weight outcomes in hispanic children. J Racial Ethn Health Disparities. (2016) 3:582–9. 10.1007/s40615-015-0177-9 27294753

[147] Yoo S. Dynamic energy balance and obesity prevention. J Obes Metab Syndr. (2018) 27:203–12. 10.7570/jomes.2018.27.4.203 31089565 PMC6513301

[148] Fairclough S Strattona G. Effects of a physical education intervention to improve student activity levels. Phys Educ Sport Peda. (2006) 11:29–44. 10.1080/17408980500467613

[149] Barnard ND Goldman DM Loomis JF Kahleova H Levin SM Neabore S. Plant-based diets for cardiovascular safety and performance in endurance sports. Nutrients. (2019) 11:130. 10.3390/nu11010130 30634559 PMC6356661

[150] Barnard N Kahleova H Levin SM. The use of plant-based diets for obesity treatment. Int J Dis Reversal Prev. (2019) 1:12. 10.22230/ijdrp.2019v1n1a11

[151] Merghani A Maestrini V Rosmini S Cox AT Dhutia H Bastiaenan R. Prevalence of subclinical coronary artery disease in masters endurance athletes with a low atherosclerotic risk profile. Circulation. (2017) 136:126–37. 10.1161/CIRCULATIONAHA.116.026964 28465287

[152] Schwartz RS Kraus SM Schwartz JG Wickstrom KK Peichel G Garberich RF. Increased coronary artery plaque volume among male marathon runners. Mo Med. (2014) 111:89–94. Available online at: https://europepmc.org/article/med/30323509 30323509 PMC6179497

[153] Sheppard MN. The fittest person in the morgue? Histopathology. (2012) 60:381–96. 10.1111/j.1365-2559.2011.03852.x 21668469

[154] Benatar JR Stewart R. Cardiometabolic risk factors in vegans; A meta-analysis of observational studies. PLoS ONE. (2018) 13:e0209086. 10.1371/journal.pone.0209086 30571724 PMC6301673

[155] Kahleova H Dort S Holubkov R Barnard ND. A plant-based high-carbohydrate, low-fat diet in overweight individuals in a 16-week randomized clinical trial: the role of carbohydrates. Nutrients. (2018) 10:1302. 10.3390/nu10091302 30223451 PMC6165066

[156] Mishra S Xu J AgarwalU Gonzales J Levin S Barnard ND. A multicenter randomized controlled trial of a plant-based nutrition program to reduce body weight and cardiovascular risk in the corporate setting: the GEICO study. Eur J Clin Nutr. (2013) 67:718–24. 10.1038/ejcn.2013.92 23695207 PMC3701293

[157] Le LT Sabaté J. Beyond meatless, the health effects of vegan diets: findings from the Adventist cohorts. Nutrients. (2014) 6:2131–47. 10.3390/nu6062131 24871675 PMC4073139

[158] Weder S Hoffmann M Becker K Alexy U Keller M. Energy, macronutrient intake, and anthropometrics of vegetarian, vegan, and omnivorous children (1-3 years) in Germany (VeChi Diet Study). Nutrients. (2019) 11:832. 10.3390/nu11040832 31013738 PMC6521189

[159] Centers for Disease Control and Prevention. Body Mass Index (BMI) (2021). Available online at: https://www.cdc.gov/healthyweight/assessing/bmi/index.html

[160] Peretti N Darmaun D Chouraqui JP Bocquet A Briend A Feillet F. Vegetarian diet in children and adolescents: a health benefit? Arch Pediatr. (2020) 27:173–5. 10.1016/j.arcped.2020.03.010 32331916

[161] Melina V Craig W Levin S. Position of the Academy of Nutrition and Dietetics: Vegetarian Diets. J Acad Nutr Diet. (2016) 115:1970–80. 10.1016/j.jand.2016.09.025 27886704

[162] Academy of Nutrition and Dietetics (AND) *2017: Feeding Vegetarian and Vegan Infants and Toddlers*. Available online at: https://www.eatright.org/food/nutrition/vegetarianand-special-diets/feeding-vegetarian-and-vegan-infants-and-toddlers (accessed March 21, 2018).

[163] Physicians Committee for Responsible Medicine (PCRM) *2021: Nutrition for Kids – Plant-Based Diets for Infants Children and Teens*. Available online at: https://www.pcrm.org/good-nutrition/nutrition-for-kids (accessed September 21, 2021).

[164] Schüpbach R Wegmüller R Berguerand C Bui M Herter-Aeberli I. Micronutrient status and intake in omnivores, vegetarians and vegans in Switzerland. Eur J Nutr. (2017) 56:283–93. 10.1007/s00394-015-1079-7 26502280

[165] Greger M. Omnivore vs. Vegan Nutrient Deficiencies. (2011). Available online at: www.youtube.com/watch?v$=$VVJCHVEatqY (accessed March 18, 2018).

[166] Turner DR Sinclair WH Knez WL. Nutritional adequacy of vegetarian and omnivore dietary intakes. J Nutr Health Sci. (2014) 1:201. 10.15744/2393-9060.1.20115153271

[167] Bakaloudi DR Halloran A Rippin HL Oikonomidou AC Dardavesis TI Williams J. Intake and adequacy of the vegan diet. A systematic review of the evidence. Clin Nutr. (2021) 40:3503–21. 10.1016/j.clnu.2020.11.035 33341313

[168] Weikert C Trefflich I Menzel J Obeid R Longree A Dierkes J. Vitamin and mineral status in a vegan diet. Dtsch Arztebl Int. (2020) 117:575–82. 10.3238/arztebl.2020.0575 33161940 PMC7779846

[169] Alexy U Fischer M Weder S Längler A Michalsen A Keller M. Food group intake of children and adolescents (6–18 years) on a vegetarian, vegan, or omnivore diet: results of the VeChi Youth Study. Br J Nutr. (2021) 1–26. 10.1017/S0007114521003603. [Epub ahead of print]. 34511141

[170] Alexy U Fischer M Weder S Längler A Michalsen A Sputtek A. Nutrient intake and status of german children and adolescents consuming vegetarian, vegan or omnivore diets: results of the VeChi youth study. Nutrients. (2021) 13:1707. 10.3390/nu13051707 34069944 PMC8157583

[171] Weder S Keller M Fischer M Becker K Alexy U. Intake of micronutrients and fatty acids of vegetarian, vegan, and omnivorous children (1–3 years) in Germany (VeChi diet study). Eur J Nutr. (2022) 61:1507–20. 10.1007/s00394-021-02753-3 34855006 PMC8921058

[172] Sdona E Ekstrom S Andersson N Gortmaker SL. Fruit, vegetable and dietary antioxidant intake in school age, respiratory health up to young adulthood. Clin Exp Allergy. (2021) 52:104–14. 10.1111/cea.14020 34549838

[173] Kenney EL Long MW Cradock AL Gortmaker SL. Prevalence of inadequate hydration among US children and disparities by gender and race/ethnicity: National health and nutrition examination survey, 2009–2012. Am J Public Health. (2015) *105*:113–8. 10.2105/AJPH.2015.302572 26066941 PMC4504329

[174] Janssen I Leblanc AG. Systematic review of the health benefits of physical activity and fitness in school-aged children and youth. Int J Behav Nutr Phys Act. (2010) 7:40. 10.1186/1479-5868-7-40 20459784 PMC2885312

[175] World Health Organization. Global status report on non-communicable diseases. (2010). Available online at: https://www.who.int/nmh/publications/ncd_report_full_en.pdf (accessed January 20, 2020).

[176] Lynch J Smith GD. A life course approach to chronic disease epidemiology. Annu Rev Public Health. (2005) 26:1–35. 10.1146/annurev.publhealth.26.021304.144505 15760279

[177] Thivel D Tremblay MS Katzmarzyk PT Fogelholm M Hu G Maher C. Associations between meeting combinations of 24-hours movement recommendations and dietary patterns of children: a 12-country study. Prev Med. (2019) 118:159–65. 10.1016/j.ypmed.2018.10.025 30393016

[178] European Commission – State of Health in the EU (2019). Available online at: https://ec.europa.eu/commission/presscorner/detail/en/IP_19_6336 (accessed December 03, 2020).

[179] American Medical Association (AMA) 2017: House of Delegates Healthful Hospitals. Reference Committee D. Resolution 406 (A-17): p. 5;37. District of Columbia. Annual Conference, Chicago. Available online at: https://www.ama-assn.org/sites/default/files/mediabrowser/public/yps/a17-yps-d-final-grid.pdf. (page 5), Available online at: https://www.amaassn.org/sites/default/files/media-browser/public/hod/a17-refcomm-d.pdf. (page 37), Available online at: http://inourishgently.com/american-medical-association-tells-hospitals-go-vegan-banmeat-dairy/ (accessed March 21, 2018).

[180] Albert Schweitzer Stiftung. Portugal: Vegane Speisen sind in Kantinen Pflicht. (2017). Available at Available online at: https://albert-schweitzer-stiftung.de/aktuell/portugal-vegane-speisen-inkantinen-pflicht (accessed September 13, 2021).

[181] Kudelka P Hnat F Müller-Amenitsch R. Vegan im Recht. Das Handbuch für juristische Fragen des veganen Lebensstils. Österreich Edition. Kapitel 3.1 – Veganes Essen in der Schule. Wien: Vegane Gesellschaft Österreich (2017)

[182] McIver JP Carmines EG. Unidimensional scaling. Quantitative Applications in the Social Sciences. Beverly Hills: Sage Publications (1981). 10.4135/9781412986441

[183] Hoeppner BB Kelly JF Urbanoski KA Slaymaker V. Comparative utility of a single-item versus multiple-item measure of self-efficacy in predicting relapse among young adults. J Subst Abuse Treat. (2011) 41:305–12. 10.1016/j.jsat.2011.04.005 21700411 PMC3315352

[184] Cocca A Niedermeier M Prünster V Wirnitzer K Drenowatz C Greier K. Self-rated health status of upper secondary school pupils and its associations with multiple health-related factors. Int J Environ Res Public Health. (2022) 19:6947. 10.3390/ijerph19116947 35682529 PMC9180056

[185] Carreras M Puig G Sánchez-Pérez I Inoriza JM Coderch J Gispert R. Morbidity and self-perception of health, two different approaches to health status. Gac Sanit. (2020) 34:601–7. 10.1016/j.gaceta.2019.04.005 31255397

[186] Rolstad S Adler J Ryden A. Response burden and questionnaire length: is shorter better? A review and meta-analysis. Value Health. (2011) 14:1101–8. 10.1016/j.jval.2011.06.003 22152180

