# Supplementary material for: Study protocol of “From Science 2 School”—prevalence of sports and physical exercise linked to omnivorous, vegetarian and vegan, diets among Austrian secondary schools
Source: Front Sports Act Living. 2022 Sep 28;4:967915. doi: 10.3389/fspor.2022.967915 (PMC9554638; doi:10.3389/fspor.2022.967915)
Supplement: Supplementary file 1 [file Data_Sheet_1.pdf]

From Science 2 School - Fragebogen Schüler_innen

Herzlich willkommen bei der Online-Umfrage zur Schulstudie

‚From Science 2 School: Nachhaltig gesund – bewegt & veggie‘!

In dieser Umfrage sind 85 Fragen enthalten.

TEIL A – Zur PERSON

1. Deine Nationalität? *

Bitte wählen Sie nur eine der folgenden Antworten aus:

- ◯ Österreich
- ◯ andere

1.1 Deine Nationalität: Andere? *

Beantworten Sie diese Frage nur, wenn folgende Bedingungen erfüllt sind:
Antwort war 'andere' bei Frage '1 [Nationalitaet1]' (1. Deine Nationalität?)

Bitte wählen Sie nur eine der folgenden Antworten aus:

- ◯ Afghanistan
- ◯ Ägypten
- ◯ Albanien
- ◯ Algerien
- ◯ Andorra
- ◯ Angola
- ◯ Antigua und Barbuda
- ◯ Äquatorialguinea
- ◯ Argentinien
- ◯ Armenien
- ◯ Aserbaidschan
- ◯ Äthiopien
- ◯ Australien
- ◯ Bahamas
- ◯ Bahrain
- ◯ Bangladesch
- ◯ Barbados
- ◯ Belgien
- ◯ Belize
- ◯ Benin
- ◯ Bhutan
- ◯ Bolivien
- ◯ Bosnien und Herzegowina
- ◯ Botsuana
- ◯ Brasilien
- ◯ Brunei Darussalam
- ◯ Bulgarien
- ◯ Burkina Faso
- ◯ Burundi
- ◯ Chile

- China
- Costa Rica
- Côte d'Ivoire
- Dänemark
- Demokratische Republik Kongo
- Deutschland
- Dominica
- Dominikanische Republik
- Dschibuti
- Ecuador
- El Salvador
- Eritrea
- Estland
- Eswatini
- Fidschi
- Finnland
- Föderierte Staaten Mikronesien
- Frankreich
- Gabun
- Gambia
- Georgien
- Ghana
- Grenada
- Griechenland
- Guatemala
- Guinea
- Guinea-Bissau
- Guyana
- Haiti
- Honduras
- Indien
- Indonesien
- Irak
- Iran
- Irland

- ◯ Island
- ◯ Israel
- ◯ Italien
- ◯ Jamaika
- ◯ Japan
- ◯ Jemen
- ◯ Jordanien
- ◯ Kambodscha
- ◯ Kamerun
- ◯ Kanada
- ◯ Kap Verde
- ◯ Kasachstan
- ◯ Katar
- ◯ Kenia
- ◯ Kirgisistan
- ◯ Kiribati
- ◯ Kolumbien
- ◯ Komoren
- ◯ Demokratische Volksrepublik Korea
- ◯ Republik Korea
- ◯ Kroatien
- ◯ Kuba
- ◯ Kuwait
- ◯ Laos
- ◯ Lesotho
- ◯ Lettland
- ◯ Libanon
- ◯ Liberia
- ◯ Libyen
- ◯ Liechtenstein
- ◯ Litauen
- ◯ Luxemburg
- ◯ Madagaskar
- ◯ Malawi
- ◯ Malaysia

- ◯ Malediven
- ◯ Mali
- ◯ Malta
- ◯ Marokko
- ◯ Marshallinsel
- ◯ Mauretanien
- ◯ Mauritius
- ◯ Mazedonien
- ◯ Mexiko
- ◯ Moldau
- ◯ Monaco
- ◯ Mongolei
- ◯ Montenegro
- ◯ Mosambik
- ◯ Myanmar
- ◯ Namibia
- ◯ Nauru
- ◯ Nepal
- ◯ Neuseeland
- ◯ Nicaragua
- ◯ Niederlande
- ◯ Niger
- ◯ Nigeria
- ◯ Norwegen
- ◯ Oman
- ◯ Osttimor
- ◯ Pakistan
- ◯ Palau
- ◯ Panama
- ◯ Papua-Neuguinea
- ◯ Paraguay
- ◯ Peru
- ◯ Philippinen
- ◯ Polen
- ◯ Portugal

- Republik Kongo
- Ruanda
- Rumänien
- Russische Föderation
- Saint Kitts und Nevis
- Saint Lucia
- Salomonen
- Sambia
- Samoa
- San Marino
- São Tomé und Príncipe
- Saudi-Arabien
- Schweden
- Schweiz
- Senegal
- Serbien
- Seychellen
- Sierra Leone
- Simbabwe
- Singapur
- Slowakei
- Slowenien
- Somalia
- Spanien
- Sri Lanka
- St. Vincent und die Grenadinen
- Südafrika
- Sudan
- Südsudan
- Suriname
- Syrien
- Tadschikistan
- Tansania
- Thailand
- Togo

- Tonga
- Trinidad und Tobago
- Tschad
- Tschechische Republik
- Tunesien
- Türkei
- Turkmenistan
- Tuvalu
- Uganda
- Ukraine
- Ungarn
- Uruguay
- Usbekistan
- Vanuatu
- Vatikanstaat
- Venezuela
- Vereinigte Arabische Emirate
- Vereinigte Staaten von Amerika
- Vereinigtes Königreich
- Vietnam
- Weißrussland
- Zentralafrikanische Republik
- Zypern

2. Dein Wohnort? *

Bitte wählen Sie nur eine der folgenden Antworten aus:

- Stadt/städtische Region
- Land/ländliche Region

2.1 Dein Bundesland? *

Bitte wählen Sie nur eine der folgenden Antworten aus:

- ◯ Burgenland
- ◯ Kärnten
- ◯ Niederösterreich
- ◯ Oberösterreich
- ◯ Salzburg
- ◯ Steiermark
- ◯ Tirol
- ◯ Vorarlberg
- ◯ Wien

3. Dein Geschlecht? *

Bitte wählen Sie nur eine der folgenden Antworten aus:

- ◯ männlich
- ◯ weiblich

4. Deine Klasse (Schulstufe)? *

Bitte wählen Sie nur eine der folgenden Antworten aus:

- ◯ Unterstufe (=Sekundarstufe 1)
- ◯ Oberstufe (=Sekundarstufe 2)

4.1 Unterstufe (=Sekundarstufe 1) *

Beantworten Sie diese Frage nur, wenn folgende Bedingungen erfüllt sind:
Antwort war 'Unterstufe (=Sekundarstufe 1)' bei Frage '6 [KlasseSchulstufe]' (4. Deine Klasse (Schulstufe)?)

Bitte wählen Sie nur eine der folgenden Antworten aus:

- ◯ 1. Klasse (5. Schulstufe)
- ◯ 2. Klasse (6. Schulstufe)
- ◯ 3. Klasse (7. Schulstufe)
- ◯ 4. Klasse (8. Schulstufe)

4.1 Oberstufe (=Sekundarstufe 2) *

Beantworten Sie diese Frage nur, wenn folgende Bedingungen erfüllt sind:
Antwort war 'Oberstufe (=Sekundarstufe 2)' bei Frage '6 [KlasseSchulstufe]' (4. Deine Klasse (Schulstufe)?)

Bitte wählen Sie nur eine der folgenden Antworten aus:

- ◯ 1. Klasse inkl. Polytechnische Schule (9. Schulstufe)
- ◯ 2. Klasse (10. Schulstufe)
- ◯ 3. Klasse (11. Schulstufe)
- ◯ 4. Klasse (12. Schulstufe)
- ◯ 5. Klasse (13. Schulstufe)

5. Besuchst Du eine Sport-Klasse (d. h. eine Klasse mit sportlichem Schwerpunkt)? *

Bitte wählen Sie nur eine der folgenden Antworten aus:

- ◯ Ja
- ◯ Nein

6. Dein Schultyp? *

Beantworten Sie diese Frage nur, wenn folgende Bedingungen erfüllt sind:
Antwort war 'Unterstufe (=Sekundarstufe 1)' bei Frage '6 [KlasseSchulstufe]' (4. Deine Klasse (Schulstufe)?)

Bitte wählen Sie nur eine der folgenden Antworten aus:

- ◯ MS – Mittelschule (vorher: NMS – Neue Mittelschule)
- ◯ AHS – Allgemeinbildende Höhere Schule (z. B. Gymnasium Unterstufe)
- ◯ andere ______

6. Dein Schultyp? *

Beantworten Sie diese Frage nur, wenn folgende Bedingungen erfüllt sind:
Antwort war 'Oberstufe (=Sekundarstufe 2)' bei Frage '6 [KlasseSchulstufe]' (4. Deine Klasse (Schulstufe)?)

Bitte wählen Sie nur eine der folgenden Antworten aus:

- ◯ Polytechnische Schule
- ◯ BMS – Berufsbildende Mittlere Schulen (Fachschulen wie z. B. Handelsschule (HAS) bzw. 3- oder 4-jährig an HBLA oder HTL, etc.)
- ◯ AHS – Allgemeinbildende Höhere Schule (z. B. Gymnasium Oberstufe)
- ◯ BHS – Berufsbildende Höhere Schulen mit Matura (z. B. 5-jährig an HAK, HBLA, HTL usw.)
- ◯ Berufsschule
- ◯ andere ______

7. Dein Körpergewicht (kg)?

Beispiel:
40,3 kg oder 37,80 kg
51 kg oder 85,0 kg

*

❗ Ihre Antwort muss zwischen 20 und 160 liegen.

Bitte geben Sie Ihre Antwort hier ein:

kg

Nur Zahlen-Eingabe möglich (zwischen 20,0 kg und 160,0 kg)!

8. Deine Körpergröße (m)? *

Bitte wählen Sie nur eine der folgenden Antworten aus:

- ◯ 1,00
- ◯ 1,01
- ◯ 1,02
- ◯ 1,03
- ◯ 1,04
- ◯ 1,05
- ◯ 1,06
- ◯ 1,07
- ◯ 1,08
- ◯ 1,09
- ◯ 1,10
- ◯ 1,11
- ◯ 1,12
- ◯ 1,13
- ◯ 1,14
- ◯ 1,15
- ◯ 1,16
- ◯ 1,17
- ◯ 1,18
- ◯ 1,19
- ◯ 1,20
- ◯ 1,21
- ◯ 1,22
- ◯ 1,23
- ◯ 1,24
- ◯ 1,25
- ◯ 1,26
- ◯ 1,27
- ◯ 1,28
- ◯ 1,29

- ◯ 1,30
- ◯ 1,31
- ◯ 1,32
- ◯ 1,33
- ◯ 1,34
- ◯ 1,35
- ◯ 1,36
- ◯ 1,37
- ◯ 1,38
- ◯ 1,39
- ◯ 1,40
- ◯ 1,41
- ◯ 1,42
- ◯ 1,43
- ◯ 1,44
- ◯ 1,45
- ◯ 1,46
- ◯ 1,47
- ◯ 1,48
- ◯ 1,49
- ◯ 1,50
- ◯ 1,51
- ◯ 1,52
- ◯ 1,53
- ◯ 1,54
- ◯ 1,55
- ◯ 1,56
- ◯ 1,57
- ◯ 1,58
- ◯ 1,59
- ◯ 1,60
- ◯ 1,61
- ◯ 1,62
- ◯ 1,63
- ◯ 1,64

- ◯ 1,65
- ◯ 1,66
- ◯ 1,67
- ◯ 1,68
- ◯ 1,69
- ◯ 1,70
- ◯ 1,71
- ◯ 1,72
- ◯ 1,73
- ◯ 1,74
- ◯ 1,75
- ◯ 1,76
- ◯ 1,77
- ◯ 1,78
- ◯ 1,79
- ◯ 1,80
- ◯ 1,81
- ◯ 1,82
- ◯ 1,83
- ◯ 1,84
- ◯ 1,85
- ◯ 1,86
- ◯ 1,87
- ◯ 1,88
- ◯ 1,89
- ◯ 1,90
- ◯ 1,91
- ◯ 1,92
- ◯ 1,93
- ◯ 1,94
- ◯ 1,95
- ◯ 1,96
- ◯ 1,97
- ◯ 1,98
- ◯ 1,99

- ◯ 2,00
- ◯ 2,01
- ◯ 2,02
- ◯ 2,03
- ◯ 2,04
- ◯ 2,05
- ◯ 2,06
- ◯ 2,07
- ◯ 2,08
- ◯ 2,09
- ◯ 2,10
- ◯ 2,11
- ◯ 2,12
- ◯ 2,13
- ◯ 2,14
- ◯ 2,15
- ◯ 2,16
- ◯ 2,17
- ◯ 2,18
- ◯ 2,19
- ◯ 2,20

9. Nimmst Du am Schulsport-Angebot teil?

Anmerkung:
Hier ist nicht der Unterricht Bewegung & Sport laut Stundenplan gemeint, sondern der zusätzliche Sport- & Bewegungs-Unterricht, den Du an der Schule besuchst!

*

Bitte wählen Sie nur eine der folgenden Antworten aus:

- ◯ Ja
- ◯ Nein

9.1 Ja, ich nehme am Schulsport-Angebot teil: *Anmerkung: Mehrfachnennung möglich!* *

Beantworten Sie diese Frage nur, wenn folgende Bedingungen erfüllt sind:
Antwort war 'Ja' bei Frage '14 [SchulsportAngebot]' (9. Nimmst Du am Schulsport-Angebot teil? Anmerkung: Hier ist nicht der Unterricht Bewegung & Sport laut Stundenplan gemeint, sondern der zusätzliche Sport- & Bewegungs-Unterricht, den Du an der Schule besuchst!)

Bitte wählen Sie alle zutreffenden Antworten aus:

- ☐ verbindliche Übung Bewegung & Sport
- ☐ Freifach Bewegung & Sport (Freigegenstand)
- ☐ unverbindliche Übung Bewegung & Sport

10. Dein Alter? *

Beantworten Sie diese Frage nur, wenn folgende Bedingungen erfüllt sind:
Antwort war 'Unterstufe (=Sekundarstufe 1)' bei Frage '6 [KlasseSchulstufe]' (4. Deine Klasse (Schulstufe)?)

Bitte wählen Sie nur eine der folgenden Antworten aus:

- ○ 9
- ○ 10
- ○ 11
- ○ 12
- ○ 13
- ○ 14
- ○ 15
- ○ 16

10. Dein Alter? *

Beantworten Sie diese Frage nur, wenn folgende Bedingungen erfüllt sind:
Antwort war 'Oberstufe (=Sekundarstufe 2)' bei Frage '6 [KlasseSchulstufe]' (4. Deine Klasse (Schulstufe)?)

Bitte wählen Sie nur eine der folgenden Antworten aus:

- ○ 14
- ○ 15
- ○ 16
- ○ 17
- ○ 18
- ○ 19
- ○ 20
- ○ 21
- ○ 22

11. Dein Stimmungs-Barometer *

Bitte wählen Sie die zutreffende Antwort für jeden Punkt aus:

	Ja, stimmt genau	Stimmt eher	Stimmt weniger	Nein, stimmt nicht
**Ich betreibe gerne Bewegung & Sport, weil es mir Spaß macht und gesund ist.**	○	○	○	○
**Ich interessiere mich für meine Gesundheit und informiere mich.**	○	○	○	○
**Ich interessiere mich für Ernährung, was gesund bzw. was ungesund ist.**	○	○	○	○

TEIL B – BEWEGUNG & SPORT

1. Betreibst Du Sport in Deiner Freizeit? *

Bitte wählen Sie nur eine der folgenden Antworten aus:

- ◯ Ja
- ◯ Nein

1.1 Warum betreibst Du keinen Sport in Deiner Freizeit?

Beantworten Sie diese Frage nur, wenn folgende Bedingungen erfüllt sind:
Antwort war 'Nein' bei Frage '19 [BetreibstDuSport]' (1. Betreibst Du Sport in Deiner Freizeit?)

Bitte geben Sie Ihre Antwort hier ein:

2. Das ist mein Beweggrund für Bewegung & Sport, das ist mir wichtig ...
2.1 Das ist mein **TOP 1 (wichtigster) Beweggrund** für Bewegung & Sport
Anmerkung:
Nur 1 Antwort möglich! *

Beantworten Sie diese Frage nur, wenn folgende Bedingungen erfüllt sind:
Antwort war 'Ja' bei Frage '19 [BetreibstDuSport]' (1. Betreibst Du Sport in Deiner Freizeit?)

Bitte wählen Sie alle zutreffenden Antworten aus:

- ☐ Eigenes Interesse: macht mir Freude/Spaß
- ☐ Eltern/Geschwister/Familie (inkl. Haustier, z. B. Hund)
- ☐ Freunde/Freund_in
- ☐ Lehrer_innen/Schulkolleg_innen (Schule)
- ☐ Sportverein
- ☐ Vorbilder (z. B. Sportler, Promis in Medien)
- ☐ Gesundheit/Wohlbefinden

- ☐ Leistung verbessern
- ☐ Sich mit anderen messen (z. B. Wettkampf)
- ☐ Anerkennung
- ☐ Nervenkitzel/Abenteuer/Erlebnis (Adrenalinkick)
- ☐ Fitness (z. B. kräftig, ausdauernd, wendig)
- ☐ Aussehen/Ästhetischer Körper (gute Figur z. B. schlank, schön, definierte Muskeln)
- ☐ Entspannung/Ausgleich
- ☐ Druck und Stress abbauen
- ☐ Lifestyle (spezielles Outfit, z. B. Tanz, Skateboard, Snowboard)
- ☐ Kein bestimmter Grund
- ☐ Andere

2.1.1 Andere: *

Beantworten Sie diese Frage nur, wenn folgende Bedingungen erfüllt sind:
Antwort war bei Frage '21 [TOP1Beweggr1]' (2. Das ist mein Beweggrund für Bewegung & Sport, das ist mir wichtig ... 2.1 Das ist mein TOP 1 (wichtigster) Beweggrund für Bewegung & Sport Anmerkung: Nur 1 Antwort möglich!)

Bitte geben Sie Ihre Antwort hier ein:

2. Das ist mein Beweggrund für Bewegung & Sport, das ist mir wichtig ...
2.2 Das ist mein **TOP 2 Beweggrund** für Bewegung & Sport
Anmerkung:
Nur 1 Antwort möglich! *

Beantworten Sie diese Frage nur, wenn folgende Bedingungen erfüllt sind:
Antwort war 'Ja' bei Frage '19 [BetreibstDuSport]' (1. Betreibst Du Sport in Deiner Freizeit?)

Bitte wählen Sie alle zutreffenden Antworten aus:

- ☐ Eigenes Interesse: macht mir Freude/Spaß
- ☐ Eltern/Geschwister/Familie (inkl. Haustier, z. B. Hund)
- ☐ Freunde/Freund_in
- ☐ Lehrer_innen/Schulkolleg_innen (Schule)
- ☐ Sportverein
- ☐ Vorbilder (z. B. Sportler, Promis in Medien)
- ☐ Gesundheit/Wohlbefinden
- ☐ Leistung verbessern
- ☐ Sich mit anderen messen (z. B. Wettkampf)
- ☐ Anerkennung
- ☐ Nervenkitzel/Abenteuer/Erlebnis (Adrenalinkick)
- ☐ Fitness (z. B. kräftig, ausdauernd, wendig)
- ☐ Aussehen/Ästhetischer Körper (gute Figur z. B. schlank, schön, definierte Muskeln)
- ☐ Entspannung/Ausgleich
- ☐ Druck und Stress abbauen
- ☐ Lifestyle (spezielles Outfit, z. B. Tanz, Skateboard, Snowboard)
- ☐ Kein bestimmter Grund

- ☐ Andere

2.2.1 Andere: *

Beantworten Sie diese Frage nur, wenn folgende Bedingungen erfüllt sind:
Antwort war bei Frage '23 [TOP2Beweggr2]' (2. Das ist mein Beweggrund für Bewegung & Sport, das ist mir wichtig ... 2.2 Das ist mein TOP 2 Beweggrund für Bewegung & Sport Anmerkung: Nur 1 Antwort möglich!)

Bitte geben Sie Ihre Antwort hier ein:

2. Das ist mein Beweggrund für Bewegung & Sport, das ist mir wichtig ...
2.3 Das ist mein **TOP 3 Beweggrund** für Bewegung & Sport
Anmerkung:
Nur 1 Antwort möglich! *

Beantworten Sie diese Frage nur, wenn folgende Bedingungen erfüllt sind:
Antwort war 'Ja' bei Frage '19 [BetreibstDuSport]' (1. Betreibst Du Sport in Deiner Freizeit?)

Bitte wählen Sie alle zutreffenden Antworten aus:

- [ ] Eigenes Interesse: macht mir Freude/Spaß
- [ ] Eltern/Geschwister/Familie (inkl. Haustier, z. B. Hund)
- [ ] Freunde/Freund_in
- [ ] Lehrer_innen/Schulkolleg_innen (Schule)
- [ ] Sportverein
- [ ] Vorbilder (z. B. Sportler, Promis in Medien)
- [ ] Gesundheit/Wohlbefinden
- [ ] Leistung verbessern
- [ ] Sich mit anderen messen (z. B. Wettkampf)
- [ ] Anerkennung
- [ ] Nervenkitzel/Abenteuer/Erlebnis (Adrenalinkick)
- [ ] Fitness (z. B. kräftig, ausdauernd, wendig)
- [ ] Aussehen/Ästhetischer Körper (gute Figur z. B. schlank, schön, definierte Muskeln)
- [ ] Entspannung/Ausgleich
- [ ] Druck und Stress abbauen
- [ ] Lifestyle (spezielles Outfit, z. B. Tanz, Skateboard, Snowboard)
- [ ] Kein bestimmter Grund

- [ ] Andere

2.3.1 Andere: *

Beantworten Sie diese Frage nur, wenn folgende Bedingungen erfüllt sind:
Antwort war bei Frage '25 [TOP3Beweggr3]' (2. Das ist mein Beweggrund für Bewegung & Sport, das ist mir wichtig ... 2.3 Das ist mein TOP 3 Beweggrund für Bewegung & Sport Anmerkung: Nur 1 Antwort möglich!)

Bitte geben Sie Ihre Antwort hier ein:

3. Wie lange schon betreibst Du Sport als Freizeitaktivität?*

Beantworten Sie diese Frage nur, wenn folgende Bedingungen erfüllt sind:
Antwort war 'Ja' bei Frage '19 [BetreibstDuSport]' (1. Betreibst Du Sport in Deiner Freizeit?)

Bitte wählen Sie nur eine der folgenden Antworten aus:

- ◯ schon immer, mein Leben lang
- ◯ ≤ 0,5 Jahr (1 – 6 Monate)
- ◯ 1 Jahr
- ◯ 2 Jahre
- ◯ 3 – 5 Jahre
- ◯ 6 – 9 Jahre
- ◯ 10 oder mehr Jahre

4. Mit wem betreibst Du am häufigsten Sport in Deiner Freizeit? *

Beantworten Sie diese Frage nur, wenn folgende Bedingungen erfüllt sind:
Antwort war 'Ja' bei Frage '19 [BetreibstDuSport]' (1. Betreibst Du Sport in Deiner Freizeit?)

Bitte wählen Sie nur eine der folgenden Antworten aus:

- ◯ Ich alleine
- ◯ Eltern/Geschwister/Familie (inkl. Haustier, z. B. Hund)
- ◯ Freunde/Freund_in
- ◯ Mannschaft/Teamkolleg_innen (z. B. Fußballverein, Tanzgruppe)
- ◯ Trainingspartner_in (wenige, z. B. zu zweit, zu dritt)
- ◯ Trainer_in/Coach

5. Welche Sportart(en) betreibst Du in Deiner Freizeit? *Anmerkung: Mehrfachnennung möglich!* *

Beantworten Sie diese Frage nur, wenn folgende Bedingungen erfüllt sind:
Antwort war 'Ja' bei Frage '19 [BetreibstDuSport]' (1. Betreibst Du Sport in Deiner Freizeit?)

Bitte wählen Sie alle zutreffenden Antworten aus:

- ☐ Einzelsportart
- ☐ Mannschafts-/Teamsportart

5.1 Welche Einzelsportart(en) betreibst Du in Deiner Freizeit?
Anmerkung:
Mehrfachnennung möglich! *

Beantworten Sie diese Frage nur, wenn folgende Bedingungen erfüllt sind:
Antwort war 'Ja' bei Frage '19 [BetreibstDuSport]' (1. Betreibst Du Sport in Deiner Freizeit?)
und Antwort war bei Frage '29 [EinzelMannsch]' (5. Welche Sportart(en) betreibst Du in Deiner Freizeit? Anmerkung: Mehrfachnennung möglich!)

Bitte wählen Sie alle zutreffenden Antworten aus:

- ☐ Schwimmen
- ☐ Laufen
- ☐ Radfahren
- ☐ Jazz Dance/Hip Hop
- ☐ Skifahren
- ☐ Skateboarden
- ☐ Fitness-/Krafttraining
- ☐ Sonstiges: ____________________

5.2 Welche Mannschafts-/Teamsportart(en) betreibst Du in Deiner Freizeit?
Anmerkung:
Mehrfachnennung möglich! *

Beantworten Sie diese Frage nur, wenn folgende Bedingungen erfüllt sind:
Antwort war 'Ja' bei Frage '19 [BetreibstDuSport]' (1. Betreibst Du Sport in Deiner Freizeit?) *und* Antwort war bei Frage '29 [EinzelMannsch]' (5. Welche Sportart(en) betreibst Du in Deiner Freizeit? Anmerkung: Mehrfachnennung möglich!)

Bitte wählen Sie alle zutreffenden Antworten aus:

- ☐ Fußball
- ☐ Handball
- ☐ Basketball
- ☐ Volleyball
- ☐ Sonstiges: ____________

5.3 Deine Haupt-Sportart ist *

Beantworten Sie diese Frage nur, wenn folgende Bedingungen erfüllt sind:
-------- Scenario 1 --------
Antwort war 'Ja' bei Frage '19 [BetreibstDuSport]' (1. Betreibst Du Sport in Deiner Freizeit?) *und* Antwort war bei Frage '29 [EinzelMannsch]' (5. Welche Sportart(en) betreibst Du in Deiner Freizeit? Anmerkung: Mehrfachnennung möglich!)
-------- oder Scenario 2 --------
Antwort war 'Ja' bei Frage '19 [BetreibstDuSport]' (1. Betreibst Du Sport in Deiner Freizeit?) *und* Antwort war bei Frage '29 [EinzelMannsch]' (5. Welche Sportart(en) betreibst Du in Deiner Freizeit? Anmerkung: Mehrfachnennung möglich!)

Bitte wählen Sie nur eine der folgenden Antworten aus:

- ◯ Schwimmen
- ◯ Laufen
- ◯ Radfahren
- ◯ Jazz Dance/Hip Hop
- ◯ Skifahren
- ◯ Skateboarden
- ◯ Fitness-/Krafttraining
- ◯ Fußball
- ◯ Handball
- ◯ Basketball
- ◯ Volleyball
- ◯ Sonstiges ____________

6. An wie vielen Tagen pro Woche treibst Du Sport in Deiner Freizeit (Hobby, Training, und/oder Wettkampf)? *

Beantworten Sie diese Frage nur, wenn folgende Bedingungen erfüllt sind:
Antwort war 'Ja' bei Frage '19 [BetreibstDuSport]' (1. Betreibst Du Sport in Deiner Freizeit?)

Bitte wählen Sie nur eine der folgenden Antworten aus:

- ◯ 1 Tag/Woche
- ◯ 2 Tage/Woche
- ◯ 3 Tage/Woche
- ◯ 4 Tage/Woche
- ◯ 5 Tage/Woche
- ◯ 6 Tage/Woche
- ◯ 7 Tage/Woche

7. Durchschnittliche Dauer Deiner Sporteinheit (ø Stunden/Sporteinheit)? *

Beantworten Sie diese Frage nur, wenn folgende Bedingungen erfüllt sind:
Antwort war 'Ja' bei Frage '19 [BetreibstDuSport]' (1. Betreibst Du Sport in Deiner Freizeit?)

Bitte wählen Sie nur eine der folgenden Antworten aus:

- ◯ 00:15
- ◯ 00:30
- ◯ 00:45
- ◯ 01:00
- ◯ 01:15
- ◯ 01:30
- ◯ 01:45
- ◯ 02:00
- ◯ 02:15
- ◯ 02:30
- ◯ 02:45
- ◯ 03:00
- ◯ 03:15
- ◯ 03:30
- ◯ 03:45
- ◯ 04:00
- ◯ 04:15
- ◯ 04:30
- ◯ 04:45
- ◯ 05:00
- ◯ 05:15
- ◯ 05:30
- ◯ 05:45
- ◯ 06:00
- ◯ 06:15
- ◯ 06:30
- ◯ 06:45
- ◯ 07:00

- 07:15
- 07:30
- 07:45
- 08:00
- 08:15
- 08:30
- 08:45
- 09:00
- 09:15
- 09:30
- 09:45
- 10:00

8. Nimmst Du auch an sportlichen Wettkämpfen teil? *

Beantworten Sie diese Frage nur, wenn folgende Bedingungen erfüllt sind:
Antwort war 'Ja' bei Frage '19 [BetreibstDuSport]' (1. Betreibst Du Sport in Deiner Freizeit?)

Bitte wählen Sie nur eine der folgenden Antworten aus:

- Ja
- Nein

8.1 Dein Ziel bei einem sportlichen Wettkampf? *

Beantworten Sie diese Frage nur, wenn folgende Bedingungen erfüllt sind:
Antwort war 'Ja' bei Frage '19 [BetreibstDuSport]' (1. Betreibst Du Sport in Deiner Freizeit?) *und* Antwort war 'Ja' bei Frage '35 [Wettkaempfen]' (8. Nimmst Du auch an sportlichen Wettkämpfen teil?)

Bitte wählen Sie nur eine der folgenden Antworten aus:

- ◯ Durchkommen/Dabei sein ist alles!
- ◯ Freude und Spaß
- ◯ Mannschaft/Teamkolleg_innen unterstützen
- ◯ konkrete Wertung (z. B. Punkte, Zeit)
- ◯ konkrete Platzierung (z. B. Sieg, Podium, Top 10)
- ◯ Sonstiges ____________

9. Bist Du Mitglied in einem Sportverein? *

Beantworten Sie diese Frage nur, wenn folgende Bedingungen erfüllt sind:
Antwort war 'Ja' bei Frage '19 [BetreibstDuSport]' (1. Betreibst Du Sport in Deiner Freizeit?)

Bitte wählen Sie nur eine der folgenden Antworten aus:

- ◯ Ja
- ◯ Nein

TEIL C – ERNÄHRUNG

1. Wie ernährst Du Dich aktuell?
Anmerkung:
Definition Ernährungsformen in Anlehnung an die weltweit größte Fachgesellschaft für Ernährung, der Academy of Nutrition and Dietetics (AND, 2015/2016):

Art der Ernährung	JA, diese Produkte esse ich:	NEIN, diese Produkte esse ich NICHT:
**Mischkost** (ich esse alles)	**„Alles“** z. B. Fleisch inkl. Wurst, Fleischkäse/Leberkäse, Schinken, Salami, Streichwurst, Käse, Milch, Milchprodukte, Fisch und sog. „Meeresfrüchte“, Obst, Gemüse, Kartoffeln, Getreide, usw.	/
**Vegetarisch** (pflanzlich; lakto-ovo-vegetarisch)	**Milch und Milchprodukte, Eier und Eiprodukte** z. B. Käse, Topfen/Quark, Joghurt, Molke/Whey, usw.	**Fleisch, Fisch und sog. „Meeresfrüchte“** inkl. Wurst, Schinken, Salami, Fleischkäse/Leberkäse, Streichwurst, usw.
**Vegan** (rein pflanzlich)	**Pflanzliche Produkte** z. B. Obst, Gemüse, Getreide, Samen, Nüsse, Pflanzenvleisch, Pflanzenmilch, Pflanzenkäse usw.	**Keine Produkte vom Tier** z. B. kein Fleisch; keine Eier, Käse, Milch, und Milchprodukte; keinen Honig

*

Bitte wählen Sie nur eine der folgenden Antworten aus:

- ◯ Mischkost (ich esse alles)
- ◯ Vegetarisch (pflanzlich, lakto-ovo-vegetarisch)
- ◯ Vegan (rein pflanzlich)

1.1 Gibt es mindestens 1 vegetarische Speise am Schulbuffet (Jause) bzw. in der Schulkantine (Menü am Mittagstisch)? *

Beantworten Sie diese Frage nur, wenn folgende Bedingungen erfüllt sind:
Antwort war 'Vegetarisch (pflanzlich, lakto-ovo-vegetarisch)' bei Frage '38 [Ernaehrungsform]' (1. Wie ernährst Du Dich aktuell? Anmerkung: Definition Ernährungsformen in Anlehnung an die weltweit größte Fachgesellschaft für Ernährung, der Academy of Nutrition and Dietetics (AND, 2015/2016):)

Bitte wählen Sie nur eine der folgenden Antworten aus:

- ◯ Ja
- ◯ Nein
- ◯ Weiß nicht

1.2 Gibt es mindestens 1 vegane Speise am Schulbuffet (Jause) bzw. in der Schulkantine (Menü am Mittagstisch)? *

Beantworten Sie diese Frage nur, wenn folgende Bedingungen erfüllt sind:
Antwort war 'Vegan (rein pflanzlich)' bei Frage '38 [Ernaehrungsform]' (1. Wie ernährst Du Dich aktuell? Anmerkung: Definition Ernährungsformen in Anlehnung an die weltweit größte Fachgesellschaft für Ernährung, der Academy of Nutrition and Dietetics (AND, 2015/2016):)

Bitte wählen Sie nur eine der folgenden Antworten aus:

- ◯ Ja
- ◯ Nein
- ◯ Weiß nicht

2. Wie lange schon ernährst Du Dich so? *

Bitte wählen Sie nur eine der folgenden Antworten aus:

- ◯ schon immer, mein Leben lang
- ◯ ≤ 0,5 Jahr (1 – 6 Monate)
- ◯ 1 Jahr
- ◯ 2 Jahre
- ◯ 3 – 5 Jahre
- ◯ 6 – 9 Jahre
- ◯ 10 oder mehr Jahre

3. Das ist mein Beweggrund für meine aktuelle Ernährung, das ist mir wichtig ...

3.1 Das ist mein **TOP 1 (wichtigster) Beweggrund** für meine aktuelle Ernährung

Anmerkung:
Nur 1 Antwort möglich!

*

Bitte wählen Sie alle zutreffenden Antworten aus:

- [ ] Gesundheit, Wohlbefinden
- [ ] Sportliche Leistung
- [ ] Tierschutz (Tierethik)
- [ ] Klimaschutz/Umweltschutz (ökologische Aspekte)
- [ ] Welthunger (soziale Aspekte, z. B. Land, Nahrung, etc.)
- [ ] Religion/Spiritualität
- [ ] Gewohnheit /Erziehung/Tradition
- [ ] Geschmack/Genuss
- [ ] Lebensmittelskandale
- [ ] Qualität der Nahrungsmittel (z. B. biologischer Anbau)
- [ ] Billige Nahrungsmittel (finanzielle/ökonomische Aspekte)
- [ ] Eltern/Geschwister/Familie
- [ ] Freunde
- [ ] Lehrer_innen/Schulkolleg_innen (Schule)
- [ ] Vorbilder (z. B. Sportler, Popstars, Schauspieler)
- [ ] Veggie Boom/Lifestyle (chic, ‚in‘, dabei-sein, z. B. Medien, Promis)
- [ ] Kein bestimmter Grund
- [ ] Andere

3.1.1 Andere: *

Beantworten Sie diese Frage nur, wenn folgende Bedingungen erfüllt sind:
Antwort war bei Frage '42 [BeweggrundERL1]' (3. Das ist mein Beweggrund für meine aktuelle Ernährung, das ist mir wichtig ... 3.1 Das ist mein TOP 1 (wichtigster) Beweggrund für meine aktuelle Ernährung Anmerkung: Nur 1 Antwort möglich!)

Bitte geben Sie Ihre Antwort hier ein:

3. Das ist mein Beweggrund für meine aktuelle Ernährung, das ist mir wichtig ...
3.2 Das ist mein **TOP 2 Beweggrund** für meine aktuelle Ernährung
Anmerkung:
Nur 1 Antwort möglich! *

Bitte wählen Sie alle zutreffenden Antworten aus:

- ☐ Gesundheit, Wohlbefinden
- ☐ Sportliche Leistung
- ☐ Tierschutz (Tierethik)
- ☐ Klimaschutz/Umweltschutz (ökologische Aspekte)
- ☐ Welthunger (soziale Aspekte, z. B. Land, Nahrung, etc.)
- ☐ Religion/Spiritualität
- ☐ Gewohnheit /Erziehung/Tradition
- ☐ Geschmack/Genuss
- ☐ Lebensmittelskandale
- ☐ Qualität der Nahrungsmittel (z. B. biologischer Anbau)
- ☐ Billige Nahrungsmittel (finanzielle/ökonomische Aspekte)
- ☐ Eltern/Geschwister/Familie
- ☐ Freunde
- ☐ Lehrer_innen/Schulkolleg_innen (Schule)
- ☐ Vorbilder (z. B. Sportler, Popstars, Schauspieler)
- ☐ Veggie Boom/Lifestyle (chic, ‚in‘, dabei-sein, z. B. Medien, Promis)
- ☐ Kein bestimmter Grund
- ☐ Andere

3.2.1 Andere: *

Beantworten Sie diese Frage nur, wenn folgende Bedingungen erfüllt sind:
Antwort war bei Frage '44 [BeweggrundERL2]' (3. Das ist mein Beweggrund für meine aktuelle Ernährung, das ist mir wichtig ... 3.2 Das ist mein TOP 2 Beweggrund für meine aktuelle Ernährung Anmerkung: Nur 1 Antwort möglich!)

Bitte geben Sie Ihre Antwort hier ein:

3. Das ist mein Beweggrund für meine aktuelle Ernährung, das ist mir wichtig ...
3.3 Das ist mein **TOP 3 Beweggrund** für meine aktuelle Ernährung
Anmerkung:
Nur 1 Antwort möglich!

*

Bitte wählen Sie alle zutreffenden Antworten aus:

- ☐ Gesundheit, Wohlbefinden
- ☐ Sportliche Leistung
- ☐ Tierschutz (Tierethik)
- ☐ Klimaschutz/Umweltschutz (ökologische Aspekte)
- ☐ Welthunger (soziale Aspekte, z. B. Land, Nahrung, etc.)
- ☐ Religion/Spiritualität
- ☐ Gewohnheit /Erziehung/Tradition
- ☐ Geschmack/Genuss
- ☐ Lebensmittelskandale
- ☐ Qualität der Nahrungsmittel (z. B. biologischer Anbau)
- ☐ Billige Nahrungsmittel (finanzielle/ökonomische Aspekte)
- ☐ Eltern/Geschwister/Familie
- ☐ Freunde
- ☐ Lehrer_innen/Schulkolleg_innen (Schule)
- ☐ Vorbilder (z. B. Sportler, Popstars, Schauspieler)
- ☐ Veggie Boom/Lifestyle (chic, ‚in‘, dabei-sein, z. B. Medien, Promis)
- ☐ Kein bestimmter Grund
- ☐ Andere

3.3.1 Andere: *

Beantworten Sie diese Frage nur, wenn folgende Bedingungen erfüllt sind:
Antwort war bei Frage '46 [BeweggrundERL3]' (3. Das ist mein Beweggrund für meine aktuelle Ernährung, das ist mir wichtig ... 3.3 Das ist mein TOP 3 Beweggrund für meine aktuelle Ernährung Anmerkung: Nur 1 Antwort möglich!)

Bitte geben Sie Ihre Antwort hier ein:

4. Wie viel trinkst Du insgesamt pro Tag (Aufstehen/Frühstück bis zum Schlafen-Gehen)? *Anmerkung:* *0,25 Liter (bzw. 250 ml) entspricht z. B. 1 großen Tasse Tee;* *0,5 Liter (bzw. 500 ml) entspricht z. B. 1 kleinen PET-Flasche Eistee oder 1 großem Glas Apfelsaft.*

*

Bitte wählen Sie nur eine der folgenden Antworten aus:

- ◯ weniger als 1 Liter/Tag
- ◯ zwischen 1 – 1,5 Liter/Tag
- ◯ zwischen 1,5 – 2 Liter/Tag
- ◯ zwischen 2 – 2,5 Liter/Tag
- ◯ mehr als 2,5 Liter/Tag

5. Was ist Dein Lieblingsgetränk? *

Bitte wählen Sie nur eine der folgenden Antworten aus:

- ◯ Wasser (Leitungswasser, Mineralwasser, Sodawasser)
- ◯ Fruchtsäfte (verdünnt und unverdünnt, z. B. Apfelsaft, Orangensaft)
- ◯ Verdünnungssäfte (z. B. aus Himbeersirup, Holundersirup)
- ◯ Soft-Drinks (zuckerhaltige Getränke inkl. Light- und Zero-Produkte wie z. B. Eistee, Cola, Fanta, Sprite, Almdudler, usw.)
- ◯ Tee (heiß oder kalt)
- ◯ Kakao/heiße Schokolade
- ◯ Kaffee
- ◯ Energy Drinks (z. B. Red Bull, Flying Horse, etc.)

6. Was trinkst Du am häufigsten, z. B. täglich? *

Bitte wählen Sie nur eine der folgenden Antworten aus:

- ◯ Wasser (Leitungswasser, Mineralwasser, Sodawasser)
- ◯ Fruchtsäfte (verdünnt und unverdünnt, z. B. Apfelsaft, Orangensaft)
- ◯ Verdünnungssäfte (z. B. aus Himbeersirup, Holundersirup)
- ◯ Soft-Drinks (zuckerhaltige Getränke inkl. Light- und Zero-Produkte wie z. B. Eistee, Cola, Fanta, Sprite, Almdudler, usw.)
- ◯ Tee (heiß oder kalt)
- ◯ Kakao/heiße Schokolade
- ◯ Kaffee
- ◯ Energy Drinks (z. B. Red Bull, Flying Horse, etc.)

7. Isst Du täglich Obst? *

Bitte wählen Sie nur eine der folgenden Antworten aus:

- ◯ Ja
- ◯ Nein

8. Isst Du täglich Gemüse? *

Bitte wählen Sie nur eine der folgenden Antworten aus:

- ◯ Ja
- ◯ Nein

TEIL D – Gesundheit

1. Welche Nahrungsmittel denkst Du sind gesund und fördern Deine Gesundheit?
1.1 Tierische Nahrungsmittel
Anmerkung:
Mehrfachnennung möglich!

Bitte wählen Sie alle zutreffenden Antworten aus:

- ☐ Fleisch und Wurst, z. B. Schnitzel, Streichwurst, Fleischkäse/Leberkäse, Frankfurter Würstchen, Salami, Schinken, etc.
- ☐ Fisch und sog. „Meeresfrüchte“, z. B. Muscheln, Scampi, Fischöl, etc.
- ☐ Milch und Milchprodukte, z. B. Käse, Butter, Joghurt, Topfen/Quark, Sahne, etc.
- ☐ Eier
- ☐ Keine

1. Welche Nahrungsmittel denkst Du sind gesund und fördern Deine Gesundheit?
1.2 Pflanzliche Nahrungsmittel
Anmerkung:
Mehrfachnennung möglich!

Bitte wählen Sie alle zutreffenden Antworten aus:

- ☐ Obst
- ☐ Gemüse
- ☐ Getreideprodukte, z. B. Brot, Nudeln, Reis, Müsli, etc.
- ☐ Kartoffeln und Hülsenfrüchte, z. B. Bohnen, Erbsen, etc.
- ☐ Keine

1. Welche Nahrungsmittel denkst Du sind gesund und fördern Deine Gesundheit?
1.3 Nahrungsergänzungsmittel bzw. Supplemente, für z. B. Vitamine, Mineralstoffe und Spurenelemente, Protein, Ballaststoffe, etc. in Form von Tabletten/Pillen und Pulver *

Bitte wählen Sie nur eine der folgenden Antworten aus:

- ◯ Ja
- ◯ Nein
- ◯ Weiß nicht
- ◯ Manchmal (z. B. bei Mängeln, Krankheit oder wenn man viel Sport treibt)

1. Welche Nahrungsmittel denkst Du sind gesund und fördern Deine Gesundheit?
1.4 Andere Faktoren:
Anmerkung:
Mehrfachnennung möglich!

Bitte wählen Sie alle zutreffenden Antworten aus:

- ☐ Schlaf (viel, guter)
- ☐ Kein Druck und Stress
- ☐ Flüssigkeit (viel)
- ☐ Energydrinks
- ☐ Andere: ____________

1. Welche Nahrungsmittel denkst Du fördern Deine Gesundheit am meisten?
1.5.1 Das ist mein **TOP 1 (wichtigster) Faktor** für meine Gesundheit
Anmerkung:
Nur 1 Antwort möglich! *

Bitte wählen Sie alle zutreffenden Antworten aus:

- ☐ Fleisch und Wurst, z. B. Schnitzel, Streichwurst, Fleischkäse/Leberkäse, Frankfurter Würstchen, Salami, Schinken, etc.
- ☐ Fisch und sog. „Meeresfrüchte“, z. B. Muscheln, Scampi, Fischöl, etc.
- ☐ Milch und Milchprodukte, z. B. Käse, Butter, Joghurt, Topfen/Quark, Sahne, etc.
- ☐ Eier
- ☐ Obst
- ☐ Gemüse
- ☐ Getreideprodukte, z. B. Brot, Nudeln, Reis, Müsli, etc.
- ☐ Kartoffeln und Hülsenfrüchte, z. B. Bohnen, Erbsen, etc.
- ☐ Nahrungsergänzungsmittel bzw. Supplemente, für z. B. Vitamine, Mineralstoffe und Spurenelemente, Protein, Ballaststoffe, etc. in Form von Tabletten/Pillen und Pulver
- ☐ Schlaf (viel, guter)
- ☐ Kein Druck und Stress
- ☐ Flüssigkeit (viel)
- ☐ Energydrinks
- ☐ Andere

1.5.1.1 Andere: *

Beantworten Sie diese Frage nur, wenn folgende Bedingungen erfüllt sind:
Antwort war bei Frage '57 [NahrungsmTop1]' (1. Welche Nahrungsmittel denkst Du fördern Deine Gesundheit am meisten? 1.5.1 Das ist mein TOP 1 (wichtigster) Faktor für meine Gesundheit Anmerkung: Nur 1 Antwort möglich!)

Bitte geben Sie Ihre Antwort hier ein:

☐

1. Welche Nahrungsmittel denkst Du fördern Deine Gesundheit am meisten?
1.5.2 Das ist mein **TOP 2 Faktor** für meine Gesundheit
Anmerkung:
Nur 1 Antwort möglich! *

Bitte wählen Sie alle zutreffenden Antworten aus:

- ☐ Fleisch und Wurst, z. B. Schnitzel, Streichwurst, Fleischkäse/Leberkäse, Frankfurter Würstchen, Salami, Schinken, etc.
- ☐ Fisch und sog. „Meeresfrüchte“, z. B. Muscheln, Scampi, Fischöl, etc.
- ☐ Milch und Milchprodukte, z. B. Käse, Butter, Joghurt, Topfen/Quark, Sahne, etc.
- ☐ Eier
- ☐ Obst
- ☐ Gemüse
- ☐ Getreideprodukte, z. B. Brot, Nudeln, Reis, Müsli, etc.
- ☐ Kartoffeln und Hülsenfrüchte, z. B. Bohnen, Erbsen, etc.
- ☐ Nahrungsergänzungsmittel bzw. Supplemente, für z. B. Vitamine, Mineralstoffe und Spurenelemente, Protein, Ballaststoffe, etc. in Form von Tabletten/Pillen und Pulver
- ☐ Schlaf (viel, guter)
- ☐ Kein Druck und Stress
- ☐ Flüssigkeit (viel)
- ☐ Energydrinks
- ☐ Andere

1.5.2.1 Andere: *

Beantworten Sie diese Frage nur, wenn folgende Bedingungen erfüllt sind:
Antwort war bei Frage '59 [NahrungsmTop2]' (1. Welche Nahrungsmittel denkst Du fördern Deine Gesundheit am meisten? 1.5.2 Das ist mein TOP 2 Faktor für meine Gesundheit Anmerkung: Nur 1 Antwort möglich!)

Bitte geben Sie Ihre Antwort hier ein:

[ ]

1. Welche Nahrungsmittel denkst Du fördern Deine Gesundheit am meisten?
1.5.3 Das ist mein **TOP 3 Faktor** für meine Gesundheit
Anmerkung:
Nur 1 Antwort möglich! *

Bitte wählen Sie alle zutreffenden Antworten aus:

- ☐ Fleisch und Wurst, z. B. Schnitzel, Streichwurst, Fleischkäse/Leberkäse, Frankfurter Würstchen, Salami, Schinken, etc.
- ☐ Fisch und sog. „Meeresfrüchte“, z. B. Muscheln, Scampi, Fischöl, etc.
- ☐ Milch und Milchprodukte, z. B. Käse, Butter, Joghurt, Topfen/Quark, Sahne, etc.
- ☐ Eier
- ☐ Obst
- ☐ Gemüse
- ☐ Getreideprodukte, z. B. Brot, Nudeln, Reis, Müsli, etc.
- ☐ Kartoffeln und Hülsenfrüchte, z. B. Bohnen, Erbsen, etc.
- ☐ Nahrungsergänzungsmittel bzw. Supplemente, für z. B. Vitamine, Mineralstoffe und Spurenelemente, Protein, Ballaststoffe, etc. in Form von Tabletten/Pillen und Pulver
- ☐ Schlaf (viel, guter)
- ☐ Kein Druck und Stress
- ☐ Flüssigkeit (viel)
- ☐ Energydrinks
- ☐ Andere

1.5.3.1 Andere: *

Beantworten Sie diese Frage nur, wenn folgende Bedingungen erfüllt sind:
Antwort war bei Frage '61 [NahrungsmTop3]' (1. Welche Nahrungsmittel denkst Du fördern Deine Gesundheit am meisten? 1.5.3 Das ist mein TOP 3 Faktor für meine Gesundheit Anmerkung: Nur 1 Antwort möglich!)

Bitte geben Sie Ihre Antwort hier ein:

2. Wie denkst Du ist Bewegung & Sport gesund und fördert Deine Gesundheit?

2.1 Häufigkeit: Wie oft denkst Du ist Bewegung & Sport gesund und fördert Deine Gesundheit? *

Bitte wählen Sie nur eine der folgenden Antworten aus:

- ◯ nie
- ◯ ab und zu, gelegentlich
- ◯ regelmäßig

2.1.1 Häufigkeit: ab und zu, gelegentlich, z. B. *

Beantworten Sie diese Frage nur, wenn folgende Bedingungen erfüllt sind:
Antwort war 'ab und zu, gelegentlich' bei Frage '63 [BUSHaeufigkeit]' (2. Wie denkst Du ist Bewegung & Sport gesund und fördert Deine Gesundheit? 2.1 Häufigkeit: Wie oft denkst Du ist Bewegung & Sport gesund und fördert Deine Gesundheit?)

Bitte wählen Sie nur eine der folgenden Antworten aus:

- ◯ 1mal pro Monat
- ◯ 2mal pro Monat
- ◯ 1mal pro Woche
- ◯ Sonstiges ____________

2.1.2 Häufigkeit: regelmäßig *

Beantworten Sie diese Frage nur, wenn folgende Bedingungen erfüllt sind:
Antwort war 'regelmäßig' bei Frage '63 [BUSHaeufigkeit]' (2. Wie denkst Du ist Bewegung & Sport gesund und fördert Deine Gesundheit? 2.1 Häufigkeit: Wie oft denkst Du ist Bewegung & Sport gesund und fördert Deine Gesundheit?)

Bitte wählen Sie nur eine der folgenden Antworten aus:

- ○ 5mal pro Woche
- ○ 4mal pro Woche
- ○ 3mal pro Woche
- ○ 2mal pro Woche
- ○ täglich
- ○ Sonstiges ________

2. Wie denkst Du ist Bewegung & Sport gesund und fördert Deine Gesundheit?
2.2 Dauer: Wie lange denkst Du soll Bewegung & Sport dauern, damit es gesund und förderlich für Deine Gesundheit ist? *

Bitte wählen Sie nur eine der folgenden Antworten aus:

- ○ weniger als 20 min/Tag reichen aus
- ○ mindestens 20 min/Tag
- ○ etwa 1 h/Tag
- ○ mindestens 1 h/Tag
- ○ Sonstiges ________

2. Wie denkst Du ist Bewegung & Sport gesund und fördert Deine Gesundheit?
2.3 Intensität: Wie intensiv denkst Du soll Bewegung & Sport sein, damit es gesund und förderlich für Deine Gesundheit ist? *

Bitte wählen Sie nur eine der folgenden Antworten aus:

- ◯ locker, niedrige Intensität
- ◯ gefordert, nicht zu locker/nicht zu anstrengend, mittlere Intensität
- ◯ anstrengend, hohe Intensität
- ◯ Sonstiges ______

3. Was denkst Du ist der wichtigste (Haupt-)Faktor für Deine Gesundheit? *

Bitte wählen Sie nur eine der folgenden Antworten aus:

- ◯ Bewegung & Sport alleine
- ◯ Ernährung alleine
- ◯ abwechselnd achten auf: ab und zu Bewegung & Sport, ab und zu gesunde Ernährung
- ◯ Bewegung & Sport immer kombiniert mit Ernährung
- ◯ Medikamente & Therapie (vom Arzt verschrieben)
- ◯ Sonstiges ______

4. Leidest Du an Lebensmittel-/Nahrungsmittel-Allergien oder -Intoleranzen? *

Bitte wählen Sie nur eine der folgenden Antworten aus:

- ◯ Ja
- ◯ Nein

4.1 Welche Lebensmittel-/Nahrungsmittel-Allergien oder -Intoleranzen hast Du?

Beantworten Sie diese Frage nur, wenn folgende Bedingungen erfüllt sind:
Antwort war 'Ja' bei Frage '69 [LebensmAllergien]' (4. Leidest Du an Lebensmittel-/Nahrungsmittel-Allergien oder -Intoleranzen?)

Bitte geben Sie Ihre Antwort hier ein:

TEIL E – SONSTIGES zum Abschluss

1. Isst Du folgende Lebensmittel? *Anmerkung: Mehrfachnennung möglich!* *

Bitte wählen Sie alle zutreffenden Antworten aus:

- [ ] Obst
- [ ] Gemüse
- [ ] Getreideprodukte, z. B. Brot, Nudeln, Reis, Müsli, Cornflakes, Seitan, etc.
- [ ] Kartoffeln und Hülsenfrüchte, z. B. Bohnen, Erbsen, Linsen, Tofu, Pommes Frites, etc.
- [ ] Fleisch und Wurst, z. B. Schnitzel, Streichwurst, Fleischkäse/Leberkäse, Frankfurter Würstchen, Salami, Schinken, etc.
- [ ] Fisch und sog. „Meeresfrüchte“, z. B. Muscheln, Scampi, Fischöl, etc.
- [ ] Milch und Milchprodukte, z. B. Käse, Butter, Joghurt, Topfen/Quark, Sahne, etc.
- [ ] Eier
- [ ] Honig

2. Diese Freizeit-Aktivitäten sind mir wichtig ...
2.1 Das ist meine **TOP 1 (liebste) Freizeit-Aktivität**
Anmerkung:

Nur 1 Antwort möglich! *

Bitte wählen Sie alle zutreffenden Antworten aus:

- ☐ Fernsehen/Video schauen
- ☐ Musik (z. B. hören, singen, Instrument spielen, Lyrics schreiben)
- ☐ Computer spielen/Internet surfen
- ☐ Sport treiben
- ☐ Lesen/Schreiben
- ☐ Freunde treffen (z. B. Bummeln, Chillen, Shoppen gehen, etc.)
- ☐ Basteln, Malen, Handarbeiten, etc.
- ☐ andere

2.1.1 Andere: *

Beantworten Sie diese Frage nur, wenn folgende Bedingungen erfüllt sind:
Antwort war bei Frage '72 [FreizeitAktivitaet1]' (2. Diese Freizeit-Aktivitäten sind mir wichtig ... 2.1 Das ist meine TOP 1 (liebste) Freizeit-Aktivität Anmerkung: Nur 1 Antwort möglich!)

Bitte geben Sie Ihre Antwort hier ein:

2. Diese Freizeit-Aktivitäten sind mir wichtig ...
2.2 Das ist meine **TOP 2 Freizeit-Aktivität**
Anmerkung:
Nur 1 Antwort möglich! *

Bitte wählen Sie alle zutreffenden Antworten aus:

- ☐ Fernsehen/Video schauen
- ☐ Musik (z. B. hören, singen, Instrument spielen, Lyrics schreiben)
- ☐ Computer spielen/Internet surfen
- ☐ Sport treiben
- ☐ Lesen/Schreiben
- ☐ Freunde treffen (z. B. Bummeln, Chillen, Shoppen gehen, etc.)
- ☐ Basteln, Malen, Handarbeiten, etc.
- ☐ andere

2.2.1 Andere: *

Beantworten Sie diese Frage nur, wenn folgende Bedingungen erfüllt sind:
Antwort war bei Frage '74 [FreizeitAktivitaet2]' (2. Diese Freizeit-Aktivitäten sind mir wichtig ... 2.2 Das ist meine TOP 2 Freizeit-Aktivität Anmerkung: Nur 1 Antwort möglich!)

Bitte geben Sie Ihre Antwort hier ein:

2. Diese Freizeit-Aktivitäten sind mir wichtig ...
2.3 Das ist meine **TOP 3 Freizeit-Aktivität**
Anmerkung:
Nur 1 Antwort möglich! *

Bitte wählen Sie alle zutreffenden Antworten aus:

- ☐ Fernsehen/Video schauen
- ☐ Musik (z. B. hören, singen, Instrument spielen, Lyrics schreiben)
- ☐ Computer spielen/Internet surfen
- ☐ Sport treiben
- ☐ Lesen/Schreiben
- ☐ Freunde treffen (z. B. Bummeln, Chillen, Shoppen gehen, etc.)
- ☐ Basteln, Malen, Handarbeiten, etc.
- ☐ andere

2.3.1 Andere: *

Beantworten Sie diese Frage nur, wenn folgende Bedingungen erfüllt sind:
Antwort war bei Frage '76 [FreizeitAktivitaet3]' (2. Diese Freizeit-Aktivitäten sind mir wichtig ... 2.3 Das ist meine TOP 3 Freizeit-Aktivität Anmerkung: Nur 1 Antwort möglich!)

Bitte geben Sie Ihre Antwort hier ein:

3. Rauchst Du Zigaretten? *

Bitte wählen Sie nur eine der folgenden Antworten aus:

- ◯ Ja
- ◯ Nein

3.1 Wie oft rauchst Du? *

Beantworten Sie diese Frage nur, wenn folgende Bedingungen erfüllt sind:
Antwort war 'Ja' bei Frage '78 [Rauchen]' (3. Rauchst Du Zigaretten?)

Bitte wählen Sie nur eine der folgenden Antworten aus:

- ◯ täglich
- ◯ regelmäßig, aber nicht täglich
- ◯ gelegentlich

4. Lifestyle von Jugendlichen: Was findest Du cool hinsichtlich (Deinem) Lifestyle?
Anmerkung:
Mehrfachnennung möglich! *

Bitte wählen Sie alle zutreffenden Antworten aus:

- ☐ Generell Sport treiben
- ☐ Lifestyle einer bestimmten Sportart, z. B. Snowboarden (Kleidung, Philosophie, etc.)
- ☐ Alkohol trinken
- ☐ Zigaretten rauchen
- ☐ Fleisch essen
- ☐ Vegetarisch essen
- ☐ Vegetarischer Lifestyle (Kleidung, Philosophie, etc.)
- ☐ Vegan essen
- ☐ Veganer Lifestyle (Kleidung, Philosophie, etc.)
- ☐ Sonstiges: ______________________

4.1 Was findest Du **am coolsten (TOP 1)** hinsichtlich (Deinem) Lifestyle?
Anmerkung:
Nur 1 Antwort möglich! *

Bitte wählen Sie alle zutreffenden Antworten aus:

- ☐ Generell Sport treiben
- ☐ Lifestyle einer bestimmten Sportart, z. B. Snowboarden (Kleidung, Philosophie, etc.)
- ☐ Alkohol trinken
- ☐ Zigaretten rauchen
- ☐ Fleisch essen
- ☐ Vegetarisch essen
- ☐ Vegetarischer Lifestyle (Kleidung, Philosophie, etc.)
- ☐ Vegan essen
- ☐ Veganer Lifestyle (Kleidung, Philosophie, etc.)
- ☐ Sonstiges

4.2 Was findest Du **cool (TOP 2)** hinsichtlich (Deinem) Lifestyle?
Anmerkung:
Nur 1 Antwort möglich! *

Bitte wählen Sie alle zutreffenden Antworten aus:

- ☐ Generell Sport treiben
- ☐ Lifestyle einer bestimmten Sportart, z. B. Snowboarden (Kleidung, Philosophie, etc.)
- ☐ Alkohol trinken
- ☐ Zigaretten rauchen
- ☐ Fleisch essen
- ☐ Vegetarisch essen
- ☐ Vegetarischer Lifestyle (Kleidung, Philosophie, etc.)
- ☐ Vegan essen
- ☐ Veganer Lifestyle (Kleidung, Philosophie, etc.)
- ☐ Sonstiges

4.3 Was findest Du **cool (TOP 3)** hinsichtlich (Deinem) Lifestyle?
Anmerkung:
Nur 1 Antwort möglich! *

Bitte wählen Sie alle zutreffenden Antworten aus:

- ☐ Generell Sport treiben
- ☐ Lifestyle einer bestimmten Sportart, z. B. Snowboarden (Kleidung, Philosophie, etc.)
- ☐ Alkohol trinken
- ☐ Zigaretten rauchen
- ☐ Fleisch essen
- ☐ Vegetarisch essen
- ☐ Vegetarischer Lifestyle (Kleidung, Philosophie, etc.)
- ☐ Vegan essen
- ☐ Veganer Lifestyle (Kleidung, Philosophie, etc.)
- ☐ Sonstiges

5. Trinkst Du Alkohol? *

Bitte wählen Sie nur eine der folgenden Antworten aus:

- ◯ Ja
- ◯ Nein

5.1 Wie oft trinkst Du Alkohol? *

Beantworten Sie diese Frage nur, wenn folgende Bedingungen erfüllt sind:
Antwort war 'Ja' bei Frage '84 [Alkohol]' (5. Trinkst Du Alkohol?)

Bitte wählen Sie nur eine der folgenden Antworten aus:

- ◯ täglich
- ◯ regelmäßig, aber nicht täglich
- ◯ gelegentlich

Vielen Dank für Deine Teilnahme an der Schulstudie
‚From Science 2 School: Nachhaltig gesund – bewegt & veggie‘.

Dein Fragebogen wurde erfolgreich abgesendet.

Übermittlung Ihres ausgefüllten Fragebogens:
Vielen Dank für die Beantwortung des Fragebogens.
