# Supplementary material for: Study protocol of “From Science 2 School”—prevalence of sports and physical exercise linked to omnivorous, vegetarian and vegan, diets among Austrian secondary schools
Source: Front Sports Act Living. 2022 Sep 28;4:967915. doi: 10.3389/fspor.2022.967915 (PMC9554638; doi:10.3389/fspor.2022.967915)
Supplement: Supplementary file 2 [file Data_Sheet_2.pdf]

From Science 2 School - Fragebogen Lehrer_innen

Herzlich willkommen bei der Online-Umfrage zur Schulstudie

‚From Science 2 School: Nachhaltig gesund – bewegt & veggie‘!

In dieser Umfrage sind 86 Fragen enthalten.

TEIL A – Zur PERSON

1. Ihre Nationalität? *

Bitte wählen Sie nur eine der folgenden Antworten aus:

- ◯ Österreich
- ◯ andere

1.1 Ihre Nationalität: Andere? *

Beantworten Sie diese Frage nur, wenn folgende Bedingungen erfüllt sind:
Antwort war 'andere' bei Frage '1 [Nationalitaet1]' (1. Ihre Nationalität?)

Bitte wählen Sie nur eine der folgenden Antworten aus:

- ◯ Afghanistan
- ◯ Ägypten
- ◯ Albanien
- ◯ Algerien
- ◯ Andorra
- ◯ Angola
- ◯ Antigua und Barbuda
- ◯ Äquatorialguinea
- ◯ Argentinien
- ◯ Armenien
- ◯ Aserbaidschan
- ◯ Äthiopien
- ◯ Australien
- ◯ Bahamas
- ◯ Bahrain
- ◯ Bangladesch
- ◯ Barbados
- ◯ Belgien
- ◯ Belize
- ◯ Benin
- ◯ Bhutan
- ◯ Bolivien
- ◯ Bosnien und Herzegowina
- ◯ Botsuana
- ◯ Brasilien
- ◯ Brunei Darussalam
- ◯ Bulgarien
- ◯ Burkina Faso
- ◯ Burundi
- ◯ Chile

- ◯ China
- ◯ Costa Rica
- ◯ Côte d'Ivoire
- ◯ Dänemark
- ◯ Demokratische Republik Kongo
- ◯ Deutschland
- ◯ Dominica
- ◯ Dominikanische Republik
- ◯ Dschibuti
- ◯ Ecuador
- ◯ El Salvador
- ◯ Eritrea
- ◯ Estland
- ◯ Eswatini
- ◯ Fidschi
- ◯ Finnland
- ◯ Föderierte Staaten Mikronesien
- ◯ Frankreich
- ◯ Gabun
- ◯ Gambia
- ◯ Georgien
- ◯ Ghana
- ◯ Grenada
- ◯ Griechenland
- ◯ Guatemala
- ◯ Guinea
- ◯ Guinea-Bissau
- ◯ Guyana
- ◯ Haiti
- ◯ Honduras
- ◯ Indien
- ◯ Indonesien
- ◯ Irak
- ◯ Iran
- ◯ Irland

- ◯ Island
- ◯ Israel
- ◯ Italien
- ◯ Jamaika
- ◯ Japan
- ◯ Jemen
- ◯ Jordanien
- ◯ Kambodscha
- ◯ Kamerun
- ◯ Kanada
- ◯ Kap Verde
- ◯ Kasachstan
- ◯ Katar
- ◯ Kenia
- ◯ Kirgisistan
- ◯ Kiribati
- ◯ Kolumbien
- ◯ Komoren
- ◯ Demokratische Volksrepublik Korea
- ◯ Republik Korea
- ◯ Kroatien
- ◯ Kuba
- ◯ Kuwait
- ◯ Laos
- ◯ Lesotho
- ◯ Lettland
- ◯ Libanon
- ◯ Liberia
- ◯ Libyen
- ◯ Liechtenstein
- ◯ Litauen
- ◯ Luxemburg
- ◯ Madagaskar
- ◯ Malawi
- ◯ Malaysia

- Malediven
- Mali
- Malta
- Marokko
- Marshallinsel
- Mauretanien
- Mauritius
- Mazedonien
- Mexiko
- Moldau
- Monaco
- Mongolei
- Montenegro
- Mosambik
- Myanmar
- Namibia
- Nauru
- Nepal
- Neuseeland
- Nicaragua
- Niederlande
- Niger
- Nigeria
- Norwegen
- Oman
- Osttimor
- Pakistan
- Palau
- Panama
- Papua-Neuguinea
- Paraguay
- Peru
- Philippinen
- Polen
- Portugal

- Republik Kongo
- Ruanda
- Rumänien
- Russische Föderation
- Saint Kitts und Nevis
- Saint Lucia
- Salomonen
- Sambia
- Samoa
- San Marino
- São Tomé und Príncipe
- Saudi-Arabien
- Schweden
- Schweiz
- Senegal
- Serbien
- Seychellen
- Sierra Leone
- Simbabwe
- Singapur
- Slowakei
- Slowenien
- Somalia
- Spanien
- Sri Lanka
- St. Vincent und die Grenadinen
- Südafrika
- Sudan
- Südsudan
- Suriname
- Syrien
- Tadschikistan
- Tansania
- Thailand
- Togo

- Tonga
- Trinidad und Tobago
- Tschad
- Tschechische Republik
- Tunesien
- Türkei
- Turkmenistan
- Tuvalu
- Uganda
- Ukraine
- Ungarn
- Uruguay
- Usbekistan
- Vanuatu
- Vatikanstaat
- Venezuela
- Vereinigte Arabische Emirate
- Vereinigte Staaten von Amerika
- Vereinigtes Königreich
- Vietnam
- Weißrussland
- Zentralafrikanische Republik
- Zypern

2. Ihr Wohnort? *

Bitte wählen Sie nur eine der folgenden Antworten aus:

- Stadt/städtische Region
- Land/ländliche Region

2.1 Ihr Bundesland? *

Bitte wählen Sie nur eine der folgenden Antworten aus:

- ◯ Burgenland
- ◯ Kärnten
- ◯ Niederösterreich
- ◯ Oberösterreich
- ◯ Salzburg
- ◯ Steiermark
- ◯ Tirol
- ◯ Vorarlberg
- ◯ Wien

3. Ihr Geschlecht? *

Bitte wählen Sie nur eine der folgenden Antworten aus:

- ◯ männlich
- ◯ weiblich

4. Ihr Körpergewicht (kg)?

Beispiel:
40,3 kg oder 37,80 kg
51 kg oder 85,0 kg

Anmerkung:
Falls Sie aktuell schwanger sind, geben Sie bitte an, welches Gewicht Sie VOR Ihrer Schwangerschaft hatten. Nur Zahlen-Eingabe möglich (zwischen 20,0 kg und 160,0 kg)! *

❗ Ihre Antwort muss zwischen 20 und 160 liegen.

Bitte geben Sie Ihre Antwort hier ein:

[ ]

kg

Nur Zahlen-Eingabe möglich (zwischen 20,0 kg und 160,0 kg)!

5. Ihre Körpergröße (m)? *

Bitte wählen Sie nur eine der folgenden Antworten aus:

- ◯ 1,00
- ◯ 1,01
- ◯ 1,02
- ◯ 1,03
- ◯ 1,04
- ◯ 1,05
- ◯ 1,06
- ◯ 1,07
- ◯ 1,08
- ◯ 1,09
- ◯ 1,10
- ◯ 1,11
- ◯ 1,12
- ◯ 1,13
- ◯ 1,14
- ◯ 1,15
- ◯ 1,16
- ◯ 1,17
- ◯ 1,18
- ◯ 1,19
- ◯ 1,20
- ◯ 1,21
- ◯ 1,22
- ◯ 1,23
- ◯ 1,24
- ◯ 1,25
- ◯ 1,26
- ◯ 1,27
- ◯ 1,28
- ◯ 1,29
- ◯ 1,30

- ◯ 1,31
- ◯ 1,32
- ◯ 1,33
- ◯ 1,34
- ◯ 1,35
- ◯ 1,36
- ◯ 1,37
- ◯ 1,38
- ◯ 1,39
- ◯ 1,40
- ◯ 1,41
- ◯ 1,42
- ◯ 1,43
- ◯ 1,44
- ◯ 1,45
- ◯ 1,46
- ◯ 1,47
- ◯ 1,48
- ◯ 1,49
- ◯ 1,50
- ◯ 1,51
- ◯ 1,52
- ◯ 1,53
- ◯ 1,54
- ◯ 1,55
- ◯ 1,56
- ◯ 1,57
- ◯ 1,58
- ◯ 1,59
- ◯ 1,60
- ◯ 1,61
- ◯ 1,62
- ◯ 1,63
- ◯ 1,64
- ◯ 1,65

- ◯ 1,66
- ◯ 1,67
- ◯ 1,68
- ◯ 1,69
- ◯ 1,70
- ◯ 1,71
- ◯ 1,72
- ◯ 1,73
- ◯ 1,74
- ◯ 1,75
- ◯ 1,76
- ◯ 1,77
- ◯ 1,78
- ◯ 1,79
- ◯ 1,80
- ◯ 1,81
- ◯ 1,82
- ◯ 1,83
- ◯ 1,84
- ◯ 1,85
- ◯ 1,86
- ◯ 1,87
- ◯ 1,88
- ◯ 1,89
- ◯ 1,90
- ◯ 1,91
- ◯ 1,92
- ◯ 1,93
- ◯ 1,94
- ◯ 1,95
- ◯ 1,96
- ◯ 1,97
- ◯ 1,98
- ◯ 1,99
- ◯ 2,00

- ◯ 2,01
- ◯ 2,02
- ◯ 2,03
- ◯ 2,04
- ◯ 2,05
- ◯ 2,06
- ◯ 2,07
- ◯ 2,08
- ◯ 2,09
- ◯ 2,10
- ◯ 2,11
- ◯ 2,12
- ◯ 2,13
- ◯ 2,14
- ◯ 2,15
- ◯ 2,16
- ◯ 2,17
- ◯ 2,18
- ◯ 2,19
- ◯ 2,20

6. Ihr Alter? *

Bitte wählen Sie nur eine der folgenden Antworten aus:

- ◯ 20
- ◯ 21
- ◯ 22
- ◯ 23
- ◯ 24
- ◯ 25
- ◯ 26
- ◯ 27
- ◯ 28
- ◯ 29
- ◯ 30
- ◯ 31
- ◯ 32
- ◯ 33
- ◯ 34
- ◯ 35
- ◯ 36
- ◯ 37
- ◯ 38
- ◯ 39
- ◯ 40
- ◯ 41
- ◯ 42
- ◯ 43
- ◯ 44
- ◯ 45
- ◯ 46
- ◯ 47
- ◯ 48
- ◯ 49
- ◯ 50

- ◯ 51
- ◯ 52
- ◯ 53
- ◯ 54
- ◯ 55
- ◯ 56
- ◯ 57
- ◯ 58
- ◯ 59
- ◯ 60
- ◯ 61
- ◯ 62
- ◯ 63
- ◯ 64
- ◯ 65

7. Dauer Ihrer Berufsausübung/Dienstalter: Wieviele Dienstjahre unterrichten Sie bereits? *

Bitte wählen Sie nur eine der folgenden Antworten aus:

- ◯ 1
- ◯ 2
- ◯ 3
- ◯ 4
- ◯ 5
- ◯ 6
- ◯ 7
- ◯ 8
- ◯ 9
- ◯ 10
- ◯ 11
- ◯ 12
- ◯ 13
- ◯ 14
- ◯ 15
- ◯ 16
- ◯ 17
- ◯ 18
- ◯ 19
- ◯ 20
- ◯ 21
- ◯ 22
- ◯ 23
- ◯ 24
- ◯ 25
- ◯ 26
- ◯ 27
- ◯ 28
- ◯ 29
- ◯ 30

- 31
- 32
- 33
- 34
- 35
- 36
- 37
- 38
- 39
- 40
- 41
- 42
- 43
- 44
- 45
- 46

8. Ihr Beschäftigungsausmaß? *

Bitte wählen Sie nur eine der folgenden Antworten aus:

- 100 % (Vollzeit)
- Teilzeit

8.1 Ihr Beschäftigungsausmaß Teilzeit in Prozent (%)? *

Beantworten Sie diese Frage nur, wenn folgende Bedingungen erfüllt sind:
Antwort war 'Teilzeit' bei Frage '10 [Beschaeftigungsausm]' (8. Ihr Beschäftigungsausmaß?)

Bitte wählen Sie nur eine der folgenden Antworten aus:

- ○ 5
- ○ 10
- ○ 15
- ○ 20
- ○ 25
- ○ 30
- ○ 35
- ○ 40
- ○ 45
- ○ 50
- ○ 55
- ○ 60
- ○ 65
- ○ 70
- ○ 75
- ○ 80
- ○ 85
- ○ 90
- ○ 95
- ○ 100

9. Ihre wöchentliche Arbeitszeit im Durchschnitt? *

Bitte wählen Sie nur eine der folgenden Antworten aus:

- ◯ bis 30 Stunden/Woche
- ◯ 31 – 40 Stunden/Woche
- ◯ 41 – 50 Stunden/Woche
- ◯ > 50 Stunden/Woche (Selbsteingabe):

10. In welcher Schulstufe unterrichten Sie? *

Bitte wählen Sie nur eine der folgenden Antworten aus:

- ◯ Unterstufe (=Sekundarstufe 1, Schulstufe 5 – 8)
- ◯ Oberstufe (=Sekundarstufe 2, Schulstufe 9 – 13)
- ◯ Beides: Unter- & Oberstufe (=Sekundarstufe 1 & 2, Schulstufe 5 – 13)

11. An welcher Schule (Schultyp) unterrichten Sie? *

Beantworten Sie diese Frage nur, wenn folgende Bedingungen erfüllt sind:
Antwort war 'Unterstufe (=Sekundarstufe 1, Schulstufe 5 – 8)' bei Frage '13 [Schulstufe]' (10. In welcher Schulstufe unterrichten Sie?)

Bitte wählen Sie alle zutreffenden Antworten aus:

- ☐ MS – Mittelschule (vorher: NMS – Neue Mittelschule)
- ☐ AHS – Allgemeinbildende Höhere Schule (z. B. Gymnasium Unterstufe)
- ☐ andere:

11. An welcher Schule (Schultyp) unterrichten Sie? *

Beantworten Sie diese Frage nur, wenn folgende Bedingungen erfüllt sind:
Antwort war 'Oberstufe (=Sekundarstufe 2, Schulstufe 9 – 13)' bei Frage '13 [Schulstufe]' (10. In welcher Schulstufe unterrichten Sie?)

Bitte wählen Sie alle zutreffenden Antworten aus:

- ☐ Polytechnische Schule
- ☐ BMS – Berufsbildende Mittlere Schulen (Fachschulen wie z. B. Handelsschule (HAS) bzw. 3- oder 4-jährig an HBLA oder HTL, etc.)
- ☐ AHS – Allgemeinbildende Höhere Schule (z. B. Gymnasium Oberstufe)
- ☐ BHS – Berufsbildende Höhere Schulen mit Matura (z. B. 5-jährig an HAK, HBLA, HTL usw.)
- ☐ Berufsschule
- ☐ andere: ______

11. Beides: Unter- & Oberstufe (=Sekundarstufe 1 & 2) *

Beantworten Sie diese Frage nur, wenn folgende Bedingungen erfüllt sind:
Antwort war 'Beides: Unter- & Oberstufe (=Sekundarstufe 1 & 2, Schulstufe 5 – 13)' bei Frage '13 [Schulstufe]' (10. In welcher Schulstufe unterrichten Sie?)

Bitte wählen Sie alle zutreffenden Antworten aus:

- ☐ MS – Mittelschule (vorher: NMS – Neue Mittelschule)
- ☐ Polytechnische Schule
- ☐ AHS Unterstufe – Allgemeinbildende Höhere Schule
- ☐ AHS Oberstufe – Allgemeinbildende Höhere Schule
- ☐ BMS – Berufsbildende Mittlere Schulen (Fachschulen wie z. B. Handelsschule (HAS) bzw. 3- oder 4-jährig an HBLA oder HTL, etc.)
- ☐ BHS – Berufsbildende Höhere Schulen mit Matura (z. B. 5-jährig an HAK, HBLA, HTL usw.)
- ☐ Berufsschule
- ☐ andere: ______

12. Ihr Stimmungs-Barometer *

Bitte wählen Sie die zutreffende Antwort für jeden Punkt aus:

	Ja, stimmt genau	Stimmt eher	Stimmt weniger	Nein, stimmt nicht
**Ich betreibe gerne Bewegung & Sport, weil es mir Spaß macht und gesund ist.**	○	○	○	○
**Ich interessiere mich für meine Gesundheit und informiere mich.**	○	○	○	○
**Ich interessiere mich für Ernährung, was gesund bzw. was ungesund ist.**	○	○	○	○

TEIL B – BEWEGUNG & SPORT

Definition „Sport" in Anlehnung an den LBIHPR Forschungsbericht (Hofmann et al. 2012) – Sie kommen bei Ihrer Freizeitaktivität außer Atem oder ins Schwitzen, z. B. Wandern oder Wassergymnastik, uvm.

1. Betreiben Sie Sport in Ihrer Freizeit? *

Bitte wählen Sie nur eine der folgenden Antworten aus:

- ○ Ja
- ○ Nein

1.1 Warum betreiben Sie keinen Sport in Ihrer Freizeit?

Beantworten Sie diese Frage nur, wenn folgende Bedingungen erfüllt sind:
Antwort war 'Nein' bei Frage '18 [BetreibenSieSport]' (1. Betreiben Sie Sport in Ihrer Freizeit?)

Bitte geben Sie Ihre Antwort hier ein:

2. Das ist Ihr Beweggrund für Bewegung & Sport, das ist Ihnen wichtig ...
2.1 Das ist Ihr **TOP 1 (wichtigster) Beweggrund** für Bewegung & Sport
Anmerkung:
Nur 1 Antwort möglich! *

Beantworten Sie diese Frage nur, wenn folgende Bedingungen erfüllt sind:
Antwort war 'Ja' bei Frage '18 [BetreibenSieSport]' (1. Betreiben Sie Sport in Ihrer Freizeit?)

Bitte wählen Sie alle zutreffenden Antworten aus:

- ☐ Eigenes Interesse: macht mir Freude/Spaß
- ☐ Eltern/Geschwister/Familie (inkl. Haustier, z. B. Hund)
- ☐ Kinder
- ☐ Ehepartner/Lebensgefährte
- ☐ Freunde/Freund_in
- ☐ Lehrerkolleg_innen (Schule)
- ☐ Sportverein
- ☐ Vorbilder (z. B. Sportler, Promis in Medien)
- ☐ Gesundheit/Wohlbefinden
- ☐ Druck und Stress abbauen
- ☐ Sich mit anderen messen (z. B. Wettkampf)
- ☐ Anerkennung
- ☐ Nervenkitzel/Abenteuer/Erlebnis (Adrenalinkick)
- ☐ Fitness (z. B. kräftig, ausdauernd, wendig)
- ☐ Aussehen/Ästhetischer Körper (gute Figur z. B. schlank, schön, definierte Muskeln)
- ☐ Entspannung/Ausgleich
- ☐ Lifestyle (spezielles Outfit, z. B. Tanz, Kraftsport, etc.)
- ☐ Kein bestimmter Grund
- ☐ Andere

2.1.1 Andere: *

Beantworten Sie diese Frage nur, wenn folgende Bedingungen erfüllt sind:
Antwort war 'Andere' bei Frage '20 [TOP1Beweggr1]' (2. Das ist Ihr Beweggrund für Bewegung & Sport, das ist Ihnen wichtig ... 2.1 Das ist Ihr TOP 1 (wichtigster) Beweggrund für Bewegung & Sport Anmerkung: Nur 1 Antwort möglich!)

Bitte geben Sie Ihre Antwort hier ein:

2. Das ist Ihr Beweggrund für Bewegung & Sport, das ist Ihnen wichtig ...

2.2 Das ist Ihr **TOP 2 Beweggrund** für Bewegung & Sport

Anmerkung:

Nur 1 Antwort möglich! *

Beantworten Sie diese Frage nur, wenn folgende Bedingungen erfüllt sind:
Antwort war 'Ja' bei Frage '18 [BetreibenSieSport]' (1. Betreiben Sie Sport in Ihrer Freizeit?)

Bitte wählen Sie alle zutreffenden Antworten aus:

- ☐ Eigenes Interesse: macht mir Freude/Spaß
- ☐ Eltern/Geschwister/Familie (inkl. Haustier, z. B. Hund)
- ☐ Kinder
- ☐ Ehepartner/Lebensgefährte
- ☐ Freunde/Freund_in
- ☐ Lehrerkolleg_innen (Schule)
- ☐ Sportverein
- ☐ Vorbilder (z. B. Sportler, Promis in Medien)
- ☐ Gesundheit/Wohlbefinden
- ☐ Leistung verbessern
- ☐ Sich mit anderen messen (z. B. Wettkampf)
- ☐ Anerkennung
- ☐ Nervenkitzel/Abenteuer/Erlebnis (Adrenalinkick)
- ☐ Fitness (z. B. kräftig, ausdauernd, wendig)
- ☐ Aussehen/Ästhetischer Körper (gute Figur z. B. schlank, schön, definierte Muskeln)
- ☐ Entspannung/Ausgleich
- ☐ Druck und Stress abbauen
- ☐ Lifestyle (spezielles Outfit, z. B. Tanz, Kraftsport, etc.)

- ☐ Kein bestimmter Grund
- ☐ Andere

2.2.1 Andere: *

Beantworten Sie diese Frage nur, wenn folgende Bedingungen erfüllt sind:
Antwort war 'Andere' bei Frage '22 [TOP2Beweggr2]' (2. Das ist Ihr Beweggrund für Bewegung & Sport, das ist Ihnen wichtig ... 2.2 Das ist Ihr TOP 2 Beweggrund für Bewegung & Sport Anmerkung: Nur 1 Antwort möglich!)

Bitte geben Sie Ihre Antwort hier ein:

2. Das ist Ihr Beweggrund für Bewegung & Sport, das ist Ihnen wichtig ...

2.3 Das ist Ihr **TOP 3 Beweggrund** für Bewegung & Sport

Anmerkung:

Nur 1 Antwort möglich! *

Beantworten Sie diese Frage nur, wenn folgende Bedingungen erfüllt sind:
Antwort war 'Ja' bei Frage '18 [BetreibenSieSport]' (1. Betreiben Sie Sport in Ihrer Freizeit?)

Bitte wählen Sie alle zutreffenden Antworten aus:

- ☐ Eigenes Interesse: macht mir Freude/Spaß
- ☐ Eltern/Geschwister/Familie (inkl. Haustier, z. B. Hund)
- ☐ Kinder
- ☐ Ehepartner/Lebensgefährte
- ☐ Freunde/Freund_in
- ☐ Lehrerkolleg_innen (Schule)
- ☐ Sportverein
- ☐ Vorbilder (z. B. Sportler, Promis in Medien)
- ☐ Gesundheit/Wohlbefinden
- ☐ Leistung verbessern
- ☐ Sich mit anderen messen (z. B. Wettkampf)
- ☐ Anerkennung
- ☐ Nervenkitzel/Abenteuer/Erlebnis (Adrenalinkick)
- ☐ Fitness (z. B. kräftig, ausdauernd, wendig)
- ☐ Aussehen/Ästhetischer Körper (gute Figur z. B. schlank, schön, definierte Muskeln)
- ☐ Entspannung/Ausgleich
- ☐ Druck und Stress abbauen
- ☐ Lifestyle (spezielles Outfit, z. B. Tanz, Kraftsport, etc.)

- ☐ Kein bestimmter Grund
- ☐ Andere

2.3.1 Andere: *

Beantworten Sie diese Frage nur, wenn folgende Bedingungen erfüllt sind:
Antwort war 'Andere' bei Frage '24 [TOP3Beweggr3]' (2. Das ist Ihr Beweggrund für Bewegung & Sport, das ist Ihnen wichtig ... 2.3 Das ist Ihr TOP 3 Beweggrund für Bewegung & Sport Anmerkung: Nur 1 Antwort möglich!)

Bitte geben Sie Ihre Antwort hier ein:

3. Wie lange schon betreiben Sie Sport als Freizeitaktivität?*

Beantworten Sie diese Frage nur, wenn folgende Bedingungen erfüllt sind:
Antwort war 'Ja' bei Frage '18 [BetreibenSieSport]' (1. Betreiben Sie Sport in Ihrer Freizeit?)

Bitte wählen Sie nur eine der folgenden Antworten aus:

- schon immer, mein Leben lang
- ≤ 0,5 Jahr (1 – 6 Monate)
- 1 Jahr
- 2 Jahre
- 3 – 5 Jahre
- 6 – 9 Jahre
- 10 oder mehr Jahre

4. Mit wem betreiben Sie am häufigsten Sport in Ihrer Freizeit? *

Beantworten Sie diese Frage nur, wenn folgende Bedingungen erfüllt sind:
Antwort war 'Ja' bei Frage '18 [BetreibenSieSport]' (1. Betreiben Sie Sport in Ihrer Freizeit?)

Bitte wählen Sie nur eine der folgenden Antworten aus:

- ◯ Ich alleine
- ◯ Eltern/Geschwister/Familie (inkl. Haustier, z. B. Hund)
- ◯ Kinder
- ◯ Ehepartner/Lebensgefährte
- ◯ Freunde/Freund_in
- ◯ Lehrerkolleg_innen (Schule)
- ◯ Mannschaft/Teamkolleg_innen (z. B. Fußballverein, Tanzgruppe)
- ◯ Trainingspartner_in (wenige, z. B. zu zweit, zu dritt)
- ◯ Trainer_in/Coach

5. Welche Sportart(en) betreiben Sie in Ihrer Freizeit? *Anmerkung: Mehrfachnennung möglich!* *

Beantworten Sie diese Frage nur, wenn folgende Bedingungen erfüllt sind:
Antwort war 'Ja' bei Frage '18 [BetreibenSieSport]' (1. Betreiben Sie Sport in Ihrer Freizeit?)

Bitte wählen Sie alle zutreffenden Antworten aus:

- ☐ Einzelsportart
- ☐ Mannschafts-/Teamsportart

5.1 Welche Einzelsportart(en) betreiben Sie in Ihrer Freizeit?
Anmerkung:
Mehrfachnennung möglich! *

Beantworten Sie diese Frage nur, wenn folgende Bedingungen erfüllt sind:
Antwort war 'Ja' bei Frage '18 [BetreibenSieSport]' (1. Betreiben Sie Sport in Ihrer Freizeit?)
und Antwort war bei Frage '28 [EinzelMannsch]' (5. Welche Sportart(en) betreiben Sie in Ihrer Freizeit? Anmerkung: Mehrfachnennung möglich!)

Bitte wählen Sie alle zutreffenden Antworten aus:

- [ ] Schwimmen
- [ ] Laufen
- [ ] Radfahren
- [ ] Jazz Dance/Hip Hop
- [ ] Skifahren
- [ ] Fitness-/Krafttraining
- [ ] Sonstiges: ____________

5.2 Welche Mannschafts-/Teamsportart(en) betreiben Sie in Ihrer Freizeit?
Anmerkung:
Mehrfachnennung möglich! *

Beantworten Sie diese Frage nur, wenn folgende Bedingungen erfüllt sind:
Antwort war 'Ja' bei Frage '18 [BetreibenSieSport]' (1. Betreiben Sie Sport in Ihrer Freizeit?)
und Antwort war bei Frage '28 [EinzelMannsch]' (5. Welche Sportart(en) betreiben Sie in Ihrer Freizeit? Anmerkung: Mehrfachnennung möglich!)

Bitte wählen Sie alle zutreffenden Antworten aus:

- [ ] Fußball
- [ ] Handball
- [ ] Basketball
- [ ] Volleyball
- [ ] Sonstiges: ____________

5.3 Ihre Haupt-Sportart ist *

Beantworten Sie diese Frage nur, wenn folgende Bedingungen erfüllt sind:
-------- Scenario 1 --------
Antwort war 'Ja' bei Frage '18 [BetreibenSieSport]' (1. Betreiben Sie Sport in Ihrer Freizeit?) *und* Antwort war bei Frage '28 [EinzelMannsch]' (5. Welche Sportart(en) betreiben Sie in Ihrer Freizeit? Anmerkung: Mehrfachnennung möglich!)
-------- oder Scenario 2 --------
Antwort war 'Ja' bei Frage '18 [BetreibenSieSport]' (1. Betreiben Sie Sport in Ihrer Freizeit?) *und* Antwort war bei Frage '28 [EinzelMannsch]' (5. Welche Sportart(en) betreiben Sie in Ihrer Freizeit? Anmerkung: Mehrfachnennung möglich!)

Bitte wählen Sie nur eine der folgenden Antworten aus:

- ◯ Schwimmen
- ◯ Laufen
- ◯ Radfahren
- ◯ Jazz Dance/Hip Hop
- ◯ Skifahren
- ◯ Fitness-/Krafttraining
- ◯ Fußball
- ◯ Handball
- ◯ Basketball
- ◯ Volleyball
- ◯ Sonstiges ____________

6. An wie vielen Tagen pro Woche treiben Sie Sport in Ihrer Freizeit (Hobby, Training, und/oder Wettkampf)? *

Beantworten Sie diese Frage nur, wenn folgende Bedingungen erfüllt sind:
Antwort war 'Ja' bei Frage '18 [BetreibenSieSport]' (1. Betreiben Sie Sport in Ihrer Freizeit?)

Bitte wählen Sie nur eine der folgenden Antworten aus:

- ◯ 1 Tag/Woche
- ◯ 2 Tage/Woche
- ◯ 3 Tage/Woche
- ◯ 4 Tage/Woche
- ◯ 5 Tage/Woche
- ◯ 6 Tage/Woche
- ◯ 7 Tage/Woche

7. Durchschnittliche Dauer Ihrer Sporteinheit (ø Stunden/Sporteinheit)? *

Beantworten Sie diese Frage nur, wenn folgende Bedingungen erfüllt sind:
Antwort war 'Ja' bei Frage '18 [BetreibenSieSport]' (1. Betreiben Sie Sport in Ihrer Freizeit?)

Bitte wählen Sie nur eine der folgenden Antworten aus:

- ◯ 00:15
- ◯ 00:30
- ◯ 00:45
- ◯ 01:00
- ◯ 01:15
- ◯ 01:30
- ◯ 01:45
- ◯ 02:00
- ◯ 02:15
- ◯ 02:30
- ◯ 02:45
- ◯ 03:00
- ◯ 03:15
- ◯ 03:30
- ◯ 03:45
- ◯ 04:00
- ◯ 04:15
- ◯ 04:30
- ◯ 04:45
- ◯ 05:00
- ◯ 05:15
- ◯ 05:30
- ◯ 05:45
- ◯ 06:00
- ◯ 06:15
- ◯ 06:30
- ◯ 06:45
- ◯ 07:00

- 07:15
- 07:30
- 07:45
- 08:00
- 08:15
- 08:30
- 08:45
- 09:00
- 09:15
- 09:30
- 09:45
- 10:00

8. Nehmen Sie auch an sportlichen Wettkämpfen teil? *

Beantworten Sie diese Frage nur, wenn folgende Bedingungen erfüllt sind:
Antwort war 'Ja' bei Frage '18 [BetreibenSieSport]' (1. Betreiben Sie Sport in Ihrer Freizeit?)

Bitte wählen Sie nur eine der folgenden Antworten aus:

- Ja
- Nein

8.1 Ihr Ziel bei einem sportlichen Wettkampf? *

Beantworten Sie diese Frage nur, wenn folgende Bedingungen erfüllt sind:
Antwort war 'Ja' bei Frage '18 [BetreibenSieSport]' (1. Betreiben Sie Sport in Ihrer Freizeit?) *und* Antwort war 'Ja' bei Frage '34 [Wettkaempfen]' (8. Nehmen Sie auch an sportlichen Wettkämpfen teil?)

Bitte wählen Sie nur eine der folgenden Antworten aus:

- ◯ Durchkommen/Dabei sein ist alles!
- ◯ Freude und Spaß
- ◯ Mannschaft/Teamkolleg_innen/Familie unterstützen
- ◯ konkrete Wertung (z. B. Punkte, Zeit)
- ◯ konkrete Platzierung (z. B. Sieg, Podium, Top 10)
- ◯ Sonstiges [ ]

9. Sind Sie Mitglied in einem Sportverein? *

Beantworten Sie diese Frage nur, wenn folgende Bedingungen erfüllt sind:
Antwort war 'Ja' bei Frage '18 [BetreibenSieSport]' (1. Betreiben Sie Sport in Ihrer Freizeit?)

Bitte wählen Sie nur eine der folgenden Antworten aus:

- ◯ Ja
- ◯ Nein

TEIL C – ERNÄHRUNG

1. Wie ernähren Sie sich aktuell?
Anmerkung:
Definition Ernährungsformen in Anlehnung an die weltweit größte Fachgesellschaft für Ernährung, der Academy of Nutrition and Dietetics (AND, 2015/2016):

Art der Ernährung	**JA, diese Produkte esse ich:**	**NEIN, diese Produkte esse ich NICHT:**
**Mischkost** (ich esse alles)	**„Alles"** z. B. Fleisch inkl. Wurst, Fleischkäse/Leberkäse, Schinken, Salami, Streichwurst, Käse, Milch, Milchprodukte, Fisch und sog. „Meeresfrüchte", Obst, Gemüse, Kartoffeln, Getreide, usw.	/
**Vegetarisch** (pflanzlich; lakto-ovo-vegetarisch)	**Milch und Milchprodukte, Eier und Eiprodukte** z. B. Käse, Topfen/Quark, Joghurt, Molke/Whey, usw.	**Fleisch, Fisch und sog. „Meeresfrüchte"** inkl. Wurst, Schinken, Salami, Fleischkäse/Leberkäse, Streichwurst, usw.
**Vegan** (rein pflanzlich)	**Pflanzliche Produkte** z. B. Obst, Gemüse, Getreide, Samen, Nüsse, Pflanzenvleisch, Pflanzenmilch, Pflanzenkäse usw.	**Keine Produkte vom Tier** z. B. kein Fleisch; keine Eier, Käse, Milch, und Milchprodukte; keinen Honig

*

Bitte wählen Sie nur eine der folgenden Antworten aus:

- ◯ Mischkost (ich esse alles)
- ◯ Vegetarisch (pflanzlich, lakto-ovo-vegetarisch)
- ◯ Vegan (rein pflanzlich)

1.1 Gibt es mindestens 1 vegetarische Speise am Schulbuffet (Jause) bzw. in der Schulkantine (Menü am Mittagstisch)? *

Beantworten Sie diese Frage nur, wenn folgende Bedingungen erfüllt sind:
Antwort war 'Vegetarisch (pflanzlich, lakto-ovo-vegetarisch)' bei Frage '37 [Ernaehrungsform]' (1. Wie ernähren Sie sich aktuell? Anmerkung: Definition Ernährungsformen in Anlehnung an die weltweit größte Fachgesellschaft für Ernährung, der Academy of Nutrition and Dietetics (AND, 2015/2016):)

Bitte wählen Sie nur eine der folgenden Antworten aus:

- Ja
- Nein
- Weiß nicht

1.2 Gibt es mindestens 1 vegane Speise am Schulbuffet (Jause) bzw. in der Schulkantine (Menü am Mittagstisch)? *

Beantworten Sie diese Frage nur, wenn folgende Bedingungen erfüllt sind:
Antwort war 'Vegan (rein pflanzlich)' bei Frage '37 [Ernaehrungsform]' (1. Wie ernähren Sie sich aktuell? Anmerkung: Definition Ernährungsformen in Anlehnung an die weltweit größte Fachgesellschaft für Ernährung, der Academy of Nutrition and Dietetics (AND, 2015/2016):)

Bitte wählen Sie nur eine der folgenden Antworten aus:

- Ja
- Nein
- Weiß nicht

2. Wie lange schon ernähren Sie sich so? *

Bitte wählen Sie nur eine der folgenden Antworten aus:

- ◯ schon immer, mein Leben lang
- ◯ ≤ 0,5 Jahr (1 – 6 Monate)
- ◯ 1 Jahr
- ◯ 2 Jahre
- ◯ 3 – 5 Jahre
- ◯ 6 – 9 Jahre
- ◯ 10 oder mehr Jahre

3. Das ist Ihr Beweggrund für Ihre aktuelle Ernährung, das ist Ihnen wichtig ...
3.1 Das ist Ihr **TOP 1 (wichtigster) Beweggrund** für Ihre aktuelle Ernährung
Anmerkung:
Nur 1 Antwort möglich!

*

Bitte wählen Sie alle zutreffenden Antworten aus:

- [ ] Gesundheit, Wohlbefinden
- [ ] Sportliche Leistung
- [ ] Tierschutz (Tierethik)
- [ ] Klimaschutz/Umweltschutz (ökologische Aspekte)
- [ ] Welthunger (soziale Aspekte, z. B. Land, Nahrung, etc.)
- [ ] Religion/Spiritualität
- [ ] Gewohnheit /Erziehung/Tradition
- [ ] Geschmack/Genuss
- [ ] Lebensmittelskandale
- [ ] Qualität der Nahrungsmittel (z. B. biologischer Anbau)
- [ ] Billige Nahrungsmittel (finanzielle/ökonomische Aspekte)
- [ ] Eltern/Geschwister/Familie
- [ ] Freunde
- [ ] Lehrer_innen/Schulkolleg_innen (Schule)
- [ ] Vorbilder (z. B. Sportler, Popstars, Schauspieler)
- [ ] Veggie Boom/Lifestyle (chic, ‚in‘, dabei-sein, z. B. Medien, Promis)
- [ ] Kein bestimmter Grund
- [ ] Andere

3.1.1 Andere: *

Beantworten Sie diese Frage nur, wenn folgende Bedingungen erfüllt sind:
Antwort war bei Frage '41 [BeweggrundERL1]' (3. Das ist Ihr Beweggrund für Ihre aktuelle Ernährung, das ist Ihnen wichtig ... 3.1 Das ist Ihr TOP 1 (wichtigster) Beweggrund für Ihre aktuelle Ernährung Anmerkung: Nur 1 Antwort möglich!)

Bitte geben Sie Ihre Antwort hier ein:

3. Das ist Ihr Beweggrund für Ihre aktuelle Ernährung, das ist Ihnen wichtig ...
3.2 Das ist Ihr **TOP 2 Beweggrund** für Ihre aktuelle Ernährung
Anmerkung:
Nur 1 Antwort möglich! *

Bitte wählen Sie alle zutreffenden Antworten aus:

- [ ] Gesundheit, Wohlbefinden
- [ ] Sportliche Leistung
- [ ] Tierschutz (Tierethik)
- [ ] Klimaschutz/Umweltschutz (ökologische Aspekte)
- [ ] Welthunger (soziale Aspekte, z. B. Land, Nahrung, etc.)
- [ ] Religion/Spiritualität
- [ ] Gewohnheit /Erziehung/Tradition
- [ ] Geschmack/Genuss
- [ ] Lebensmittelskandale
- [ ] Qualität der Nahrungsmittel (z. B. biologischer Anbau)
- [ ] Billige Nahrungsmittel (finanzielle/ökonomische Aspekte)
- [ ] Eltern/Geschwister/Familie
- [ ] Freunde
- [ ] Lehrer_innen/Schulkolleg_innen (Schule)
- [ ] Vorbilder (z. B. Sportler, Popstars, Schauspieler)
- [ ] Veggie Boom/Lifestyle (chic, ‚in‘, dabei-sein, z. B. Medien, Promis)
- [ ] Kein bestimmter Grund
- [ ] Andere

3.2.1 Andere: *

Beantworten Sie diese Frage nur, wenn folgende Bedingungen erfüllt sind:
Antwort war bei Frage '43 [BeweggrundERL2]' (3. Das ist Ihr Beweggrund für Ihre aktuelle Ernährung, das ist Ihnen wichtig ... 3.2 Das ist Ihr TOP 2 Beweggrund für Ihre aktuelle Ernährung Anmerkung: Nur 1 Antwort möglich!)

Bitte geben Sie Ihre Antwort hier ein:

3. Das ist Ihr Beweggrund für Ihre aktuelle Ernährung, das ist Ihnen wichtig ...
3.3 Das ist Ihr **TOP 3 Beweggrund** für Ihre aktuelle Ernährung
Anmerkung:
Nur 1 Antwort möglich!

*

Bitte wählen Sie alle zutreffenden Antworten aus:

- [ ] Gesundheit, Wohlbefinden
- [ ] Sportliche Leistung
- [ ] Tierschutz (Tierethik)
- [ ] Klimaschutz/Umweltschutz (ökologische Aspekte)
- [ ] Welthunger (soziale Aspekte, z. B. Land, Nahrung, etc.)
- [ ] Religion/Spiritualität
- [ ] Gewohnheit /Erziehung/Tradition
- [ ] Geschmack/Genuss
- [ ] Lebensmittelskandale
- [ ] Qualität der Nahrungsmittel (z. B. biologischer Anbau)
- [ ] Billige Nahrungsmittel (finanzielle/ökonomische Aspekte)
- [ ] Eltern/Geschwister/Familie
- [ ] Freunde
- [ ] Lehrer_innen/Schulkolleg_innen (Schule)
- [ ] Vorbilder (z. B. Sportler, Popstars, Schauspieler)
- [ ] Veggie Boom/Lifestyle (chic, ‚in‘, dabei-sein, z. B. Medien, Promis)
- [ ] Kein bestimmter Grund
- [ ] Andere

3.3.1 Andere: *

Beantworten Sie diese Frage nur, wenn folgende Bedingungen erfüllt sind:
Antwort war bei Frage '45 [BeweggrundERL3]' (3. Das ist Ihr Beweggrund für Ihre aktuelle Ernährung, das ist Ihnen wichtig ... 3.3 Das ist Ihr TOP 3 Beweggrund für Ihre aktuelle Ernährung Anmerkung: Nur 1 Antwort möglich!)

Bitte geben Sie Ihre Antwort hier ein:

4. Wie viel trinken Sie insgesamt pro Tag (Aufstehen/Frühstück bis zum Schlafen-Gehen)? *Anmerkung:* *0,25 Liter (bzw. 250 ml) entspricht z. B. 1 großen Tasse Tee;* *0,5 Liter (bzw. 500 ml) entspricht z. B. 1 kleinen PET-Flasche Eistee oder 1 großem Glas Apfelsaft.*

*

Bitte wählen Sie nur eine der folgenden Antworten aus:

- weniger als 1 Liter/Tag
- zwischen 1 – 1,5 Liter/Tag
- zwischen 1,5 – 2 Liter/Tag
- zwischen 2 – 2,5 Liter/Tag
- mehr als 2,5 Liter/Tag

5. Was ist Ihr Lieblingsgetränk? *

Bitte wählen Sie nur eine der folgenden Antworten aus:

- ◯ Wasser (Leitungswasser, Mineralwasser, Sodawasser)
- ◯ Fruchtsäfte (verdünnt und unverdünnt, z. B. Apfelsaft, Orangensaft)
- ◯ Verdünnungssäfte (z. B. aus Himbeersirup, Holundersirup)
- ◯ Soft-Drinks (zuckerhaltige Getränke inkl. Light- und Zero-Produkte wie z. B. Eistee, Cola, Fanta, Sprite, Almdudler, usw.)
- ◯ Tee (heiß oder kalt)
- ◯ Kakao/heiße Schokolade
- ◯ Kaffee
- ◯ Energy Drinks (z. B. Red Bull, Flying Horse, etc.)
- ◯ andere ____________________

6. Was trinken Sie am häufigsten, z. B. täglich? *

Bitte wählen Sie nur eine der folgenden Antworten aus:

- ◯ Wasser (Leitungswasser, Mineralwasser, Sodawasser)
- ◯ Fruchtsäfte (verdünnt und unverdünnt, z. B. Apfelsaft, Orangensaft)
- ◯ Verdünnungssäfte (z. B. aus Himbeersirup, Holundersirup)
- ◯ Soft-Drinks (zuckerhaltige Getränke inkl. Light- und Zero-Produkte wie z. B. Eistee, Cola, Fanta, Sprite, Almdudler, usw.)
- ◯ Tee (heiß oder kalt)
- ◯ Kakao/heiße Schokolade
- ◯ Kaffee
- ◯ Energy Drinks (z. B. Red Bull, Flying Horse, etc.)
- ◯ andere ____________________

7. Essen Sie täglich Obst? *

Bitte wählen Sie nur eine der folgenden Antworten aus:

- ◯ Ja
- ◯ Nein

8. Essen Sie täglich Gemüse? *

Bitte wählen Sie nur eine der folgenden Antworten aus:

- ◯ Ja
- ◯ Nein

TEIL D – Gesundheit

1. Welche Nahrungsmittel – denken Sie – sind gesund und fördern Ihre Gesundheit?
1.1 Tierische Nahrungsmittel
Anmerkung:
Mehrfachnennung möglich!

Bitte wählen Sie alle zutreffenden Antworten aus:

- ☐ Fleisch und Wurst, z. B. Schnitzel, Streichwurst, Fleischkäse/Leberkäse, Frankfurter Würstchen, Salami, Schinken, etc.
- ☐ Fisch und sog. „Meeresfrüchte“, z. B. Muscheln, Scampi, Fischöl, etc.
- ☐ Milch und Milchprodukte, z. B. Käse, Butter, Joghurt, Topfen/Quark, Sahne, etc.
- ☐ Eier
- ☐ Keine

1. Welche Nahrungsmittel – denken Sie – sind gesund und fördern Ihre Gesundheit?
1.2 Pflanzliche Nahrungsmittel
Anmerkung:
Mehrfachnennung möglich!

Bitte wählen Sie alle zutreffenden Antworten aus:

- ☐ Obst
- ☐ Gemüse
- ☐ Getreideprodukte, z. B. Brot, Nudeln, Reis, Müsli, etc.
- ☐ Kartoffeln und Hülsenfrüchte, z. B. Bohnen, Erbsen, etc.
- ☐ Keine

1. Welche Nahrungsmittel – denken Sie – sind gesund und fördern Ihre Gesundheit?
1.3 Nahrungsergänzungsmittel bzw. Supplemente, für z. B. Vitamine, Mineralstoffe und Spurenelemente, Protein, Ballaststoffe, etc. in Form von Tabletten/Pillen und Pulver *

Bitte wählen Sie nur eine der folgenden Antworten aus:

- ◯ Ja
- ◯ Nein
- ◯ Weiß nicht
- ◯ Manchmal (z. B. bei Mängeln, Krankheit oder wenn man viel Sport treibt)

1. Welche Nahrungsmittel – denken Sie – sind gesund und fördern Ihre Gesundheit?
1.4 Andere Faktoren:
Anmerkung:
Mehrfachnennung möglich!

Bitte wählen Sie alle zutreffenden Antworten aus:

- ☐ Schlaf (viel, guter)
- ☐ Kein Druck und Stress
- ☐ Flüssigkeit (viel)
- ☐ Energydrinks
- ☐ Andere: ____________

1. Welche Nahrungsmittel – denken Sie – fördern Ihre Gesundheit am meisten?
1.5.1 Das ist Ihr **TOP 1 (wichtigster) Faktor** für Ihre Gesundheit
Anmerkung:
Nur 1 Antwort möglich! *

Bitte wählen Sie alle zutreffenden Antworten aus:

- ☐ Fleisch und Wurst, z. B. Schnitzel, Streichwurst, Fleischkäse/Leberkäse, Frankfurter Würstchen, Salami, Schinken, etc.
- ☐ Fisch und sog. „Meeresfrüchte“, z. B. Muscheln, Scampi, Fischöl, etc.
- ☐ Milch und Milchprodukte, z. B. Käse, Butter, Joghurt, Topfen/Quark, Sahne, etc.
- ☐ Eier
- ☐ Obst
- ☐ Gemüse
- ☐ Getreideprodukte, z. B. Brot, Nudeln, Reis, Müsli, etc.
- ☐ Kartoffeln und Hülsenfrüchte, z. B. Bohnen, Erbsen, etc.
- ☐ Nahrungsergänzungsmittel bzw. Supplemente, für z. B. Vitamine, Mineralstoffe und Spurenelemente, Protein, Ballaststoffe, etc. in Form von Tabletten/Pillen und Pulver
- ☐ Schlaf (viel, guter)
- ☐ Kein Druck und Stress
- ☐ Flüssigkeit (viel)
- ☐ Energydrinks
- ☐ Andere

1.5.1.1 Andere: *

Beantworten Sie diese Frage nur, wenn folgende Bedingungen erfüllt sind:
Antwort war bei Frage '56 [NahrungsmTop1]' (1. Welche Nahrungsmittel – denken Sie – fördern Ihre Gesundheit am meisten? 1.5.1 Das ist Ihr TOP 1 (wichtigster) Faktor für Ihre Gesundheit Anmerkung: Nur 1 Antwort möglich!)

Bitte geben Sie Ihre Antwort hier ein:

1. Welche Nahrungsmittel – denken Sie – fördern Ihre Gesundheit am meisten?
1.5.2 Das ist Ihr **TOP 2 Faktor** für Ihre Gesundheit
Anmerkung:
Nur 1 Antwort möglich! *

Bitte wählen Sie alle zutreffenden Antworten aus:

- ☐ Fleisch und Wurst, z. B. Schnitzel, Streichwurst, Fleischkäse/Leberkäse, Frankfurter Würstchen, Salami, Schinken, etc.
- ☐ Fisch und sog. „Meeresfrüchte“, z. B. Muscheln, Scampi, Fischöl, etc.
- ☐ Milch und Milchprodukte, z. B. Käse, Butter, Joghurt, Topfen/Quark, Sahne, etc.
- ☐ Eier
- ☐ Obst
- ☐ Gemüse
- ☐ Getreideprodukte, z. B. Brot, Nudeln, Reis, Müsli, etc.
- ☐ Kartoffeln und Hülsenfrüchte, z. B. Bohnen, Erbsen, etc.
- ☐ Nahrungsergänzungsmittel bzw. Supplemente, für z. B. Vitamine, Mineralstoffe und Spurenelemente, Protein, Ballaststoffe, etc. in Form von Tabletten/Pillen und Pulver
- ☐ Schlaf (viel, guter)
- ☐ Kein Druck und Stress
- ☐ Flüssigkeit (viel)
- ☐ Energydrinks
- ☐ Andere

1.5.2.1 Andere: *

Beantworten Sie diese Frage nur, wenn folgende Bedingungen erfüllt sind:
Antwort war bei Frage '58 [NahrungsmTop2]' (1. Welche Nahrungsmittel – denken Sie – fördern Ihre Gesundheit am meisten? 1.5.2 Das ist Ihr TOP 2 Faktor für Ihre Gesundheit Anmerkung: Nur 1 Antwort möglich!)

Bitte geben Sie Ihre Antwort hier ein:

1. Welche Nahrungsmittel – denken Sie – fördern Ihre Gesundheit am meisten?

1.5.3 Das ist Ihr **TOP 3 Faktor** für Ihre Gesundheit

Anmerkung:
Nur 1 Antwort möglich! *

Bitte wählen Sie alle zutreffenden Antworten aus:

- [ ] Fleisch und Wurst, z. B. Schnitzel, Streichwurst, Fleischkäse/Leberkäse, Frankfurter Würstchen, Salami, Schinken, etc.
- [ ] Fisch und sog. „Meeresfrüchte“, z. B. Muscheln, Scampi, Fischöl, etc.
- [ ] Milch und Milchprodukte, z. B. Käse, Butter, Joghurt, Topfen/Quark, Sahne, etc.
- [ ] Eier
- [ ] Obst
- [ ] Gemüse
- [ ] Getreideprodukte, z. B. Brot, Nudeln, Reis, Müsli, etc.
- [ ] Kartoffeln und Hülsenfrüchte, z. B. Bohnen, Erbsen, etc.
- [ ] Nahrungsergänzungsmittel bzw. Supplemente, für z. B. Vitamine, Mineralstoffe und Spurenelemente, Protein, Ballaststoffe, etc. in Form von Tabletten/Pillen und Pulver
- [ ] Schlaf (viel, guter)
- [ ] Kein Druck und Stress
- [ ] Flüssigkeit (viel)
- [ ] Energydrinks
- [ ] Andere

1.5.3.1 Andere: *

Beantworten Sie diese Frage nur, wenn folgende Bedingungen erfüllt sind:
Antwort war bei Frage '60 [NahrungsmTop3]' (1. Welche Nahrungsmittel – denken Sie – fördern Ihre Gesundheit am meisten? 1.5.3 Das ist Ihr TOP 3 Faktor für Ihre Gesundheit Anmerkung: Nur 1 Antwort möglich!)

Bitte geben Sie Ihre Antwort hier ein:

____

2. Wie – denken Sie – ist Bewegung & Sport gesund und fördert Ihre Gesundheit?
2.1 Häufigkeit: Wie oft – denken Sie – ist Bewegung & Sport gesund und fördert Ihre Gesundheit? *

Bitte wählen Sie nur eine der folgenden Antworten aus:

- ◯ nie
- ◯ ab und zu, gelegentlich
- ◯ regelmäßig

2.1.1 Häufigkeit: ab und zu, gelegentlich, z. B. *

Beantworten Sie diese Frage nur, wenn folgende Bedingungen erfüllt sind:
Antwort war 'ab und zu, gelegentlich' bei Frage '62 [BUSHaeufigkeit]' (2. Wie – denken Sie – ist Bewegung & Sport gesund und fördert Ihre Gesundheit? 2.1 Häufigkeit: Wie oft – denken Sie – ist Bewegung & Sport gesund und fördert Ihre Gesundheit?)

Bitte wählen Sie nur eine der folgenden Antworten aus:

- ◯ 1mal pro Monat
- ◯ 2mal pro Monat
- ◯ 1mal pro Woche
- ◯ Sonstiges ____________

2.1.2 Häufigkeit: regelmäßig *

Beantworten Sie diese Frage nur, wenn folgende Bedingungen erfüllt sind:
Antwort war 'regelmäßig' bei Frage '62 [BUSHaeufigkeit]' (2. Wie – denken Sie – ist Bewegung & Sport gesund und fördert Ihre Gesundheit? 2.1 Häufigkeit: Wie oft – denken Sie – ist Bewegung & Sport gesund und fördert Ihre Gesundheit?)

Bitte wählen Sie nur eine der folgenden Antworten aus:

- ◯ 5mal pro Woche
- ◯ 4mal pro Woche
- ◯ 3mal pro Woche
- ◯ 2mal pro Woche
- ◯ täglich
- ◯ Sonstiges

2. Wie – denken Sie – ist Bewegung & Sport gesund und fördert Ihre Gesundheit?
2.2 Dauer: Wie lange – denken Sie – soll Bewegung & Sport dauern, damit es gesund und förderlich für Ihre Gesundheit ist? *

Bitte wählen Sie nur eine der folgenden Antworten aus:

- ◯ weniger als 20 min/Tag reichen aus
- ◯ mindestens 20 min/Tag
- ◯ etwa 1 h/Tag
- ◯ mindestens 1 h/Tag
- ◯ Sonstiges

2. Wie – denken Sie – ist Bewegung & Sport gesund und fördert Ihre Gesundheit? 2.3 Intensität: Wie intensiv – denken Sie – soll Bewegung & Sport sein, damit es gesund und förderlich für Ihre Gesundheit ist? *

Bitte wählen Sie nur eine der folgenden Antworten aus:

- ◯ locker, niedrige Intensität
- ◯ gefordert, nicht zu locker/nicht zu anstrengend, mittlere Intensität
- ◯ anstrengend, hohe Intensität
- ◯ Sonstiges

3. Was – denken Sie – ist der wichtigste (Haupt-)Faktor für Ihre Gesundheit? *

Bitte wählen Sie nur eine der folgenden Antworten aus:

- ◯ Bewegung & Sport alleine
- ◯ Ernährung alleine
- ◯ abwechselnd achten auf: ab und zu Bewegung & Sport, ab und zu gesunde Ernährung
- ◯ Bewegung & Sport immer kombiniert mit Ernährung
- ◯ Medikamente & Therapie (vom Arzt verschrieben)
- ◯ Sonstiges

4. Leiden Sie an Lebensmittel-/Nahrungsmittel-Allergien oder -Intoleranzen? *

Bitte wählen Sie nur eine der folgenden Antworten aus:

- ◯ Ja
- ◯ Nein

4.1 Welche Lebensmittel-/Nahrungsmittel-Allergien oder -Intoleranzen haben Sie?

Beantworten Sie diese Frage nur, wenn folgende Bedingungen erfüllt sind:
Antwort war 'Ja' bei Frage '68 [LebensmAllergien]' (4. Leiden Sie an Lebensmittel-/Nahrungsmittel-Allergien oder -Intoleranzen?)

Bitte geben Sie Ihre Antwort hier ein:

5. Wie würden Sie aktuell Ihren allgemeinen Gesundheitszustand einschätzen?

Anmerkung:
In Anlehnung an den LBIHPR Forschungsbericht (Hoffmann et al. 2012).

*

Bitte wählen Sie nur eine der folgenden Antworten aus:

- ◯ ausgezeichnet
- ◯ sehr gut
- ◯ gut
- ◯ weniger gut
- ◯ schlecht

TEIL E – SONSTIGES zum Abschluss

1. Essen Sie folgende Lebensmittel?
Anmerkung:
Mehrfachnennung möglich! *

Bitte wählen Sie alle zutreffenden Antworten aus:

- ☐ Obst
- ☐ Gemüse
- ☐ Getreideprodukte, z. B. Brot, Nudeln, Reis, Müsli, Cornflakes, Seitan, etc.
- ☐ Kartoffeln und Hülsenfrüchte, z. B. Bohnen, Erbsen, Linsen, Tofu, Pommes Frites, etc.
- ☐ Fleisch und Wurst, z. B. Schnitzel, Streichwurst, Fleischkäse/Leberkäse, Frankfurter Würstchen, Salami, Schinken, etc.
- ☐ Fisch und sog. „Meeresfrüchte“, z. B. Muscheln, Scampi, Fischöl, etc.
- ☐ Milch und Milchprodukte, z. B. Käse, Butter, Joghurt, Topfen/Quark, Sahne, etc.
- ☐ Eier
- ☐ Honig

2. Diese Freizeit-Aktivitäten sind Ihnen wichtig ...
2.1 Das ist Ihre **TOP 1 (liebste) Freizeit-Aktivität**
Anmerkung:
Nur 1 Antwort möglich! *

Bitte wählen Sie alle zutreffenden Antworten aus:

- ☐ Fernsehen/Video schauen
- ☐ Musik (z. B. hören, singen, Instrument spielen, Lyrics schreiben)
- ☐ Computer spielen/Internet surfen
- ☐ Sport treiben
- ☐ Lesen/Schreiben
- ☐ Freunde treffen (z. B. Bummeln, Chillen, Shoppen gehen, etc.)
- ☐ Basteln, Malen, Handarbeiten, etc.
- ☐ andere

2.1.1 Andere: *

Beantworten Sie diese Frage nur, wenn folgende Bedingungen erfüllt sind:
Antwort war bei Frage '72 [FreizeitAktivitaet1]' (2. Diese Freizeit-Aktivitäten sind Ihnen wichtig ... 2.1 Das ist Ihre TOP 1 (liebste) Freizeit-Aktivität Anmerkung: Nur 1 Antwort möglich!)

Bitte geben Sie Ihre Antwort hier ein:

2. Diese Freizeit-Aktivitäten sind Ihnen wichtig ...
2.2 Das ist Ihre **TOP 2 Freizeit-Aktivität**
Anmerkung:
Nur 1 Antwort möglich! *

Bitte wählen Sie alle zutreffenden Antworten aus:

- ☐ Fernsehen/Video schauen
- ☐ Musik (z. B. hören, singen, Instrument spielen, Lyrics schreiben)
- ☐ Computer spielen/Internet surfen
- ☐ Sport treiben
- ☐ Lesen/Schreiben
- ☐ Freunde treffen (z. B. Bummeln, Chillen, Shoppen gehen, etc.)
- ☐ Basteln, Malen, Handarbeiten, etc.
- ☐ andere

2.2.1 Andere: *

Beantworten Sie diese Frage nur, wenn folgende Bedingungen erfüllt sind:
Antwort war bei Frage '74 [FreizeitAktivitaet2]' (2. Diese Freizeit-Aktivitäten sind Ihnen wichtig ... 2.2 Das ist Ihre TOP 2 Freizeit-Aktivität Anmerkung: Nur 1 Antwort möglich!)

Bitte geben Sie Ihre Antwort hier ein:

2. Diese Freizeit-Aktivitäten sind Ihnen wichtig ...
2.3 Das ist Ihre **TOP 3 Freizeit-Aktivität**

Anmerkung:
Nur 1 Antwort möglich! *

Bitte wählen Sie alle zutreffenden Antworten aus:

- ☐ Fernsehen/Video schauen
- ☐ Musik (z. B. hören, singen, Instrument spielen, Lyrics schreiben)
- ☐ Computer spielen/Internet surfen
- ☐ Sport treiben
- ☐ Lesen/Schreiben
- ☐ Freunde treffen (z. B. Bummeln, Chillen, Shoppen gehen, etc.)
- ☐ Basteln, Malen, Handarbeiten, etc.
- ☐ andere

2.3.1 Andere: *

Beantworten Sie diese Frage nur, wenn folgende Bedingungen erfüllt sind:
Antwort war bei Frage '76 [FreizeitAktivitaet3]' (2. Diese Freizeit-Aktivitäten sind Ihnen wichtig ... 2.3 Das ist Ihre TOP 3 Freizeit-Aktivität Anmerkung: Nur 1 Antwort möglich!)

Bitte geben Sie Ihre Antwort hier ein:

3. Rauchen Sie Zigaretten? *

Bitte wählen Sie nur eine der folgenden Antworten aus:

- ○ Ja
- ○ Nein

3.1 Wie oft rauchen Sie? *

Beantworten Sie diese Frage nur, wenn folgende Bedingungen erfüllt sind:
Antwort war 'Ja' bei Frage '78 [Rauchen]' (3. Rauchen Sie Zigaretten?)

Bitte wählen Sie nur eine der folgenden Antworten aus:

- ◯ täglich
- ◯ regelmäßig, aber nicht täglich
- ◯ gelegentlich

3.1.1 Wie viele Zigaretten rauchen Sie täglich?

Beantworten Sie diese Frage nur, wenn folgende Bedingungen erfüllt sind:
Antwort war 'täglich' bei Frage '79 [RauchenHauefigkeit]' (3.1 Wie oft rauchen Sie?)

Bitte wählen Sie nur eine der folgenden Antworten aus:

- ◯ 1
- ◯ 2
- ◯ 3
- ◯ 4
- ◯ 5
- ◯ 6
- ◯ 7
- ◯ 8
- ◯ 9
- ◯ 10
- ◯ 11
- ◯ 12
- ◯ 13
- ◯ 14
- ◯ 15
- ◯ 16
- ◯ 17
- ◯ 18
- ◯ 19
- ◯ 20
- ◯ 21
- ◯ 22
- ◯ 23
- ◯ 24
- ◯ 25
- ◯ 26
- ◯ 27
- ◯ 28
- ◯ 29

- ◯ 30
- ◯ 31
- ◯ 32
- ◯ 33
- ◯ 34
- ◯ 35
- ◯ 36
- ◯ 37
- ◯ 38
- ◯ 39
- ◯ 40
- ◯ 41
- ◯ 42
- ◯ 43
- ◯ 44
- ◯ 45
- ◯ 46
- ◯ 47
- ◯ 48
- ◯ 49
- ◯ 50
- ◯ 51
- ◯ 52
- ◯ 53
- ◯ 54
- ◯ 55
- ◯ 56
- ◯ 57
- ◯ 58
- ◯ 59
- ◯ 60
- ◯ 61
- ◯ 62
- ◯ 63
- ◯ 64

- ◯ 65
- ◯ 66
- ◯ 67
- ◯ 68
- ◯ 69
- ◯ 70
- ◯ 71
- ◯ 72
- ◯ 73
- ◯ 74
- ◯ 75
- ◯ 76
- ◯ 77
- ◯ 78
- ◯ 79
- ◯ 80
- ◯ 81
- ◯ 82
- ◯ 83
- ◯ 84
- ◯ 85
- ◯ 86
- ◯ 87
- ◯ 88
- ◯ 89
- ◯ 90
- ◯ 91
- ◯ 92
- ◯ 93
- ◯ 94
- ◯ 95
- ◯ 96
- ◯ 97
- ◯ 98
- ◯ 99

◯ 100

4. Ihr Lifestyle: Was finden Sie wichtig (sinnstiftend bzw. cool) hinsichtlich (Ihres) Lifestyle?
Anmerkung:
Mehrfachnennung möglich! *

Bitte wählen Sie alle zutreffenden Antworten aus:

- ☐ Generell Sport treiben
- ☐ Lifestyle einer bestimmten Sportart, z. B. Tanzen (Kleidung, Philosophie, etc.)
- ☐ Alkohol trinken
- ☐ Zigaretten rauchen
- ☐ Fleisch essen
- ☐ Vegetarisch essen
- ☐ Vegetarischer Lifestyle (Kleidung, Philosophie, etc.)
- ☐ Vegan essen
- ☐ Veganer Lifestyle (Kleidung, Philosophie, etc.)
- ☐ Sonstiges: ____________

4.1 Was finden Sie **am wichtigsten, besonders sinnstiftend bzw. am coolsten (TOP 1)** hinsichtlich (Ihres) Lifestyle?

Anmerkung:
Nur 1 Antwort möglich! *

Bitte wählen Sie alle zutreffenden Antworten aus:

- ☐ Generell Sport treiben
- ☐ Lifestyle einer bestimmten Sportart, z. B. Tanzen (Kleidung, Philosophie, etc.)
- ☐ Alkohol trinken
- ☐ Zigaretten rauchen
- ☐ Fleisch essen
- ☐ Vegetarisch essen
- ☐ Vegetarischer Lifestyle (Kleidung, Philosophie, etc.)
- ☐ Vegan essen
- ☐ Veganer Lifestyle (Kleidung, Philosophie, etc.)
- ☐ Sonstiges

4.2 Was finden Sie **wichtig, sinnstiftend bzw. cool (TOP 2)** hinsichtlich (Ihres) Lifestyle?

Anmerkung:
Nur 1 Antwort möglich! *

Bitte wählen Sie alle zutreffenden Antworten aus:

- ☐ Generell Sport treiben
- ☐ Lifestyle einer bestimmten Sportart, z. B. Tanzen (Kleidung, Philosophie, etc.)
- ☐ Alkohol trinken
- ☐ Zigaretten rauchen
- ☐ Fleisch essen
- ☐ Vegetarisch essen
- ☐ Vegetarischer Lifestyle (Kleidung, Philosophie, etc.)
- ☐ Vegan essen
- ☐ Veganer Lifestyle (Kleidung, Philosophie, etc.)
- ☐ Sonstiges

4.3 Was finden Sie **wichtig, sinnstiftend bzw. cool (TOP 3)** hinsichtlich (Ihres) Lifestyle?
Anmerkung:
Nur 1 Antwort möglich! *

Bitte wählen Sie alle zutreffenden Antworten aus:

- ☐ Generell Sport treiben
- ☐ Lifestyle einer bestimmten Sportart, z. B. Tanzen (Kleidung, Philosophie, etc.)
- ☐ Alkohol trinken
- ☐ Zigaretten rauchen
- ☐ Fleisch essen
- ☐ Vegetarisch essen
- ☐ Vegetarischer Lifestyle (Kleidung, Philosophie, etc.)
- ☐ Vegan essen
- ☐ Veganer Lifestyle (Kleidung, Philosophie, etc.)
- ☐ Sonstiges

5. Trinken Sie Alkohol? *

Bitte wählen Sie nur eine der folgenden Antworten aus:

- ◯ Ja
- ◯ Nein, ich trinke nie

5.1 Wie oft trinken Sie Alkohol? *

Beantworten Sie diese Frage nur, wenn folgende Bedingungen erfüllt sind:
Antwort war 'Ja' bei Frage '85 [Alkohol]' (5. Trinken Sie Alkohol?)

Bitte wählen Sie nur eine der folgenden Antworten aus:

- ◯ täglich
- ◯ regelmäßig, aber nicht täglich
- ◯ gelegentlich

Vielen Dank für Deine Teilnahme an der Schulstudie
‚From Science 2 School: Nachhaltig gesund – bewegt & veggie'.

Dein Fragebogen wurde erfolgreich abgesendet.

Übermittlung Ihres ausgefüllten Fragebogens:
Vielen Dank für die Beantwortung des Fragebogens.
